# A meta-learning method with reduced data requirements for training and updating deep learning models on wearable devices

**DOI:** 10.1038/s43856-026-01740-1

**Published:** 2026-07-31

**Authors:** Alireza Amirshahi, Maedeh H. Toosi, Siamak Mohammadi, Stefano Albini, Pasquale Davide Schiavone, Giovanni Ansaloni, Amir Aminifar, David Atienza

**Affiliations:** 1 Embedded Systems Laboratory (ESL), École Polytechnique Fédérale de Lausanne (EPFL), Lausanne, Switzerland; 2 School of Electrical and Computer Engineering, University of Tehran, Tehran, Iran; 3 School of Computing Science, Institute for Research in Fundamental Sciences (IPM), Tehran, Iran; 4 Department of Electrical and Information Technology, Lund University, Lund, Sweden

**Keywords:** Electroencephalography - EEG, Atrial fibrillation, Epilepsy, Pathology

## Abstract

**Background:**

Wearable systems provide continuous health monitoring and can lead to early detection of potential health issues. However, the lifecycle of wearable systems faces several challenges. First, effective model training for new wearable devices requires substantial labeled data from various subjects collected directly by the wearable. Second, subsequent model updates require further extensive labeled data for retraining. Finally, frequent model updating on the wearable device can decrease the battery life in long-term data monitoring.

**Methods:**

Addressing these challenges, in this paper, we propose a meta-learning method to reduce the amount of initial data collection required. Moreover, our approach incorporates a prototypical updating mechanism, simplifying the update process by modifying the class prototype rather than retraining the entire model. We explore the performance of our proposed method in two case studies, namely, the detection of epileptic seizures and the detection of atrial fibrillation (AF).

**Results:**

We show that by fine-tuning with just a few samples, we achieve 70% and 82% AUC for the detection of epileptic seizures and the detection of AF, respectively. Compared to a conventional approach, our proposed method performs better with up to 45% AUC. Furthermore, updating the model with only 16 minutes of additional labeled data increases the AUC by up to 5.3%. Finally, the proposed method reduces the energy consumption for model updates by 456x and 418x for epileptic seizure and AF detection, respectively.

**Conclusions:**

Our findings demonstrate that the proposed method effectively overcomes the critical barriers of data scarcity and energy constraints, offering a practical and efficient solution for the lifecycle management of deep learning models on wearable health devices.

## Plain Language Summary

Wearable devices are essential for continuous health monitoring, but introducing a new device presents significant challenges. Training an artificial intelligence (AI) model for the new device typically requires collecting large amounts of medical data specifically from that new hardware. Furthermore, updating these models over time requires continuous data collection and significantly drains the device’s battery. To address these barriers, we developed a method that allows a new device to drastically reduce the amount of data needed for initial training and subsequent updates. In tests for detecting epileptic seizures and irregular heart rhythms, our method achieved high accuracy with very few samples and reduced the energy required for updates by over 400 times. This technology could accelerate the deployment of new health monitors.

## Abstract (web summary)

Amirshahi, Toosi et al. describe MetaWearS, a meta-learning framework designed to overcome labeled data scarcity and high battery consumption in wearable health monitors. The framework achieves up to 93% AUC for abnormality detection and reduces the energy required for model updates by over 400 times compared to conventional approaches.

## Introduction

Advancements in biomedical signal monitoring techniques have contributed significantly to the early diagnosis and treatment of various medical conditions. Immediate and accurate detection of these conditions can guide appropriate medical interventions, thereby improving patient care^1,2^.

Wearable systems are increasingly being used to monitor health conditions and provide real-time data on various physiological parameters. Wearable devices, such as smartwatches, fitness trackers, and specialized medical wearables, can track and record data related to heart rate, blood pressure, sleep patterns, physical activity, etc.^3–5^.

**Fig. 1:**
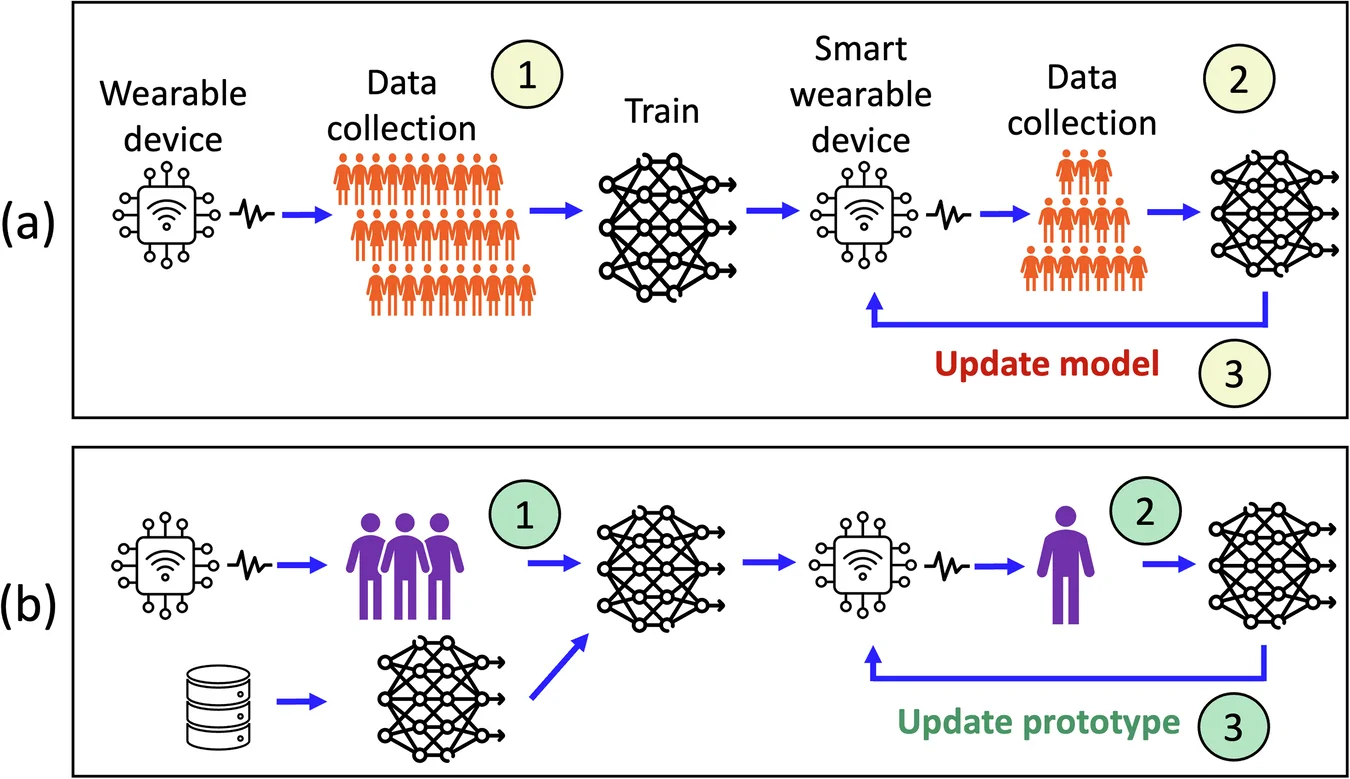
Identifying and resolving critical barriers in the lifecycle of deep learning models on wearable devices. **a** Key challenges in standard lifecycles include ① Extensive initial data collection, ② further data collection for updates, and ③ inefficient update protocol. **b** The proposed method addresses all the mentioned challenges.

Deep learning has demonstrated significant advances in a variety of biomedical applications. However, implementing these advances into practical wearable systems often faces numerous challenges. These challenges span the entire lifecycle of a wearable device, from its data collection phase to its utilization by end-users. Figure 1a shows the definition of a wearable system lifecycle and its challenges. The first challenge, shown in Fig. 1a with ①, is the requirement for substantial biomedical labeled data to train deep learning models. Data collection is a costly and time-consuming process^6^. This issue is further exacerbated in wearable systems by the fact that the data beneficial to the model training should ideally be collected from identical devices^7–10^.

The next challenge for deep learning deployment in wearable systems is the data requirement for the updating process, shown by ② in Fig. 1a. In this phase, data should ideally be collected from a large number of new participants to have enough data to retrain the model. Otherwise, with a limited number of data, the model can suffer from an overfitting problem, where it becomes too fit to the new training data and performs poorly on unseen data^11,12^.

Finally, once new signals are obtained and the model is updated, the updated model should be sent back and replaced on the wearable system. However, deep learning models are typically large. When models are transmitted and read by processors from a wireless network, model transmission can be quite time-consuming and energy-intensive^13^. This significantly impacts battery life, a critical factor for wearable devices. As depicted in Fig. 1a by ③, the third challenge is a high energy consumption for frequent model updates.

In our paper, we introduce MetaWearS, a purpose-built Meta-learning method for Wearable Systems that addresses the critical challenges of data scarcity and resource efficiency. Remarkably, as shown in Fig. 1b, our approach reduces the data required for the initial training of the model (①) and for the update process (②). This is achieved through a targeted modification of the few-shot learning strategy, specifically designed to address the aforementioned challenges. Specifically, MetaWearS reduces the required amount of target-specific labeled data (i.e., data collected directly from the end-user’s wearable device, which is often scarce) by effectively leveraging knowledge transferred from a separate, potentially large and more readily available base dataset (e.g., existing clinical datasets). The use of an extensive base dataset for pre-training is, therefore, key to minimizing the need for target data collection, resolving the apparent contradiction.

Furthermore, our method incorporates an energy-efficient updating mechanism, and instead of retraining and transmitting the entire model, we update only a single vector, called a prototype. In line with the principles of prototypical networks^14^, the prototype size is considerably smaller than the model weight. Therefore, the processor consumes less time and energy to receive the prototypes instead of receiving the whole model (③). This can significantly increase the battery life.

While traditional methods are effective^15^, deep learning offers potential advantages like automatic learning of complex patterns from signals and adaptation to patient variability^16,17^. However, deep learning data and resource demands hinder its use in wearables. MetaWearS is motivated by the goal of overcoming these specific challenges (data scarcity, update efficiency) to make the benefits of advanced deep learning techniques practical for wearable health monitoring.

To evaluate MetaWearS, Epilepsy and AF arrhythmia, two distinct but critical biomedical conditions, are chosen as two case studies based on electroencephalogram (EEG) and electrocardiogram (ECG), respectively, as two different types of biomedical signals. Moreover, we use two types of deep learning models for our study: MobileNetV2^18^ and VisionTransformer^19^. This not only highlights the generality and adaptability of our approach but also emphasizes its potential to surpass the limitations of traditional single-domain evaluations in biomedical signals.

This paper presents four key contributions in the application-based wearable system design for biomedical abnormalities monitoring. First, our work introduces MetaWearS as a few-shot learning strategy specifically designed for the effective detection of abnormalities in biomedical signals, particularly in scenarios where initial labeled data is scarce. Second, the proposed MetaWearS can update the models with empirically minimal additional labeled data when end users are actively using the wearable system. This demonstrates the practical advantages of our methodology in reducing the data requirement. Third, to the best of our knowledge, our work is the first to formalize and evaluate a mechanism where the set of prototypes within a Prototypical Network serves as the sole updatable layer. We specifically evaluate this lightweight update mechanism for its suitability in frequent, on-device personalization scenarios on battery-powered wearable devices. Finally, to assess the generalization capabilities of MetaWearS in the context of few-shot learning, we carried out comprehensive experiments on two separate case studies with two types of biomedical signals: AF arrhythmia detection using ECG and epileptic seizure detection using EEG signals.

## Methods

This section is structured into the following steps. Initially, we describe the datasets and settings used in the case studies of AF arrhythmia detection and epileptic seizure detection in “Data augmentation” and “ECG data for AF detection”, respectively. Next, we provide a conceptual introduction to MetaWearS (“Conceptual introduction of MetaWearS” and “Primary data collection and training”), outlining the meta-learning principles that motivate our framework and explaining how support and query sets, prototypes, and episodic training are defined within our approach. In the subsequent section (“MetaWearS for reducing initial data collection”), we explain how MetaWearS tackles Challenge ①. This challenge pertains to the initial data collection required for training the first model. In “MetaWearS for updating the model using a few new shots”, we employ our proposed few-shot learning method to address Challenge ②, aiming to reduce the demand for a large quantity of annotated new samples for updates during their usage by end-users.

Finally, we address the specific lifecycle challenges: reducing initial data collection (“MetaWearS for reducing initial data collection”, Challenge ①), enabling efficient few-shot updates (“MetaWearS for updating the model using few new shots”, Challenge ②), and optimizing hardware energy consumption (“Hardware architecture in MetaWearS”, Challenge ③).

### EEG data for epileptic seizure detection

The training dataset for the base learner in our study comprises the publicly available Temple University Hospital EEG Seizure Corpus (TUSZ)^20^-v2.0.0, encompassing data from 675 subjects with a cumulative duration of 1476 hours. Notably, the dataset exhibits imbalances, characterized by predominantly short files (average duration of 10 minutes) and a significant preponderance of normal non-seizure signals. Additionally, the dataset features heterogeneity in sampling frequency and the number of channels. To ensure uniformity, all files are resampled to 256 Hz as suggested in ref. ^21^. The spatial arrangement of channels follows the 10–20 system, and we adopt the bipolar montage^22^.

The second database comprises EEG recordings from a limited number of 14 subjects acquired at the Unit of Neurology and Neurophysiology of the University of Siena^23,24^. In particular, four subjects lack seizure data, and, consequently, they are always used in the test set. The original signals were captured at 512 Hz, and we resampled them to 256 Hz for consistency.

#### Data preprocessing and network architecture

The EEG signals in the base and target datasets are filtered by a band-pass filter on [0.5–60] Hz, as well as a 50 Hz notch filter. Subsequently, we extract a short-time Fourier transform (STFT) from each 12-second window of the filtered signal. The STFT employs one-second segmenting, 50 overlapping samples, and a frequency resolution of 2. These parameter choices are extracted from the recommendations in ref. ^25^.

**Fig. 2:**
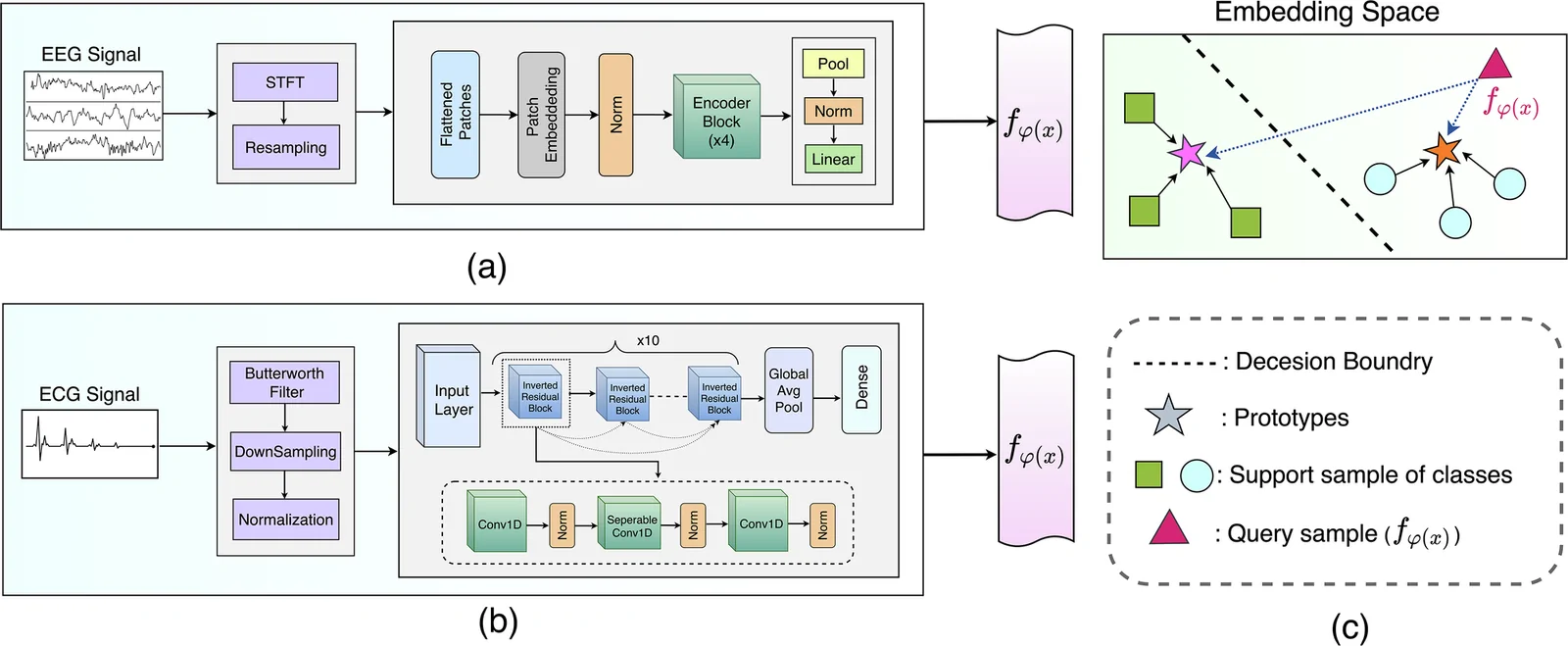
Overview of model architectures for signal classification in wearable health monitoring. **a** The EEG signal processing pathway employs a deep learning-based transformer architecture for Epilepsy detection from EEG signals. **b** The AF detection model utilizes a MobileNetV2 neural network to analyze ECG recordings for real-time classification and **c** after computing the feature vector *f*_*φ*(*x*)_ for each query sample, these vectors are embedded into a high-dimensional space. In this visual representation, data points are classified by computing their Euclidean distances to class prototype vectors.

The model used for this task is a 4-layer VisionTransformer-based model^19^, which is modified for epileptic seizure detection by ref. ^25^. The STFT extracted from the EEG input signal is considered as an input image to this four-layer transformer encoder. Originally, the decoder was implemented as a fully connected layer, reducing the dimensionality to match the number of classes. However, in our approach, using the proposed method, we modify the decoder to employ a fully connected layer, aligning the output dimension with that of the prototypes, which, in this case, is set to 16. Figure 2a displays the preprocessing steps and model architecture that were utilized for the Epilepsy detection.

### ECG data for AF detection

The large-scale dataset used for the base dataset is Physionet Computing in Cardiology Challenge 2017^26^, which consists of 8528 single-lead ECG recordings, divided among AF, normal, noisy, and other classes. We have extracted 7561 signals which have >30 seconds of signal. The dataset consists of 5154 normal recordings, 771 AF recordings, 46 noisy recordings, and 2557 records in the other rhythm classes. These recordings have a sampling frequency of 300 Hz. The duration of ECG recordings varies from 9 seconds to 60 seconds, with a median recording length of 30 seconds.

The second dataset is a private dataset in which data were obtained by a wearable device from patients in a hospital setting. Access to this proprietary data was obtained through a formal agreement with the clinical collaborator, and it remains private to protect participant confidentiality. This dataset contains 303 records of single-lead ECG signals, with each record having a duration of 40 seconds. The sampling rate of the dataset is 200 Hz. This dataset contains ECG signals for normal heart rhythms and AF. From this dataset, 150 ECG samples were selected for fine-tuning, and the remaining data was reserved for testing.

#### Data augmentation

Within our base training dataset, a total of 7561 ECG signals with a recording length of 30 seconds are present. However, this dataset demonstrates a pronounced imbalance, with ~89% of the data affiliated with a specific class, leading to a potential bias towards the class with the most data. In our study, we chose a combined approach involving random resampling and the Gaussian noise method. Initially, random resampling is used to balance the AF and Other classes, rectifying any imbalances. This technique selects samples from the minority class at random and integrates them into the training data. Furthermore, it introduces noise to the synthetic data points, improving variation and smoothing class boundaries, which in turn helps reduce overfitting. After balancing the number of data points in each class, we added amplified noise to only half of the data.

#### Data preprocessing and network architecture

Data preprocessing is an essential step in ECG signal analysis, enhancing data quality, reducing noise, and facilitating reliable analysis and modeling. These preprocessing steps help to improve the quality of the ECG signals and reduce inconsistency across different datasets, as in ref. ^27^. This method involves the application of a second-order band-pass Butterworth filter to eliminate baseline drift and high-frequency noise from ECG signals. Following this, downsampling at 100 Hz is used to ensure uniformity in sampling rates across different datasets. The signals are then normalized using min-max scaling, resulting in values within the range of 0–1.

The CNN structure employed in this study draws inspiration from MobileNetV2 architecture and the architecture in ref. ^27^, where a seven-layer instantiation of MobileNetV2 is optimized for AF detection. Based on the memory capacity in our hardware system, we increased the number of layers to 13 layers.

The employed model consists of ten inverted residual blocks connected in sequence. Each inverted residual block includes a depthwise convolutional layer, followed by a projection layer that reduces the channel size back to the original size. The number of output channels for each inverted residual block is designed to be 8, 12 (x2), 16 (x3), and 24 (x4), respectively. The activation function used in each convolutional layer is the Rectified Linear Unit (ReLU). The model also includes a separable 1D convolutional layer (separable Conv1D) with channel expansion and a pointwise convolutional layer with six times extended channels. The kernel size for the separable Conv1D layer and the inverted residual blocks is set to 7 and 5, respectively. The last layer of the model is a dense layer followed by a ReLU activation function, which performs the classification into the four ECG classes: normal sinus rhythm, AF, atrial premature contraction, and ventricular premature contraction. Overall, the proposed CNN structure is designed to be lightweight and efficient for ECG classification on low-power wearable devices. Figure 2b displays the preprocessing steps and model architecture that were utilized for the AF detection.

Figure 2c illustrates the classification process. First, the prototype vectors are computed. Then each query sample is embedded into a high-dimensional space and classified according to its distances from the prototypes. This highlights the model’s ability to distinguish between classes.

### Ethical approval and data sources

#### Public datasets

The TUSZ v2.0.0 dataset^20^ was created under the oversight and approval of the Temple University Institutional Review Board (IRB). The corpus comprises archival clinical medical records that were rigorously de-identified to conform to the HIPAA Privacy Rule. The Siena Scalp EEG Database^23^ and the PhysioNet Computing in Cardiology Challenge 2017 dataset^26^ are publicly available, de-identified repositories accessed via PhysioNet. The Siena dataset was originally acquired at the Unit of Neurology and Neurophysiology of the University of Siena for clinical purposes and subsequently de-identified for public release. The Ethical Committee of the University of Siena approved the data in accordance with the Declaration of Helsinki. At the time of admission to the clinics, each patient signed a written informed consent in which they agreed to the video registration and to the use of the data for a possible scientific divulgation. The PhysioNet Computing in Cardiology Challenge 2017 dataset, which was provided as a fully de-identified public benchmark, follows PhysioNet’s mandatory vetting process, which requires all data contributors to provide appropriate IRB approval and ethical permissions prior to hosting^28^.

#### Private dataset

The collection and analysis of the target dataset for AF detection were conducted in collaboration with Shahid Modarres Hospital (Tehran, Iran). As the dataset consists of non-invasive, retrospective, and de-identified clinical records originally acquired for routine monitoring purposes, the requirement for specific written informed consent for this study was waived by the Ethics Committee of Shahid Beheshti University of Medical Sciences.

### Conceptual introduction of MetaWearS

In this section, the proposed MetaWearS background is defined in the context of few-shot learning, introducing a distinct methodology that targets the detection of abnormalities in biomedical signals obtained from wearable devices used by a patient. This patient has no prior signals in the training set, and the amount of other annotated signals from the wearable device in the dataset is significantly limited.

**Fig. 3:**
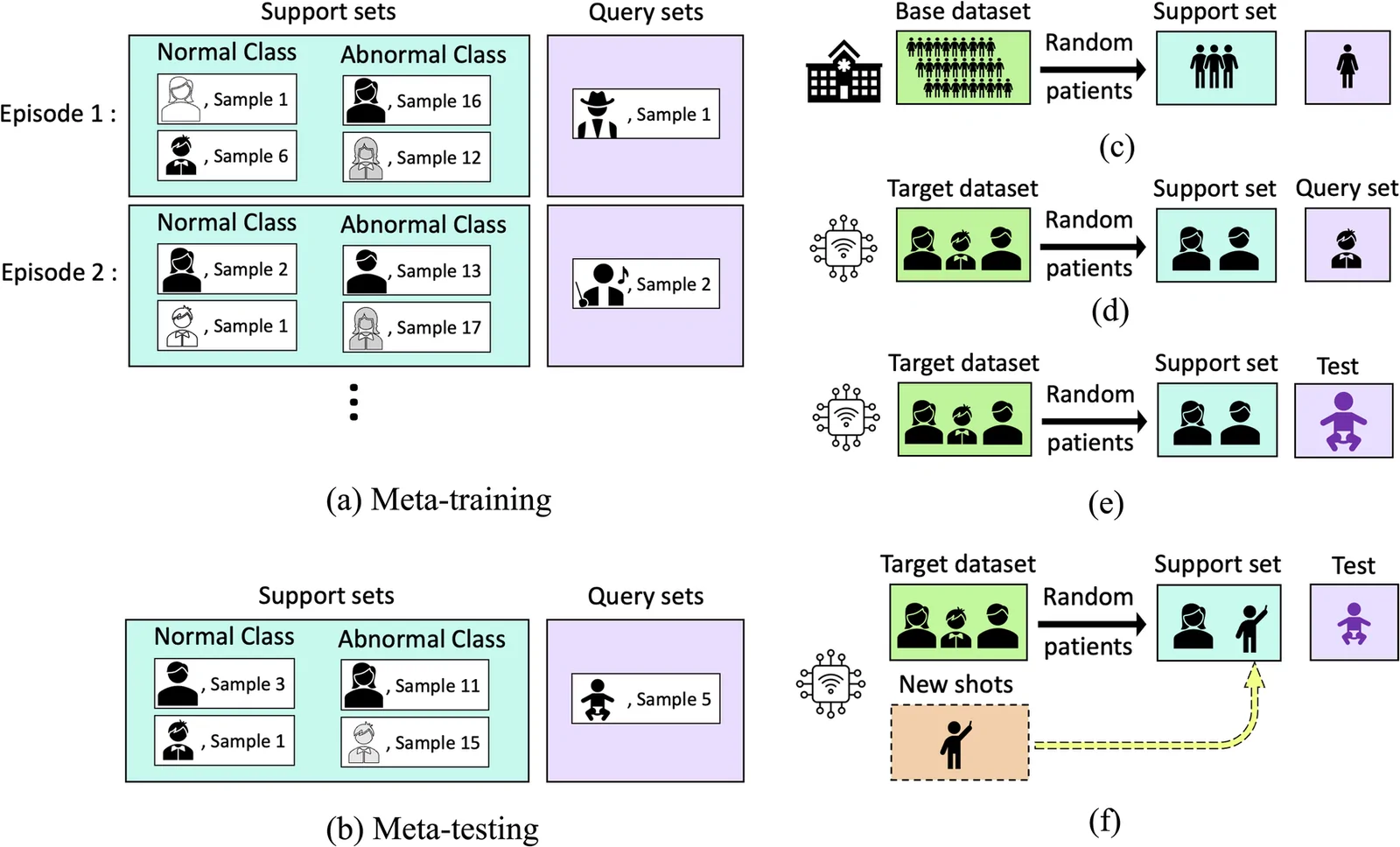
The MetaWearS framework lifecycle, from episodic training to on-device deployment and updates. **a**, **b** Meta-training and meta-testing in MetaWearS. There are three fundamental rules in the definition of episodes: (1) identical classes are always present in each episode; (2) patients in the query set do not have prior samples in the support set; and (3) the support set and the query set are derived from the same device. **c** Pretraining and **d** fine-tuning the model on the meta-train step in MetaWearS. This shows the split of data into a support set and a query set in each episode. **e** Inference process on the wearable systems. Although the amount of labeled data in the target dataset is limited, MetaWearS uses only a few shots for training the model to address Challenge ①. **f** Updating workflow for enhanced wearable system performance using only a few new shots to tackle Challenge ②.

Few-shot learning methods aim at designing a model that classifies a test sample based on a very limited number of previously seen labeled examples. A potential solution for the few-shot learning challenge, in the context of meta-learning, is to simulate the situation of having a few labeled samples during training. Essentially, the model is trained on a large dataset, referred to as the base dataset, to learn the similarities and differences among a few objects. This step of training the model on a base dataset is called meta-training, as shown in Fig. 3a. The training step involves several episodes. In each episode, *k* random samples are chosen from each *N* classes in the base dataset. These samples collectively form a support set for the episode.

Suppose ${{\mathcal{D}}}^{{\rm{train}}}$ is the base dataset for meta-training. The support set for episode *e* is randomly selected from ${{{\mathcal{D}}}}^{{{\rm{train}}}}$ to create ${S}_{e}={\left\{\left({x}_{i}^{s},{y}_{i}^{s}\right)\right\}}_{i=1}^{k\cdot N}$. The goal of this episode is to classify new samples *x*_*j*_ that belong to these *N* classes. Thus, we create a query set with these new samples as ${Q}_{e}={\left\{\left({x}_{j}^{q},{y}_{j}^{q}\right)\right\}}_{j=1}^{m\cdot N}$, where *m* is the number of new samples in each class. The loss function during meta-training aims to minimize the log-likelihood of each predicted class ${\hat{y}}_{j}^{q}$ of the samples in *Q*_*e*_ compared to the corresponding ground truth ${y}_{j}^{q}$.

As depicted in Fig. 3a, the process of creating episodes and selecting samples and classes is repeated multiple times during meta-training. The underlying idea behind meta-training is to simulate a situation where we have a few labeled examples in the support set, and the model needs to classify the class of a new test sample in the query set.

In few-shot learning, a meta-test is created to evaluate the model. This involves entirely new classes that were not present in ${{{\mathcal{D}}}}^{{{\rm{train}}}}$, forming ${{{\mathcal{D}}}}^{{{\rm{test}}}}$, also referred to as the target dataset, which is acquired from a different wearable device than the base dataset in our case. As shown in Fig. 3b, we again have a support set with the same number of classes and samples, and the objective is to classify the samples in the query set. This task mirrors the tasks in the meta-train. During the meta-test phase, we encounter patients who are completely new to the system. Since the model is trained in the meta-training phase with unseen patients in the query set, it is now able to classify the new sample in the meta-test’s query set with only a few shots in the support set.

In the meta-test phase described, where the model encounters entirely new classes from a target dataset, Prototypical Networks^14^ play a crucial role. These networks, adept at handling few-shot scenarios, compute and utilize feature vectors from both support and query sets to efficiently classify new samples, leveraging the previously discussed structure of support and query sets. In each episode, prototypes **c**_*n*_ are calculated for each class *n* within the support set *S*^*n*^ as the average of the feature vectors of all instances belonging to their corresponding classes. The formula for calculating the prototypes is as follows:

1 $${{{\bf{c}}}}_{n}=\frac{1}{\left|{S}^{n}\right|}{\sum }_{\left({{{\bf{x}}}}_{i},{y}_{i}\right)\in {S}^{n}}{f}_{\phi }\left({{{\bf{x}}}}_{i}\right),$$

The query set is also fed into the model, and a feature vector *f*_*ϕ*_(**x**_*j*_) is extracted for each sample **x**_*j*_. The classification of the query set is then performed by comparing the feature vectors of the query set with the class prototypes **c**_*n*_. We can classify the query set by identifying the nearest prototype in this embedding space to the query samples.

While Prototypical Networks effectively manage the task of classifying new samples in the meta-test phase, they rely on a robust meta-training phase that demands a considerable amount of labeled data. This requirement presents a significant challenge, particularly in contexts involving costly data acquisition from wearable devices, leaving Challenge ① unresolved. In the subsequent section, we discuss how MetaWearS addresses this problem.

We chose Prototypical Networks due to their demonstrated effectiveness in few-shot scenarios, their computational efficiency, and their inherent suitability for a lightweight update mechanism based on prototype recalculation, which is central to addressing the energy efficiency challenges in wearable systems.

### Primary data collection and training

In the first phase of a wearable system lifecycle, wearable data must be collected from the patients and must be annotated. These labeled signals form the “target dataset," in which all the signals are derived from the target wearable system. We assume that the amount of labeled signals is too small for training a model. In this section, we tackle Challenge ① to meta-train the model only with a minimal amount of annotated signals in the target dataset.

In our work, we use a “base dataset", which contains a large number of signals with the same type as the target dataset, albeit in a different setting. In particular, for the epileptic seizure detection task, both the base and target datasets have EEG signals, but the base dataset is collected with hospital setting devices. Similarly, in the case of AF detection, the type of signal in the base and target datasets is ECG, with the base dataset gathered in a hospital setting and the target dataset from wearable systems.

Figure 3c shows the base dataset. The base dataset is an extensive dataset, and it contains signals acquired from hospital setting devices. Crucially, the goal of MetaWearS is to minimize the data required from the scarce target dataset (Fig. 3d) by effectively leveraging this larger base dataset for pre-training; the potentially large size of the base dataset does not contradict the primary objective of reducing target data needs.

From a large pool of patients, for each episode, we randomly select non-overlapping patients for the support set and query set, respectively. We randomly choose samples from these patients to form a support set and a query set to pretrain the model in the base dataset.

Figure 3d shows the target dataset, where signals are collected from wearable devices and annotated by clinicians. The number of patients in this target dataset is significantly smaller. Here, we fine-tune the model based on this target dataset. Again, we apply the random sampling of patients to form the support set and the query set in each episode. Through fine-tuning, the model becomes adapted to wearable signals.

In the inference meta-test step, as illustrated in Fig. 3e, random patients and random samples are selected from the target dataset to create the support set and the test sample is derived from a non-overlapping patient.

### MetaWearS for reducing initial data collection

Our proposed method is inspired by the meta-transfer learning method^29^. Meta-transfer learning is a general approach in which pre-trained models on large datasets are used as a foundation for new tasks, often referred to as fine-tuning. The goal of transfer learning, in this method, is to use existing knowledge and reduce the need for extensive new training data.

Algorithm 1 formulates the explained meta-train procedure applied initially to the base dataset for pretraining and, consequently, to the target dataset for fine-tuning. During meta-train, each episode consists of a support set and a query set. The support set contains a small number *k* of samples from each class (normal and abnormal) from *N*_*S*_ patients. Similarly, the query set contains *k* samples of the same classes from *N*_*Q*_ patients. Based on prototypical networks, in our proposed method, both support and query set samples are processed by the model to extract features ${f}_{\phi }({{{\bf{x}}}}_{i})\in {{\mathbb{R}}}^{D}$ within each episode. *f*_*ϕ*_(**x**_*i*_) is considered as a *D*-dimensional vector extracted from the output of the layer that precedes the final output layer in the models.

#### Algorithm 1

A meta-train episode in MetaWearS for base and target dataset

**Input** : *f*_*ϕ*_, $${{{\mathcal{D}}}}^{{{\rm{train}}}}$$

**Output** : Updated loss ${{{\mathcal{L}}}}_{{{\mathcal{D}}}}$

**1** $${Pat}_{S}\leftarrow \,{{\mbox{Random}}}\,{{\rm{Sample}}}\,\left(\,{{\mbox{Patients}}}\,,{N}_{S}\right)$$

**2** $$Pa{t}_{Q}\leftarrow \,{{\mbox{Random}}}\,{{\rm{Sample}}}\,\left(\,{{\mbox{Patients}}}\,\backslash Pa{t}_{S},{N}_{Q}\right)$$

**3for** (*n* in {normal,  abnormal}) {

**4**$${S}^{n}\leftarrow \,{{\mbox{Random}}}\,{{\rm{Sample}}}\,\left({{{\mathcal{D}}}}_{Pat_{S}}^{\,{{\mbox{train}}}\,,{{\rm{n}}}},k\right)$$;

**5**$${Q}^{n}\leftarrow \,{{\mbox{Random}}}\,{{\rm{Sample}}}\,\left({{{\mathcal{D}}}}_{Pat_{Q}}^{\,{{\mbox{train}}}\,,{{\rm{n}}}},k\right)$$;

**6** Compute prototypes *c*_*n*_ from *S*^*n*^ in (1);

**7** }

**8for** ((*x*, *y*) in *Q*){

**9** Update classification loss ${{{\mathcal{L}}}}_{{{\mathcal{D}}}}$ by (2);

**10** Optimize *ϕ*;

**11** }

As discussed in “Conceptual introduction of MetaWearS”, a class prototype *c*_*n*_ for class *n* is defined as the average of all vectors *f*_*ϕ*_(**x**_*i*_) in the support set such that *y*_*i*_ = *n*. After computing the prototypes in the query dataset, classes with prototypes that are closer to the query feature *f*_*ϕ*_(**x**_*i*_) obtain higher probability scores. The similarity between the query sample and each class prototype is computed using squared Euclidean distance. Learning proceeds by applying softmax over the negative of these distances, and the probability for each class is obtained. The loss function ${{\mathcal{L}}}$ is computed as Equation (2).

2 $${{{\mathcal{L}}}}_{{{\mathcal{D}}}}(\phi )=\frac{1}{| {{\mathcal{D}}}| }{\sum }_{({{\bf{x}}},y)\in {{\mathcal{D}}}}l\left(\sigma (-{\left\Vert {f}_{\phi }({{\bf{x}}})-c\right\Vert }_{2}^{2}),y\right),$$

where *l* is the log-likelihood function and *σ* is softmax.

In the inference meta-test step, the prototypes are computed using Equation (1). Subsequently, the fine-tuned model, along with the prototypes, is deployed to the wearable system for patient use.

Algorithm 2 provides a detailed explanation of inference in wearable systems. When a patient uses the wearable, the acquired signal **x**_*j*_ is processed through the network, resulting in *f*_*ϕ*_(**x**_*j*_). The next step involves finding the Euclidean distance between this feature vector and the prototypes. Consequently, we can obtain the probability score for each class and classify **x**_*j*_.

#### Algorithm 2

Inference of **x**_*j*_ in the test set to tackle Challenge ①

**Input** : *f*_*ϕ*_, $${{{\mathcal{D}}}}^{{{\rm{train}}}}$$, **x**_*j*_ in $${{{\mathcal{D}}}}^{{{\rm{test}}}}$$

**Output** : Sample’s probability scores

**1** $$Pa{t}_{S}\leftarrow \,{{\mbox{Random}}}\,{{\rm{Sample}}}\,\left(\,{{\mbox{Patients}}}\,,{N}_{S}\right)$$

**2for** (*n* in {normal,  abnormal}) {

**3***S*^*n*^ ← ;

**4** Random Sample ($${{{\mathcal{D}}}}_{Pat_{S}}^{\,{{\mbox{train}}}\,,{{\rm{n}}}}$$, *k*);

**5** Compute class prototypes *c*_*n*_ from *S*^*n*^ in (1);

**6** }

**7** Compute Euclidean distances:

$${\big\Vert {f}_{\phi }({{{\bf{x}}}}_{j})-{{\bf{c}}}\big\Vert }_{2}^{2}$$;

**8** Compute the probability scores *p*(**x**_*j*_∣*y*)

### MetaWearS for updating the model using a few new shots

In this section, we discuss the methods corresponding to Challenge ②. Assume that some new annotated samples are collected from new subjects. We denote the new dataset from these subjects as ${{{\mathcal{D}}}}^{{{\rm{new}}}}$. By feeding the samples of ${{{\mathcal{D}}}}^{{{\rm{new}}}}$ data into the model, we are able to compute *f*_*ϕ*_(**x**) for all the samples. As discussed in “Conceptual introduction of MetaWearS”, we use the samples in the target set (${{{\mathcal{D}}}}^{{{\rm{train}}}}$) and the small sample set in ${{{\mathcal{D}}}}^{{{\rm{new}}}}$ to reconstruct the support set for each class *n* as follows:

3 $${S}^{n}\leftarrow \,{{\mbox{Random}}}\,\,{{\rm{Sample}}}\,\left({{{\mathcal{D}}}}_{{\rm{Pat}}_{S}}^{\,{{\mbox{train}}}\,,{{\rm{n}}}},{{\rm{k}}}\right)\cup \\ \,{{\mbox{Random}}}\,\,{{\rm{Sample}}}\,\left({{{\mathcal{D}}}}^{{{\mbox{new}}},{{\rm{n}}}},{{\rm{k}}}\right),$$

where $k\le | {{{\mathcal{D}}}}^{{{\rm{new}}}}| \ll | {{{\mathcal{D}}}}^{{{\rm{train}}}}|$. The inference algorithm in this part is identical to Algorithm 2 except Line 4, where *S*^*n*^ should be reconstructed by the support sets based on the assignment (3). For each $({{{\bf{x}}}}_{j},{y}_{j})\in {{{\mathcal{D}}}}^{{{\rm{test}}}}$, *f*_*ϕ*_(**x**_*j*_) is compared to the updated prototypes *c*_*n*_ to compute the Euclidean distance and classify the sample.

In this phase, we calculate the prototypes based on the few new shots that have been received and annotated, typically on the server side. Once these new prototypes are updated, they can be transmitted back to the wearable systems. By replacing and updating these prototypes in wearable systems, subjects can benefit from a more generalized prototype, leading to improved performance.

These updates can be performed frequently for two reasons. First, we only require a few annotated samples for the update. Second, the prototype vectors are compact enough to consume minimal energy during updates. The details of updating the prototype are discussed in detail in “Optimizing memory usage in MetaWearS based wearable systems”, where we explain Challenge ③ within the hardware system.

It is important to note that while the inference process (Algorithm 2) using the deployed prototypes runs in real-time on the wearable device (“Conceptual introduction of MetaWearS”), the update cycle involving the acquisition and annotation of new samples (${{{\mathcal{D}}}}^{new}$) typically includes an offline component^30–35^. This process depends on clinician availability, and it is performed before the updated prototypes can be computed and transmitted.

Furthermore, the computational process of updating prototypes (requiring only forward passes of new shots and embedding averaging) is significantly less complex and faster than conventional weight fine-tuning via backpropagation. Our analysis indicates that the prototype update requires ~[3x] fewer FLOPs and executes in [only 10%] of the time compared to a single fine-tuning epoch using the same data. While this computation typically occurs off-device, its low complexity further complements the primary energy savings achieved through reduced data transmission during updates.

### Hardware architecture in MetaWearS

**Fig. 4:**
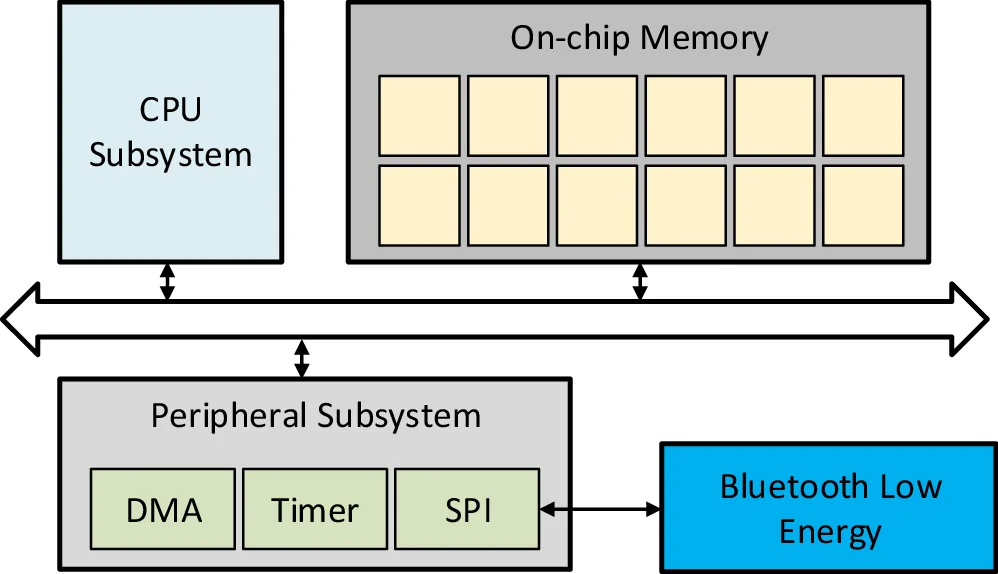
Hardware structure in X-HEEP. In the MetaWearS system, the updated prototypes are transmitted to the CPU through the BLE module.

The system considered in our scenario for wearable systems is based on X-HEEP^36^, an open-source RISC-V extensible and configurable platform for edge computing. Shown in Fig. 4, X-HEEP comprises three key hardware components: an OpenHW Group CV32E40P RISC-V processing unit (CPU)^37^, an on-chip memory, and a peripheral subsystem, all interconnected via a bus. The CPU implements RV32IMFC RISC-V extensions and is designed to deliver high performance while maintaining energy efficiency, a critical requirement for wearable devices due to their limited battery capacity.

The peripheral unit of our system encompasses several subsystems, one of which is the Serial Peripheral Interface (SPI) unit. This unit is tasked with sending and receiving data from an external wireless communication system. In our specific scenario, this unit receives all updated models and prototypes from a BLE unit. It is important to note that accessing the data via the SPI takes significantly longer than accessing the on-chip memory.

The process of updating the model or prototype, as received from the server side, unfolds as follows: The updated model weights or prototypes start arriving from BLE. The CPU then fetches these values using the SPI protocol and replaces the corresponding parameters in the on-chip memory.

In our scenario, the prototype is considerably shorter in length than the entire model’s weight. This characteristic allows us to update the prototypes with much less energy consumption, considering that, in the updating process, the bottleneck is the BLE throughput. Note that updates of all the model weights are still possible, but they become unnecessary save for large scenario changes (e.g., a change in monitoring conditions or setup).

The model is updated via a BLE-based method for the X-HEEP architecture. Updates are received by the BLE module and transmitted to the CPU via SPI protocol for updating on-chip memory. The bottleneck in this system is the BLE 4.2’s maximum data transfer rate of 1 Mbps.

## Results

### Quantitative results

**Fig. 5:**
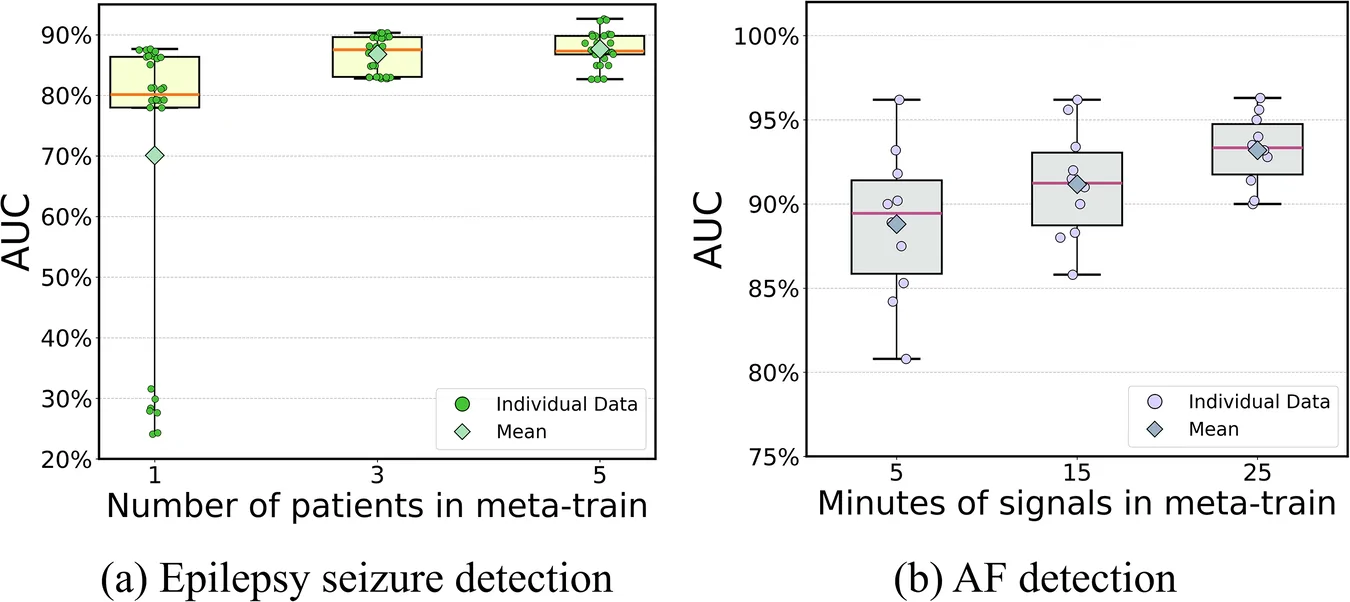
Model performance based on the amount of labeled data in the target dataset. This figure illustrates the efficacy of MetaWearS even with very limited labeled data in the target dataset, addressing challenge ①. For epilepsy *n* = 30 and AF *n* = 10.

Figure 5 shows the results of our experiments based on the amount of labeled data in the target dataset. For epilepsy, we have selected annotated groups from the target dataset with one, three, and five patients, and similarly, for AF, we have selected annotated groups with varying ECG signal durations of 5, 15, and 25 minutes to investigate the effect of the number of initial annotated signals on the performance of the model. These specific small quantities were chosen to rigorously evaluate the few-shot learning capability of MetaWearS under conditions of extreme data scarcity, representative of challenges in initial wearable system deployment, and to provide clear data points for illustrating the performance trend with minimal samples in Fig. 5.

As shown in Fig. 5, the results have improved consistently as the number of samples within each group has gradually increased.

During pretraining on the base dataset, in each episode, we set a query set size of 15 from two classes in the AF large-scale dataset and two classes in the epilepsy base dataset. At test time, we similarly sample episodes from target classes and average the results over 10 iterations to get trustworthy measures of the model performance. During fine-tuning on the target datasets, in each epoch, there are five episodes for AF and 100 episodes for epilepsy and then selected *k* = 5 samples for the support set. Also, 20% of the data is selected as validation. In case the validation loss is not decreased for five epochs, the fine-tuning is stopped.

In the meta-test phase, we perform multiple iterations to evaluate our model’s performance under varying conditions. For each iteration, the model is applied to the test data, resulting in a set of Area Under the ROC Curve (AUC). We then report the mean AUC value.

As shown in Fig. 5a, by increasing the number of patients in each group, the AUC value increases by 16%. With fine-tuning on only five patients, our model obtained an AUC value of 87.7%. Similarly, in Fig. 5b, by increasing the time of the ECG signals in each group, we have an increase in the AUC value by 2% and then 4% for 15 and 25 minutes of data in fine-tuning, respectively. Finally, with fine-tuning on 25 minutes of signals, our model’s AUC value is 93%.

#### Comparison with a source-only baseline

**Fig. 6:**
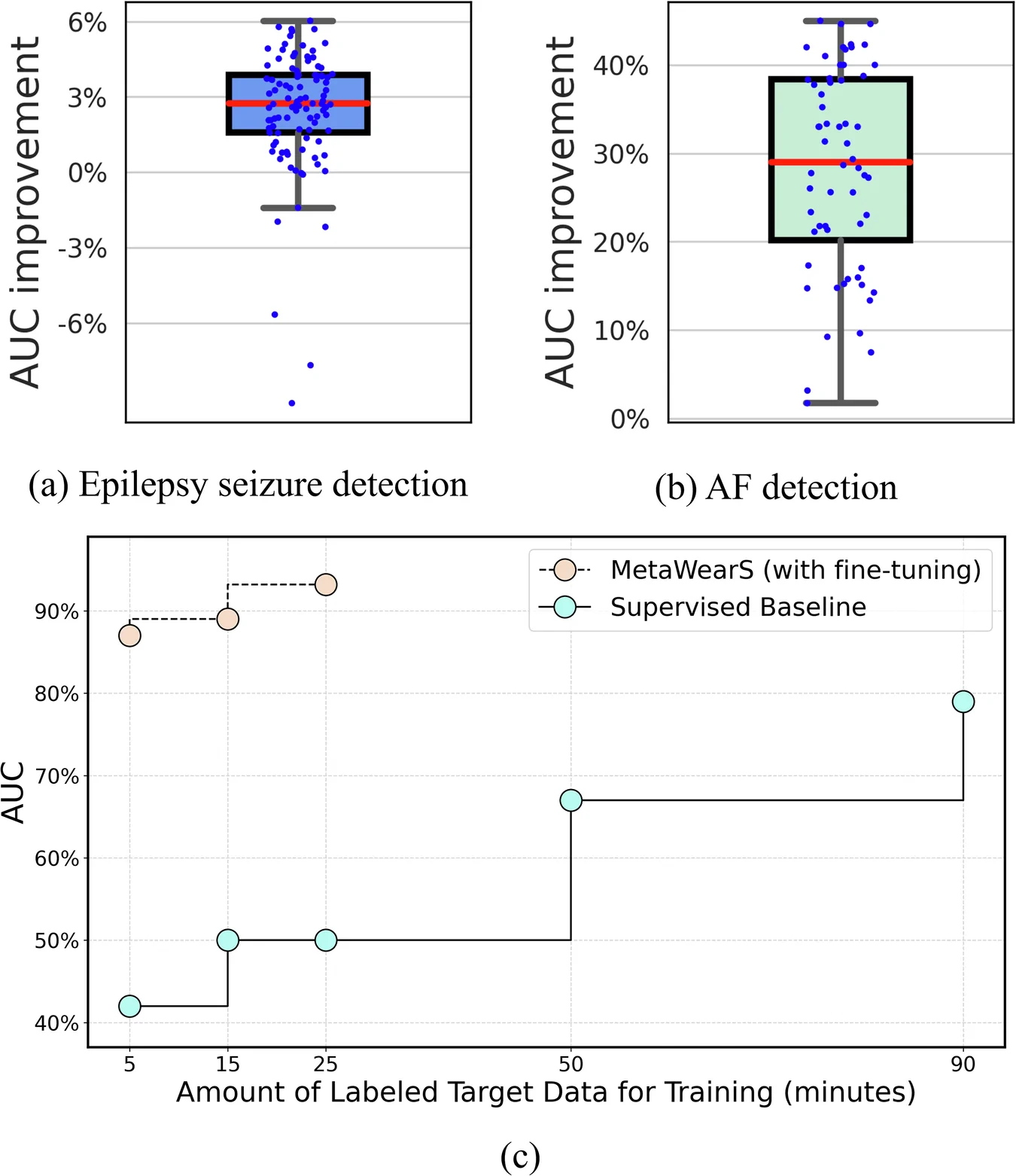
Comparative analysis of MetaWearS against source-only and target-only supervised learning baselines. **a**, **b** Box plots illustrating the distribution of AUC improvement for MetaWearS compared to the conventional source-only approach. Results are shown for **a** epileptic seizure detection and **b** AF detection across multiple experimental runs with different patient combinations from the target dataset. The analysis for seizure detection includes combinations from 14 total subjects 5, while the AF detection analysis utilizes 150 ECG samples for fine-tuning selected from the private wearable dataset. *n* = 98 and *n* = 30 experiment points are shown for (**a**, **b**), respectively. **c** Shows the effect of initial data increase in AF detection. This plot compares MetaWearS against a supervised baseline trained from scratch. The baseline performance is evaluated as the training data increases from 5 to 90 minutes of labeled signal. MetaWearS results are based on 5, 15, and 25 minutes of initial data.

To evaluate MetaWearS in comparison with a standard transfer learning strategy, we establish a Source-Only Baseline. This baseline utilizes a model with the same backbone architecture as MetaWearS, but it is trained for direct classification using a conventional classifier. This approach addresses Challenge ①-the limited data in the target dataset-by training a model on a larger base (source) dataset and then directly evaluating its performance on the target dataset without any fine-tuning or prototype-based classification. This direct transfer method is common in medical research across various data types, such as medical images^38–41^, biomedical signals^42–47^, electronic health records^48–51^, and their combinations^52–55^. This section compares the performance of MetaWearS against this Source-Only Baseline to specifically measure the performance improvement gained from our fine-tuning stage. To ensure this comparison was robust and accounted for patient-specific variability, we conducted multiple experiments using different combinations of patients from the target dataset, obtaining corresponding results for both MetaWearS and the Source-Only Baseline in each run. The AUC improvement using MetaWearS for each target dataset was then calculated by subtracting the conventional approach’s result from the MetaWearS result. Figure 6 presents the distribution of AUC improvement for various patient combinations in the target dataset for both seizure and AF detection tasks.

As shown in the figure, the median AUC improvement for MetaWearS compared to the conventional approach is 2.7% and 29.0% for seizure and AF detection, respectively. To further analyze this, a one-sample t-test was performed to determine if the AUC improvement is significantly greater than zero. The null hypothesis assumed that zero belongs to the distribution. The obtained p-value was less than 0.05, leading to the rejection of the null hypothesis. This evidence confirms that the AUC improvement is statistically greater than zero, indicating that the MetaWearS model outperforms the conventional approach in the studied detection tasks.

The results reveal a greater performance difference between MetaWearS and the conventional approach for AF detection compared to seizure detection. In particular, the AF detection task does not exhibit instances of extreme under-performance or outliers. This observation can be attributed to the larger discrepancy in signal features between the target and base datasets for AF detection. This is because the AF task utilizes data from a wearable device in the target dataset, whereas seizure detection utilizes data from a hospital setting, different from the base hospital data. This issue is further discussed in “Discussion”.

#### Comparison with a target-only supervised baseline

To explicitly quantify the benefit of MetaWearS in reducing the need for target-specific labeled data (Challenge ①), we compared its performance against a Target-Only Supervised Baseline. This baseline model was trained from scratch using only the labeled data from the target dataset. We incrementally increased the amount of training data available to the baseline, from a few minutes up to 90 minutes of signal, to observe the performance trajectory. We first attempted to use a model with the same architecture as MetaWearS for the baseline; however, due to the severe scarcity of initial data, this model showed significant overfitting and failed to achieve a satisfactory performance level. To facilitate a more meaningful comparison, we then employed a more compact MobileNet-style architecture with fewer parameters for the baseline, as used in ref. ^27^, which is better suited for training on smaller datasets. The test set was kept consistent across all experiments to ensure a fair evaluation.

The results for the AF detection task are presented in Fig. 6c. The figure plots AUC against the minutes of labeled signal used for training. As illustrated, MetaWearS achieves a high performance of 89% AUC using only 5 minutes of the labeled ECG signal from the target user. In stark contrast, the supervised baseline requires significantly more data to even begin learning effectively. It only starts to show a significant improvement after 50 minutes of signal, reaching an AUC of 68.2%. Even when provided 90 minutes of training data, the baseline performance remains below the 80% AUC, failing to reach the level achieved by MetaWearS with a fraction of the data.

This result clearly quantifies the data efficiency of our approach. MetaWearS can achieve high accuracy with a lower amount of initial data, whereas a traditional approach would require a substantially larger, and in this case prohibitively large, dataset to approach comparable performance. This directly validates our claim that MetaWearS effectively addresses the challenge of reducing the initial data burden for personalizing wearable sensor models.

### Updating the model using a few new shots

In the second phase of wearable system lifecycles, the wearable devices equipped with our model are distributed to new patients for detection purposes. The wearable systems begin collecting signals from the individuals. The system is designed to detect target abnormalities and transmit this data to a server for further processing. As data is accumulated from the wearable systems, clinicians are able to annotate the signals. In this way, a new labeled sample is available for updating the model. In this section, we discuss the reduction of the number of new labeled data for updating the model using MetaWearS to address Challenge ②.

As shown in Fig. 3f, the new shot is included in the support set that was also created with samples from the target dataset. Therefore, the support set is updated, and the new few shots play a crucial role in the inference step.

To evaluate the effectiveness of our model update scenario for the detection of AF and epilepsy, we conducted experiments to evaluate the results of the phase where prototypes are calculated based on the newly received annotated shots, typically on the server side. For AF, we performed fine-tuning on the group that included the smallest number of patients, which is equal to 30 ECG records and takes 5 minutes, and then we only took a few shots from other groups of patients that are not part of the test data in the meta-test phase. For epilepsy, fine-tuning was performed in one patient, and then new shots were taken from one and two additional patients. These experiments involved varying the shot values of *k* = 1, 3, 5, 10, 15, and 20.

**Fig. 7:**
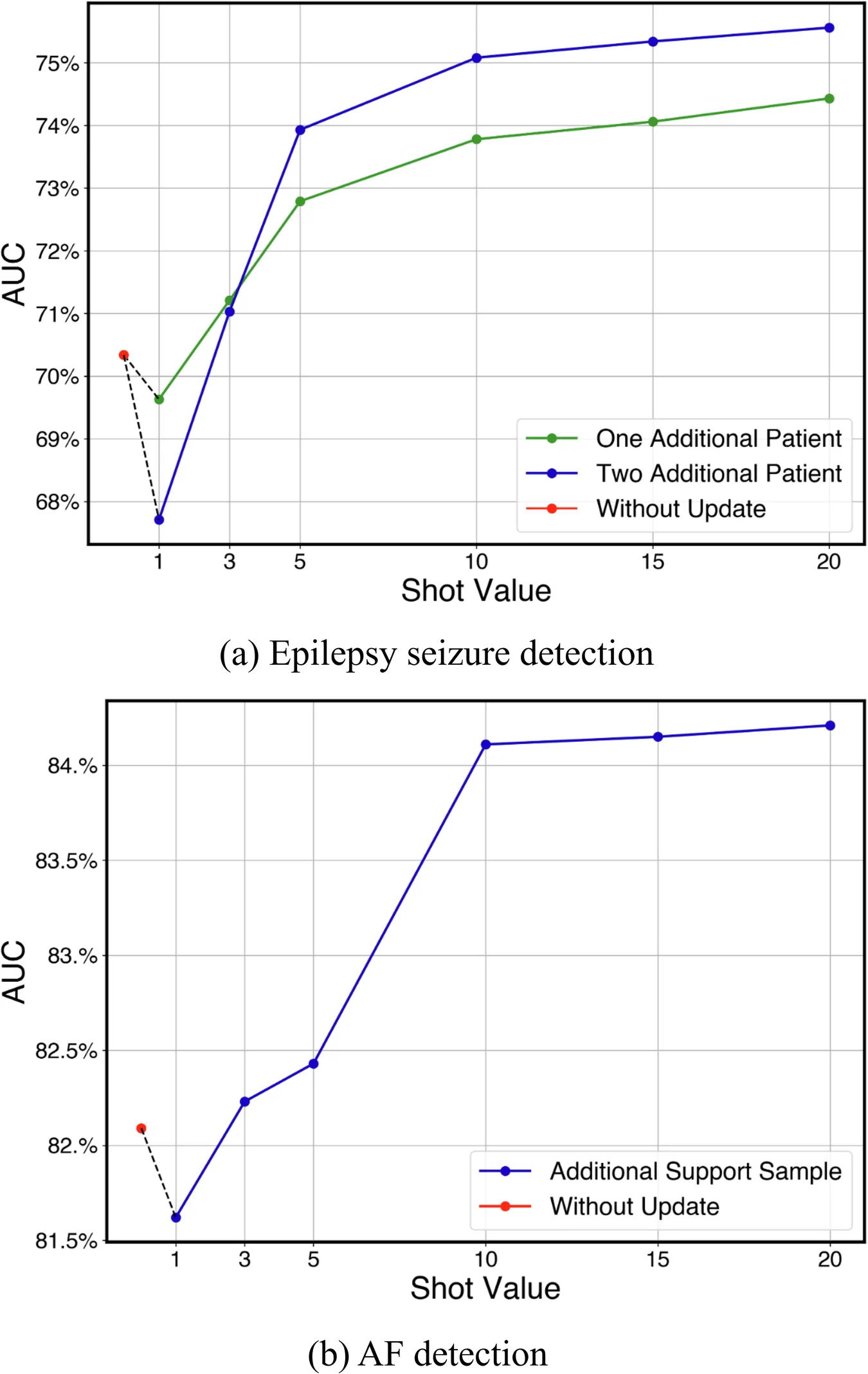
Improving model performance using a few new shots to address challenge ②. In both cases, AUC is increased by updating prototypes when *k* ≥ 3 new shots are received. For epilepsy (**a**), updates are tested using *k* = 1, 3, 5, 10, 15, and 20 shots from one and two additional patients. For AF detection (**b**), updates are tested with the same *k* values using additional ECG records from the wearable dataset.

As shown in Fig. 7a, for epileptic seizure detection, the baseline AUC is 70.3%, where no additional shots are applied. When *k* = 10 shots are received from a single additional patient in ${{{\mathcal{D}}}}^{{{\rm{new}}}}$, there is an improvement in the AUC by 3.4% compared to the baseline. This improvement is even more pronounced when *k* = 20 shots are received from the same patient, resulting in 74.4% of AUC.

The figure also presents the results when two additional patients shared their shots. With *k* = 20 new shots from these two patients, the model can achieve 75.6% of AUC, which is 5.3% more than the baseline, only by a total of 16 minutes of signals.

An observation in Fig. 7 in both cases of epileptic seizure detection and AF detection is the slight performance degradation compared to the baseline when only a single shot is received from one or two additional patients. The reason for the performance degradation can be the low robustness of randomly selecting a single shot as opposed to multiple shots. This phenomenon is explained in more detail in “Discussion”.

### Battery life with MetaWearS

Addressing Challenge ③ that considers the energy efficiency of the updates, we use X-HEEP as the energy-efficient and low-power system for implementing the models^36^.

The X-HEEP hardware supports both low-latency and low-power modes, detailed in Tables 1 and 2. In low-latency mode, the system uses a 1.2 V supply and 450 MHz frequency, allowing rapid execution and subsequent idling. Table 1 shows the results for the epileptic seizure detection task. The transformer model used in this task processes each 12-second window as input. Remarkably, we observe that the processing time for each window is 1.9 seconds when X-HEEP operates at the low-latency mode. Also, in AF detection, we process a 10-second window in 0.76 seconds at the low-latency mode. The estimated power consumption is 50 mW during operations. Using a battery with a capacity of 480 mAh, we can execute the seizure detection and AF detection tasks on X-HEEP for 24.3 and 27.3 hours, respectively. Therefore, thanks to the optimized implementation and quantization efforts undertaken for these models, they can operate in real-time on the X-HEEP system.

**Table 1:** Epilepsy detection performance comparison using a 12-second EEG signal window across various hardware platforms

Platform	Mode	Frequency	Active power	Idle power	Voltage	Exec. time	Battery life
		[MHz]	[mW]	[mW]	[V]	[seconds]	[hours]
X-HEEP	Low-latency	450	50	18.7	1.2	1.9	24.3
	Low-power	75	3.7	–	0.8	12	103.8
STM32 Nucleo	Low-latency	120	132	77.5	1.8	1.673	10.2
	Low-power	16	35.5	27.9	1.8	11.62	24.5
RPi Pico	Low-latency	133	82.3	23.7	3.3	3.752	37.7
	Low-power	41	38.7	18.8	3.3	12	40.9

Conversely, the low-power mode focuses on reducing power usage by adjusting frequencies to just meet real-time demands, eliminating idle periods. Here, epilepsy detection operates at 75 MHz with 3.7 mW power, and AF detection at 34.4 MHz with 1.9 mW power. A battery with the same capacity of 480 mAh can last in the low-power mode as long as 103.8 and 202.1 hours for seizure detection and AF detection, respectively.

For epilepsy detection, updating the entire model via the bluetooth low energy (BLE) module takes 239 milliseconds, which is 12% of the total execution time. For AF detection, updates take 1.7 seconds, amounting to 225% of the execution time, significantly impacting performance.

A common approach to updating models on wearable devices involves sending the entire updated model back to users’ devices from the server. This method is prevalent in federated learning scenarios, as seen in several works^56–61^.

In contrast, our proposed MetaWearS method enables us to update the wearable system by only updating the prototypes instead of updating the entire model’s weights. This benefit reduces the time and energy consumption for updating the framework by 456x and 418x for epileptic seizure detection and AF detection, respectively. In case of frequent updating of the model, the battery life is decreased significantly in the conventional approach.

#### Hardware platform trade-offs

To provide a comprehensive evaluation of MetaWearS’s performance and applicability, we extended our analysis beyond the custom RISC-V-based X-HEEP platform. We deployed and benchmarked our system on two widely-used, commodity hardware platforms: the STM32 Nucleo-L4R5ZI-P, which features an ARM Cortex-M4 core, and the Raspberry Pi Pico, built on a dual-core ARM Cortex-M0+. These platforms represent standard choices for embedded and wearable device development, allowing for a direct comparison between a specialized, energy-centric RISC-V implementation and mainstream ARM architectures.

For both the epilepsy and AF detection case studies, we measured three key metrics: inference latency (execution time), power consumption (active and idle), and estimated battery life. To ensure a fair comparison, battery life for all platforms was calculated based on a standard 480 mAh battery.

The complete performance results for the epilepsy detection task are detailed in Table 1. The analysis reveals a clear trade-off between latency and energy efficiency. In their respective low-latency modes, the STM32 Nucleo achieves the fastest inference time at 1.67 seconds, closely followed by the X-HEEP at 1.9 seconds. However, in terms of energy efficiency, the X-HEEP platform is the undisputed leader. Its low-power mode yields an estimated battery life of 103.8 hours, which is over four times longer than the STM32’s 24.5 hours and more than double the Raspberry Pi Pico’s 40.9 hours.

For the AF detection application, as shown in Table 2, the X-HEEP platform demonstrates superior performance across both latency and efficiency. In its low-latency mode, it completes an inference in just 0.76 seconds-nearly 6 × faster than both the STM32 Nucleo (4.41 s) and the Raspberry Pi Pico (4.53 s). This efficiency advantage is even more pronounced in the low-power mode, where the X-HEEP achieves an estimated battery life of 202.1 hours. This is approximately 6 × longer than the baselines, which both offer ~33 hours. This remarkable efficiency is primarily due to X-HEEP’s extremely low active power draw of only 1.9 mW in this configuration.

**Table 2:** AF detection performance comparison using a 10-second ECG signal window across various hardware platforms

Platform	Mode	Frequency	Active power	Idle power	Voltage	Exec. time	Battery life
		[MHz]	[mW]	[mW]	[V]	[seconds]	[hours]
X-HEEP	Low-latency	450	50	18.7	1.2	0.76	27.3
	Low-power	34.5	1.9	–	0.8	10	202.1
STM32 Nucleo	Low-latency	120	124	77.5	1.8	4.41	8.8
	Low-power	45	62	44.5	1.8	9.9	14.0
RPi Pico	Low-latency	133	80.4	23.7	3.3	4.527	32.1
	Low-power	60	47	19.8	3.3	10	33.7

These results highlight the distinct advantages of each architectural approach. The X-HEEP platform, with its configurable RISC-V core and specialized features like its ultra-low-voltage (0.8 V) operating point, is explicitly optimized for energy-efficient, long-duration monitoring tasks. In contrast, the ARM-based STM32 and Raspberry Pi Pico provide highly accessible, general-purpose development ecosystems. The choice of hardware is therefore dictated by the specific requirements of the target application: for applications demanding the lowest possible latency, the STM32 Cortex-M4 proves competitive, whereas for those prioritizing maximum battery life, the specialized RISC-V architecture of X-HEEP is unparalleled.

Crucially, this expanded evaluation demonstrates that while the underlying hardware platform influences execution time and power draw, the core principle of MetaWearS-updating a compact prototype to avoid transmitting large raw data or full models-delivers substantial energy savings universally. The 418 × to 456 × reduction in energy consumption from minimized data transmission is a platform-independent benefit that underscores the robustness of our approach.

#### Optimizing memory usage in MetaWearS-based wearable systems

Supplementary Table 1 provides the details of the models used in this study. In the case of epilepsy, the transformer model is deployed on X-HEEP using 16-bit fixed-point parameters. Within the transformer, the linear operations are executed with fixed-point operations, while the non-linear operations, namely Softmax, Normalization, and GELU activation, are operated by a 32-bit floating-point unit. While quantization of the parameters and the linear operations to 16-bit enables us to save data size and increase performance, it has a negligible effect on the results, with only a 0.1% drop in AUC in the case of epileptic seizure detection using the proposed method. In particular, the quantized implementation fits entirely in the on-chip memory, which leads to higher energy efficiency because no data movements from the external memory are needed. In addition, quantization to 16-bit integer numbers allows for higher performance on the CPU with respect to floating-point operations. For the AF detection task, the MobileNetV2 model operates with 32-bit floating-points both for the parameters and operations. Based on these parameters and operation types, in Supplementary Table 1, we show the sizes of the models, input, intermediate output, and prototypes for these two case studies.

The synthesized system features 384 KB of on-chip memory divided into 12 memory banks, storing the input signal, the model’s weight, the prototypes, and the intermediate output. The intermediate output is generated during the inference process, where the model saves the output of each layer for further processing. The part dedicated to the intermediate output can be overwritten during the computation. This memory optimization strategy is particularly beneficial in wearable systems where memory resources are limited.

We emphasize that while quantization is crucial for efficient deployment on resource-constrained hardware like X-HEEP, the quantization techniques themselves are standard practices. The core innovation of our work lies in the MetaWearS framework for few-shot learning and efficient prototype-based updates within the wearable systems lifecycle.

## Discussion

In this work, we have discussed several significant obstacles that are currently at the cutting edge of personalized healthcare and wearable technology. In Challenge ①, we highlight the issue of insufficient data collected from wearable devices, which often reduces the effectiveness of machine learning models. Further research has been conducted to advance meta-learning strategies that address these challenges in various health monitoring applications^62^. One study introduces MetaPhys, a meta-learning framework that optimizes remote physiological measurements and is capable of high-speed inference, showing promise for edge or wearable devices^63^. Furthermore, a study explores the use of model-agnostic meta-learning (MAML)^64^ to improve wearable platforms for real-time glucose monitoring, specifically in the management of type 1 diabetes^65,66^. The study in ref. ^67^ presents a meta-learning neural network framework for genomic survival analysis, achieving effective predictive modeling with limited data and highlighting key cancer-related genes and pathways. Other works^68,69^ introduce models, which effectively generalize across multiple datasets using limited mobile sensing data.

While these papers tackled the challenge of limited data and cross-dataset through meta-learning, they have not taken into account the scenarios where the training set has no data from the target subject. In the context of wearable devices, acquiring annotated data from each user for model training is not only costly but also time-intensive due to the infrequency of abnormal events. In MetaWearS, we have clearly separated the meta-train and meta-test based on individual patients to have the real-world applicability of our proposed algorithm. Furthermore, while the mentioned papers provide foundational insights, they have not considered hardware implementation in their methods, nor have they addressed the ongoing updates to the framework over time, which we identify as Challenge ②.

In Challenge ②, we introduced MetaWearS to update models efficiently with minimal wearable device data. Unlike the other update processes performed in the previous study, such as integrating multi-resolution kernels^70^ or optimizing memory management through gradient descent^71^, MetaWearS simplifies the update process. Traditional approaches, like the hardware-software integration for energy efficiency^72^ or using the modified hardware-aware meta-learning process^73^, often increase computational demands and energy consumption. Some recent researches focus on self-powered flexible devices using energy harvesting and low-power designs optimized for healthcare applications like real-time health monitoring^74–76^. The main issue with these methods is their requirement for a stable supply voltage, which is incompatible with the fluctuating output of miniature energy harvesters. In contrast, MetaWearS minimizes both, reducing the execution time and resource use critical in time-sensitive and energy-constrained wearable environments. Furthermore, the need for a lightweight and energy-efficient model suitable for deployment in wearable devices was important, a challenge that we defined as Challenge ③, which is inefficiency in frequent updates of deep learning models on wearable devices. The studies in refs. ^27,77,78^ tackled the model deployment on wearable devices by customizing solutions specifically for the resource constraints of these platforms. Also other previous works, federated learning has been employed to update a global model via the Federated Averaging algorithm, which involves centralized aggregation of local data adjustments. This process continues with iterative local and global optimizations, including the exchange of model parameters between the central server and local nodes^56^. In addition, another study introduces Relay Learning, in which models are updated and passed from one clinical site to another sequentially. This process is facilitated by a central server and enhances data privacy by maintaining physical disconnection during model transmission^59^.

While previous studies have offered energy-efficient real-time solutions for detecting biomedical abnormalities, our MetaWearS proposal takes a more comprehensive approach. Not only does it consider the energy efficiency of the wearable system, it also addresses the entire lifecycle of such systems. We have addressed the challenges of limited data in wearable training sets and updated the model with only a few data points. Moreover, our method has been extended to cover two distinct domains, heart and brain signal abnormality detection. Our experiments focus on two binary detection tasks, epileptic seizure detection and AF detection. Future work may explore whether the observed generalization behavior extends to multi-class or more diverse clinical settings.

While the main results demonstrate the effectiveness of the proposed method, it is important to evaluate the performance of MetaWearS by comparing it with established few-shot learning methods. This comparison shows that the improved lifecycle efficiency of our approach does not reduce classification performance. To contextualize the performance of MetaWearS, we benchmarked it against several established few-shot learning algorithms, including MAML^64^, Matching Networks^79^, and Relation Networks^80^. Our analysis confirms that MetaWearS provides its significant lifecycle efficiency benefits without compromising on classification performance.

When comparing MetaWearS to established methods like MAML, the primary distinction lies in the update mechanism, which directly impacts the wearable lifecycle (Challenges ② & ③). MAML adapts by fine-tuning its model weights via gradient descent, requiring the wireless transmission of the entire updated model for on-device deployment. In stark contrast, MetaWearS leverages its Prototypical Network foundation to perform updates by simply recalculating and transmitting a single, compact prototype vector. This architectural difference leads to an orders-of-magnitude reduction (418–456x) in the energy required for model updates.

Having established this efficiency advantage, for the performance comparison, we followed a methodology similar to the one used in “Data preprocessing and network architecture”. All methods were first pre-trained on their respective base datasets (TUSZ EEG, PhysioNet ECG) using their standard meta-training procedures. Subsequently, we evaluated their few-shot classification performance on the corresponding target datasets (Siena EEG, private wearable ECG).

Our experiments confirm the MetaWearS advantage on update efficiency does not come at the cost of performance. For the seizure detection task, the classification performance of MetaWearS was not statistically different from MAML (*p* > 0.05), and MetaWearS has a mean AUC improvement of 0.9%. For the more challenging AF detection task, which involves a greater domain shift, MetaWearS demonstrated a statistically significant improvement over MAML, with an average AUC improvement of 6.4% (*p* < 0.05). This performance gap can be attributed to MAML’s known vulnerability to overfitting on small support sets, whereas the implicit regularization of our prototype-based approach ensures greater robustness.

This fundamental advantage in lifecycle efficiency extends to other prominent metric-based approaches, namely Matching Networks and Relation Networks. These architectures share a critical limitation: unlike MetaWearS, which condenses all support set information into a single prototype, they require the feature vectors of every individual sample in the support set for classification. To update the system with *k* new shots, all *k* feature vectors must be transmitted, a process that is *k* times less efficient than the single prototype update of MetaWearS.

While being architecturally more efficient, MetaWearS also demonstrated competitive accuracy. In the case of AF detection, it significantly outperformed Matching Networks with a mean AUC improvement of 2.8% (*p*  < 0.05) and achieved an accuracy similar to the highly competitive Relation Network, with a mean improvement of 1.7% (*p* > 0.05). This unique combination of validated high performance and an architecturally superior, energy-efficient update mechanism solidifies the value of the MetaWearS framework for practical wearable systems.

Beyond these quantitative benchmarks, a qualitative examination of how the model represents information provides deeper insight into its learning process. By using dimensionality reduction techniques to visualize support and query data, we can observe the impact of fine-tuning and the shift of prototypes as they adapt to new users.

**Fig. 8:**
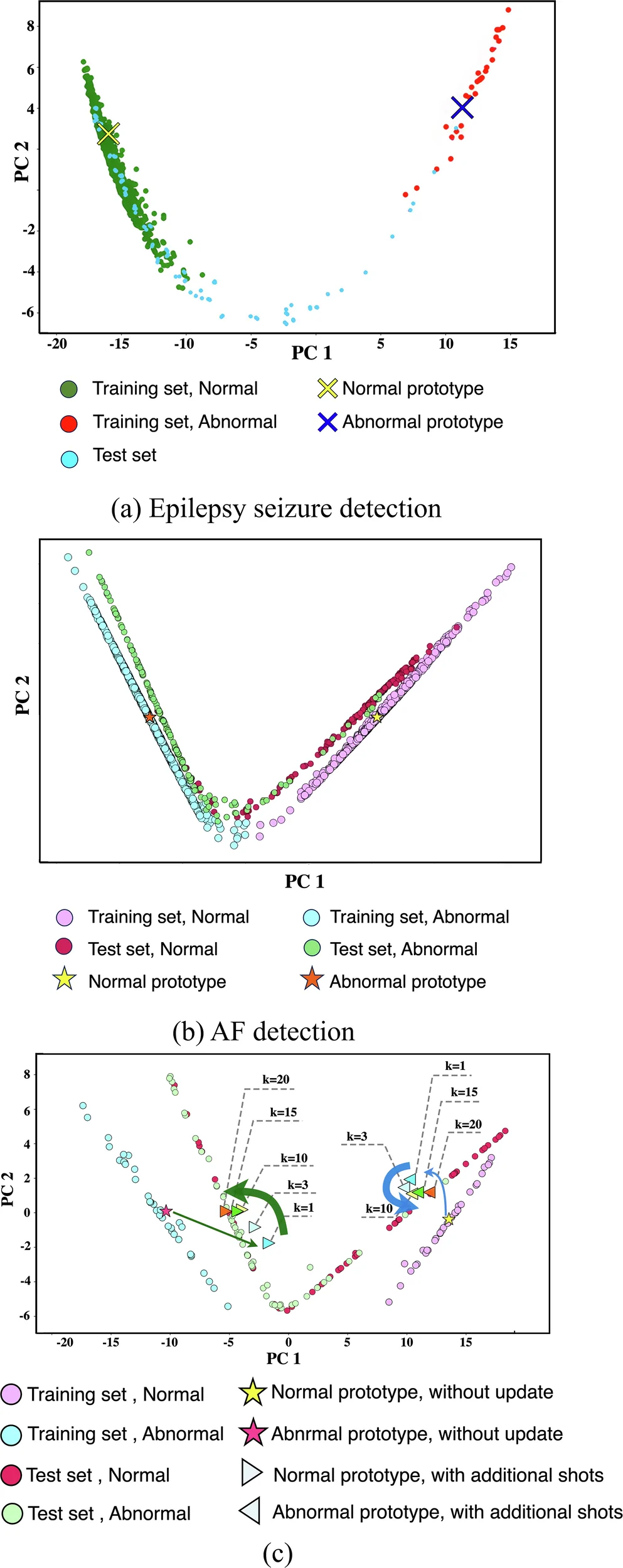
Evaluation of feature space separability and prototype trajectories during updates. **a**, **b** PCA analysis of support and query data after fine-tuning for **a** epilepsy and **b** AF, demonstrating enhanced separability and clustering of the datasets. **c** Prototype updating and their trajectory based on the number of new shots to address Challenge ②. As more new shots are involved, the prototypes progressively align closer with the training and test data, effectively fine-tuning the model. The arrows show the movement of prototypes from their initial positions (marked by stars) toward better generalization with the test data.

In Fig. 8a and b, we present a visual representation of the support and query data, highlighting the impact of fine-tuning in our meta-learning framework in Challenge ①. Figure 8a presents the support and query data related to epilepsy patients, which has been subject to dimensionality reduction through Principal Component Analysis (PCA). In Fig. 8b, we provide a parallel analysis for AF, with both cases being processed similarly using PCA for feature space transformation. This visualization showcases the distribution of query data points after fine-tuning. In particular, our results reveal distinct clustering patterns between the support and query data after fine-tuning, indicating a substantial improvement in their separability.

Figure 8a, b, presents PCA visualizations of the feature embeddings for support and query (test) data after fine-tuning. While the 2D projection may not fully capture the separability in the original high-dimensional space, particularly for the dispersed AF test samples, the visualization illustrates the relative positioning of support data clusters and class prototypes after fine-tuning. The effectiveness of the learned feature space in separating the classes is quantitatively demonstrated by the high AUC performance achieved on the test set (see “Methods”).

Furthermore, we show the results corresponding to Challenge ②, where the data requirement for the process to update the values poses a significant consideration. To gain a better understanding of this phenomenon, we visualized the feature set of labeled samples in the target dataset and the samples in the test set in a two-dimensional representation using PCA. Figure 8c shows this representation for AF detection. The prototypes of the model, before receiving any update, are denoted by stars for each class. The arrows show the trajectory of the prototypes as they receive new shots from additional samples.

As seen in Fig. 8c, when a single shot is received, the updated prototypes slightly deviate from the train and test samples. This deviation sometimes results in a slight performance decrease compared to the baseline (no update), as observed in Fig. 7. This result is not an anomaly but a known challenge in Incremental Few-Shot Learning^81–83^, often described as a form of catastrophic forgetting^84,85^. The core of the issue lies in the statistical properties of the update itself. A class prototype is an estimator of the true class centroid, and a single sample provides a high-variance, statistically unstable estimate. If this single sample is an outlier or atypical, it can corrupt the prototype’s position, disrupting the learned geometry of the entire metric space and degrading overall performance. This observation aligns with previous studies^86,87^ showing that naive adaptation with extremely scarce data can be more detrimental than no adaptation at all.

In contrast, as more shots are received and the prototypes are updated with these shots, the prototypes align more closely with the test sample line. This result shows that in MetaWearS a k-shot update (where *k*≥3) leverages the power of averaging to provide a more robust, lower-variance estimate of the class centroid, leading to a more stable and constructive adaptation that improves generalization.

The practical utility of such visualizations must be considered alongside the model’s robustness to common environmental challenges encountered in real-world wearable monitoring. We therefore evaluated the framework’s robustness against simulated noise and its inherent scalability for more complex clinical settings.

To assess the framework’s resilience against common artifacts in non-controlled environments, we evaluated its performance on target test sets corrupted with simulated white noise. After standard preprocessing, noise was added at varying signal-to-noise ratios (SNRs). For the epilepsy detection task, where the model was fine-tuned on five patients (with a baseline AUC of 87.7%), the absolute AUC loss (Supplementary Fig. 1a) was over 10% at a low 5 dB SNR. However, the model demonstrated strong resilience, with the loss decreasing to only 2.5–3% at a moderate 25 dB SNR, preserving a robust AUC of approximately 85.2–85.0%. Degradation became negligible at 40 dB SNR. Similarly, for AF detection, where the model was fine-tuned on 25 minutes of data (with a baseline AUC of 93.0%) and ECG signals were affected by motion artifacts and baseline wander, the absolute AUC loss (Supplementary Fig. 1b) was approximately 30% at 5 dB SNR, reflecting significant interference. Yet, performance notably improved with higher SNRs, reducing to 17–17.5% at 25 dB SNR, corresponding to an AUC of ~75.5–76.0%. At 40 dB SNR, the loss was a minimal 2.5–3%. While performance degrades with increasing noise, these results indicate that MetaWearS maintains reasonable efficacy under common noise conditions, particularly when coupled with appropriate signal preprocessing.

A notable strength of MetaWearS’s episodic training design is its inherent robustness to class imbalance during the fine-tuning and update phases on the target device. Because we explicitly sample a small, fixed number of shots (*k*) per class for both support and query sets in each episode, the learning process itself is balanced on a per-episode basis. This mitigates concerns about the model being skewed by disproportionate class representation within the target few-shot data. The primary challenge related to imbalance in the target domain then becomes the practical one of sourcing at least *k* representative samples for each class, especially rare ones. Imbalance within the base dataset, which is used for pre-training, is a more traditional concern; for instance, we addressed this in the base AF dataset through random resampling and Gaussian noise augmentation to ensure the pre-trained model is not unduly biased before adaptation. Thus, MetaWearS’s structure inherently manages imbalance at the crucial few-shot adaptation stage, provided a minimal number of representative samples per class can be obtained.

MetaWearS is also inherently scalable to non-binary classification tasks, as Prototypical Networks compute a distinct prototype for each class. Our epileptic seizure case study already demonstrates its use with multi-channel EEG data. Extension to multi-modal signals would primarily necessitate the design of a suitable feature extractor *f*_*ϕ*_ capable of processing and fusing diverse data streams into a coherent embedding space, from which prototypes can then be derived. While this might increase the complexity of *f*_*ϕ*_, the core meta-learning and efficient prototype-update mechanisms of MetaWearS remain applicable, offering a scalable approach for various complex wearable sensing scenarios.

In order to investigate the effect of the base dataset, we conducted experiments without pretraining the model on the base dataset. According to the results shown in Supplementary Fig. 1a and b for both AF and Epilepsy, we observed that the model’s performance is significantly affected (p-value  < 0.05) by the absence of a base dataset. In particular, as shown in Supplementary Fig. 1c for epilepsy, the AUC values exhibited an 8% decrease without the use of the base dataset. In Supplementary Fig. 1d, for AF, AUC is decreased by up to 7%, demonstrating the advantages of pretraining the model on a base dataset in MetaWearS to address Challenge ①.

Further ablation study explores the effect of directly testing the target dataset without fine-tuning. In this configuration, we removed the fine-tuning step from the meta-training. In Supplementary Fig. 1c and d, we compared the results of this experiment with the results of our proposed strategy. For AF, the mean AUC shows a significant 10% decrease (*p* value < 0.05), underscoring the critical role of fine-tuning in enhancing performance. Meanwhile, in the case of epilepsy, we observed a more pronounced effect, with a significant decrease in AUC of 12.5% (*p* value  < 0.05).

Finally, while these results demonstrate a significant advancement in managing the lifecycle of wearable models, it is important to acknowledge the specific constraints of the current study. Our work presents the following key limitations. Firstly, we trained MetaWearS using publicly available datasets (TUH for seizure detection^20^ and PhysioNet for AF detection^26^) where data originated from hospital settings. However, the scarcity of public datasets involving wearable devices and data collected outside hospitals hinders a more generalizable evaluation. To partially address this, we employed the Siena dataset^23^, albeit small and still hospital-based, to simulate data from a different clinical setting. For AF detection, we were able to leverage a private dataset with wearable device data. The availability of publicly accessible wearable device datasets in the future holds promise for a more comprehensive evaluation of the effectiveness of our framework.

Secondly, the limited size of the target wearable device datasets restricted our ability to analyze the impact of seizure/AF types or the patients’ age on our framework’s performance. We believe that future research with larger datasets that separate patient data by AF/seizure types or ages would enable better fine-tuning in Challenge ① and targeted model updates focused on specific types of abnormalities in Challenge ②.

Furthermore, the efficacy of MetaWearS, like other approaches relying on learned embeddings, is inherently tied to the quality of the feature space generated by the encoder *f*_*ϕ*_. A poorly discriminative feature space will inevitably limit the performance of the prototype-based classifier.

Finally, a key consideration in wearable health monitoring is the model’s ability to generalize to new, unseen patients, particularly given the high inter-patient variability common in biomedical signals like EEG. MetaWearS is designed to inherently address this through its meta-learning paradigm. The episodic training during both pre-training and fine-tuning (“MetaWearS for reducing initial data collection”) explicitly trains the model to make predictions for patients not seen in the support set of a given episode. Furthermore, the fine-tuning process itself adapts the model to the target population, and the prototype update mechanism (“Ethical approval and data sources”) allows for ongoing personalization as data from new users becomes available. While these strategies aim to enhance generalization, the extent of domain shift between the base data, initial target data, and new user data remains a factor influencing performance. Future work could explore the robustness against severe data variability and improve calibration for highly diverse patient cohorts.

## Conclusion

In this paper, we have proposed a novel few-shot learning method to detect abnormalities in scarce signals acquired by wearable systems. In the first phase of the wearable device’s lifecycle, we have significantly reduced the amount of data collection for the initial training of the network. We have shown that by using only three subjects in the epileptic seizure detection use case, we can fine-tune the model and obtain 86.8% of AUC. This performance is 82% for AF detection with only 5 minutes of data. In the second phase of the wearable’s lifecycle, where the wearable systems are being used by the end-users, we have also reduced the required labeled data for updating the framework using MetaWearS. We have shown that with only 16 minutes of additional labeled signals, we can improve AUC to 75.6% in the epilepsy task. Furthermore, we have measured the time and power consumption for the transformer model for a 12-second signal window as 1.9 seconds and 50 mW, respectively. The update process, due to MetaWearS, can consume up to 456 times less than updating the entire model.

## Supplementary information

Transparent Peer Review file

Supplementary material

## Data Availability

The datasets and source data underlying the findings of this study are available as follows: Temple University Hospital EEG Seizure Corpus (TUSZ) v2.0.0: the dataset used for epilepsy pre-training is publicly available via the Neural Engineering Data Consortium at Temple University at https://isip.piconepress.com/projects/tuh_eeg/. Siena Scalp EEG Database: the dataset used for epilepsy target evaluation is publicly available through PhysioNet at https://physionet.org/content/siena-scalp-eeg/ (10.13026/5d4a-j060). PhysioNet Computing in Cardiology Challenge 2017: the ECG dataset used for atrial fibrillation (AF) pre-training is publicly available through PhysioNet at https://physionet.org/content/challenge-2017/ (10.22489/CinC.2017.065-469). Source Data: numerical results underlying all graphs and charts are provided as CSV files in the project’s GitHub repository (https://github.com/esl-epfl/metawears) and are archived on Zenodo^88^ at 10.5281/zenodo.18262590.

## Code availability

The code for the experiments is available in the following repository: https://github.com/esl-epfl/metawears.
