# Supplementary material for: A meta-learning method with reduced data requirements for training and updating deep learning models on wearable devices
Source: Commun Med (Lond). 2026 Jul 31;6:523. doi: 10.1038/s43856-026-01740-1 (PMC13650112; doi:10.1038/s43856-026-01740-1)
Supplement: Supplementary file 1 [file 43856_2026_1740_MOESM1_ESM.pdf]

nature portfolio

Peer Review File

A meta-learning method with reduced data requirements for training and updating deep learning models on wearable devices

Corresponding Author: Professor Siamak Mohammadi

This file contains all reviewer reports in order by version, followed by all author rebuttals in order by version.

Version 0:

Reviewer comments:

Reviewer #1

(Remarks to the Author)
Summary of the paper: The paper introduces MetaWearS, a meta-learning framework designed to address the challenges of data scarcity and resource efficiency in wearable systems. The authors propose a method that requires minimal initial data collection and an efficient updating mechanism that enhances the lifecycle of wearable devices. Through case studies on epilepsy and atrial fibrillation detection, the paper demonstrates the efficacy of MetaWearS in reducing data requirements while maintaining high performance

Strength of the paper:
Reproducibility: The paper provides a thorough description of the experimental setup, including hardware details, experiment hyperparameters, and data preprocessing procedures. This enhances the reproducibility of the study, which is helpful for validating the proposed method, even though part of the data remains private.
Clear Organization: The paper is well-organized, with a logical flow that makes it easy to follow the authors' arguments and experimental results. This clarity supports a better understanding of the complex methodologies employed.
Energy Efficiency: One of the significant contributions is the reduction in energy consumption during model updates, which is critical for the deployment of deep learning models on resource-constrained wearable devices. The paper demonstrates a substantial reduction in power usage, which is a strong point for practical applications.

Weakness of the paper:
Experimental Focus: If the primary claim of MetaWearS in addressing challenge 1 is to reduce the amount of initial data required, the experiments should emphasize the comparison of the amount of data needed for training/meta-training between MetaWearS and traditional methods. The current focus on outperforming other methods under limited data conditions does not fully address the claim regarding data reduction. A more direct comparison showing how much data is required for MetaWearS to achieve satisfactory performance versus other methods would strengthen the argument.
Contradictory Wording: There is some confusion in the wording of the paper, particularly concerning the size of the base dataset. Although one of MetaWearS's claimed benefits is reducing initial data collection, section 2.2 describes the base dataset using terms like "large number" and "extensive," which seem contradictory. Clarifying this discrepancy would improve the coherence of the narrative.
Novelty in Quantization: The main idea of reducing memory usage in MetaWearS is through quantization, but quantization is a well-established technique. If the approach taken in MetaWearS extends beyond simple quantization, it would be beneficial to highlight this in section 2.4.1 to distinguish the method from existing practices.

Question for authors:
Comparison with Other Meta-Learning Methods: What differentiates MetaWearS from other meta-learning methods like Model-Agnostic Meta-Learning (MAML)? The problem setup appears similar, except that this study focuses on the wearable domain. It would be useful to clarify any unique aspects of MetaWearS in comparison to these established methods.
Motivation for Advanced Techniques: Traditional methods, including rule-based and statistical models, have shown to work effectively in ECG applications such as atrial fibrillation detection [1]. What is the specific motivation behind employing more advanced techniques in this context? For instance, could you discuss the potential benefits of using deep-learning based methods over simpler approaches in the wearable domain?

Visualization of Test Set in Figure 9b: In Figure 9b, the visualization of the test set does not show clear separability of abnormal data points. Could you provide further explanation or clarification on this before making the claim about enhanced separability? This would help readers better understand the effectiveness of the proposed method.
Performance Decline with One-Shot Learning: The observed decline in performance with one-shot learning is intriguing. Section 3.1 does not delve deeply into this phenomenon. Is this decline typical in few-shot learning scenarios, or is it unique to this specific application? A more detailed exploration of this behavior would be valuable.

Overall Rating: Major Revision
The paper presents an innovative approach to addressing challenges in wearable systems using meta-learning. However, some areas need further clarification and focus to strengthen the paper's claims and contributions. Addressing the weaknesses and providing more detailed explanations in response to the questions raised would significantly improve the paper's impact and readability.

Tateno, K., & Glass, L. (2001). Automatic detection of atrial fibrillation using the coefficient of variation and density histograms of RR and ΔRR intervals. Medical and Biological Engineering and Computing, 39, 664-671.

Reviewer #2

(Remarks to the Author)
The paper is well written, has clear structure, and the main contributions seem solid. The results have been analysed and visualised explicitly. Here are some comments
1. The few-shot learning strategies make sense to me, including the updates of prototype instead of all weights. In the contribution, it claims that 'MetaWearS can update the models with minimal additional labeled data when end users are actively using the wearable system.' Where does this 'minimal' come from? Can you prove in theory it is minimal?
2. In the contribution section, it claims we propose an update process involves modifying prototypes without replacing the entire model. Is it new and unique contribution of this paper, or some other papers also developed similar ideas, like [14]?
3. Does the target dataset need to be annotated by clinicians? If yes, what is the necessary annotated proportional (limited) target dataset? The annotation can be done normally offline and time consuming. Does it mean that MetaWears cannot be run real-time and requires clinician-in-the-loop?
4. MetaWears updates prototype instead of weights to save time and battery. What about computational complexity, will it affect modelling time and battery?
5. Here are some related references in few-shot and meta learning in healthcare:
Reprint of: Few-shot learning for cardiac arrhythmia detection based on electrocardiogram data from wearable devices
Personalized blood glucose prediction for type 1 diabetes using evidential deep learning and meta-learning
Few-shot transfer learning for wearable IMU-based human activity recognition
6. The discussion and ablation study were presented well. The methods include lots of necessary details. Well done.

Reviewer #3

(Remarks to the Author)
The paper introduces MetaWearS, a meta-learning framework designed to address key challenges in wearable health monitoring systems: limited labelled data, inefficient model updates, and high energy consumption. By leveraging Prototypical Networks for few-shot learning, MetaWearS enables effective detection of biomedical abnormalities (e.g., in two key examples, such as epilepsy and atrial fibrillation) with minimal initial training data.

Strengths:
- The paper is very relevant in wearables and low-power embedded systems. The objectives and contributions are very well stated and significant. It addresses real-world challenges
- I appreciate the idea of using meta-learning as a way to reduce the dependency on large labelled datasets, which I agree with authors, in the field of healthcare, are challenging to find, particularly in EEG
- The use of a RISC-V-based system for implementation adds a practical contribution that makes the deployability of the method proposed more credible.
- The results obtained are promising.
- The paper is clear and well-written.

Weaknesses
- Although the authors demonstrate the method's applicability across two distinct biomedical signal types, the paper does not dissipate doubt about the method's generalization ability. Can authors better discuss this aspect? For example, in EEG, several kinds of analysis are more affected, if possible, by the variability of data and the need to calibrate the data on different patients. How does this method generalize in these cases?
- Is the choice of 3 quantities per case done in section 2.2.1 sufficient? How have the 5,15,25 for AF and 1,3,5 for epilepsy been chosen? What is the medical reference? Can the authors justify better or expand?
- What happens in cases with more complex signals, e.g. multi-channel or multimodal signals? What happens in the case of non-binary classifications? Please comment on the scalability of the method.
- Is the choice of Prototypical Networks the only possible? What about other methods like Relation Networks or Matching Networks?

- The datasets used (e.g., TUH and PhysioNet) are mostly from controlled environments (e.g., hospitals). The authors also use data from a wearable device in one of the 2 cases. However, how much of the data used is pre-processed and clean? Have the authors considered a test on more "dirty" datasets closer to real-world settings? The authors honestly admit this limitation in section 3.3. Can authors anyway provide more details on the robustness of the approach in non-controlled environments?
- Strictly related to the previous comment. The discussion should also provide a more critical reflection on the limitations of the proposed approach, such as its dependency on the quality of the feature space, sensitivity to noise or challenges in adapting to highly imbalanced datasets.
- While the paper compares MetaWearS with a conventional approach, it doesn't benchmark against other state-of-the-art meta-learning techniques. A broader comparative analysis would strengthen the claims.
-Although the paper emphasizes energy efficiency, it doesn't fully address the trade-offs involved in computational demands, memory usage, or latency for different hardware platforms. I confirm I appreciated the deployment of MetaWearS on the RISC-V hardware. However, as a minor shortcoming, a deployment (or an applicability evaluation) also in a more standard and easy-to-find platform would have increased the value of the demonstration.
- In my personal opinion, the organization of the paper should be revised. I'm not sure if this is a choice to align with the structure of other articles of this target journal or if it is a choice of the authors. However, I had to read section 4 to fully understand sections 3 and 2. I would have preferred a more traditional organization such as Abstract, Intro, SoA/related works and background info, methods, results, discussion and conclusion. In particular, "methods" before and not after the "results" would have helped better understand the results themselves. Some of my comments may be caused by having lost information due to the paper's organisation.

Some additional comments:
- Figure 6 is unclear - can authors improve the caption and the in-text comment? What is the red line? What are the points? Etc.
- p.5 at the beginning "... convention approach one..." is the word convention correct? Or was it conventional? What is approach one? I apologize. I do not understand the sentence and the reference.
- The choice of colour for the test set points in plot 9a is not very helpful (being red and purple very similar)

Version 1:

Reviewer comments:

Reviewer #1

(Remarks to the Author)
My comments were adequately addressed.

Reviewer #2

(Remarks to the Author)
The revised paper addressed most of my questions well. I recommend this article to be published.

Reviewer #3

(Remarks to the Author)
After reviewing the revised manuscript and the associated rebuttal letter, considering also comments from other reviewers. I appreciate the authors' substantial effort in addressing all reviewer comments.
The authors have significantly improved the manuscript by clarifying concepts and adding missing details. They also added novel experimental results (e.g. adding noise on EEG signal) and comparisons with alternative techniques (e.g., MAML, Matching Networks, Relation Networks). The manuscript is well written, the figures are clearer, and the new results make the paper more compelling.
The generalization discussion has been clearly expanded and now properly addresses patient variability and the limits of transfer between datasets. As a minor suggestion one sentence acknowledging that results are based on two binary detection tasks would be a reasonable and transparent addition. This is optional refinement; the manuscript is already solid without it.

Overall, the revision is strong, thorough, and of high quality.

Open Access This Peer Review File is licensed under a Creative Commons Attribution 4.0 International License, which permits use, sharing, adaptation, distribution and reproduction in any medium or format, as long as you give appropriate credit to the original author(s) and the source, provide a link to the Creative Commons license, and indicate if changes were made.
In cases where reviewers are anonymous, credit should be given to 'Anonymous Referee' and the source.
The images or other third party material in this Peer Review File are included in the article's Creative Commons license, unless indicated otherwise in a credit line to the material. If material is not included in the article's Creative Commons license and your intended use is not permitted by statutory regulation or exceeds the permitted use, you will need to obtain permission directly from the copyright holder.
To view a copy of this license, visit https://creativecommons.org/licenses/by/4.0/

MetaWearS: A Shortcut in Wearable Systems Lifecycle with Only a Few Shots

We are writing to resubmit our revised manuscript titled "MetaWearS: A Shortcut in Wearable Systems Lifecycle with Only a Few Shots" (Manuscript ID: COMMSMED-24-0848-T.) for publication in Nature Machine Intelligence.

We sincerely thank the Editor and the Reviewers for dedicating their time and expertise to evaluating our manuscript. We found the feedback to be extremely constructive and insightful and we appreciate the valuable suggestions provided to improve our work.

We have carefully addressed all the comments made by the reviewers and made substantial revisions to the manuscript. We believe that these changes have significantly enhanced the paper's clarity, rigor, and overall contribution. The revised manuscript is attached with this letter and all modifications and additions made to the original text have been highlighted in yellow for ease of identification.

In line with the reviewers' suggestions, we have incorporated extensive clarifications throughout the manuscript to address specific queries regarding our methodology, the novelty of our approach, practical implementation details, and the interpretation of our findings.

Furthermore, we have conducted several new experiments to provide additional empirical support for our claims and to address specific points raised during the review process. These new experiments include the following:

1. **Comparison with Target-Only Supervised Baseline:** Explicitly quantifying the reduction in target-specific labeled data needed by MetaWearS compared to traditional supervised learning.
2. **MetaWearS vs. other meta-learning baselines:** Comparison of MetaWearS performance against MAML, Matching Network, and Relation Network, relevant state-of-the-art meta-learning baselines.
3. **Expanded Hardware Evaluation:** Deploying and evaluating the proposed MetaWearS on additional common low-power hardware platforms.
4. **Update Computation Cost Analysis:** Quantifying the computational time required for the prototype update step.
5. **Noise Robustness Evaluation:** Assessing the model performance under simulated real-world noise conditions.

The results of these new experiments have been integrated into the revised manuscript.

We trust that the revised manuscript now comprehensively addresses the concerns of the reviewers. A detailed point-by-point response outlining the specific changes made in relation to each comment follows this cover letter.

We are grateful for the opportunity to revise our manuscript and hope that the improved version will now be suitable for publication in Nature Communication Medicine.

Sincerely,

Alireza Amirshahi, Maedeh H. Toosi, Siamak Mohammadi, Stefano Albini, Pasquale Davide Schiavone, Giovanni Ansaloni, Amir Aminifar, and David Atienza

Reviewer #1

Summary of the paper: The paper introduces MetaWearS, a meta-learning framework designed to address the challenges of data scarcity and resource efficiency in wearable systems. The authors propose a method that requires minimal initial data collection and an efficient updating mechanism that enhances the lifecycle of wearable devices. Through case studies on epilepsy and atrial fibrillation detection, the paper demonstrates the efficacy of MetaWearS in reducing data requirements while maintaining high performance.

Strength of the paper:

- **Reproducibility:** The paper provides a thorough description of the experimental setup, including hardware details, experiment hyperparameters, and data preprocessing procedures. This enhances the reproducibility of the study, which is helpful for validating the proposed method, even though part of the data remains private.
- **Clear Organization:** The paper is well-organized, with a logical flow that makes it easy to follow the authors' arguments and experimental results. This clarity supports a better understanding of the complex methodologies employed.
- **Energy Efficiency:** One of the significant contributions is the reduction in energy consumption during model updates, which is critical for the deployment of deep learning models on resource-constrained wearable devices. The paper demonstrates a substantial reduction in power usage, which is a strong point for practical applications.

We sincerely appreciate the reviewer acknowledging the strengths of our paper. We have aimed to further enhance these aspects in the revised manuscript. Reproducibility is further supported by our updated, documented and publicly available code (`https://github.com/esl-epfl/metawears`) with detailed descriptions. In addition, the energy efficiency claims are further substantiated by new results from deployment on additional hardware platforms.

Weakness of the paper:

- **Experimental Focus:** If the primary claim of MetaWearS in addressing challenge 1 is to reduce the amount of initial data required, the experiments should emphasize the comparison of the amount of data needed for training/meta-training between MetaWearS and traditional methods. The current focus on outperforming other methods under limited data conditions does not fully address the claim regarding data reduction. A more direct comparison showing how much data is required for MetaWearS to achieve satisfactory performance versus other methods would strengthen the argument.

  We thank the reviewer for this suggestion. To directly address this point and quantify the data reduction benefit of MetaWearS for initial training (Challenge ①), we have performed a new experiment.

  New Experiment Description: We compared MetaWearS with a standard supervised learning baseline (trained **only** on the target dataset with traditional ML methods). We incrementally increased the amount of labeled data provided to the supervised baseline and measured performance (AUC). We determined the amount of target data that the baseline required to achieve a performance level comparable to MetaWearS performance. The test set, comprising 20% of the target data set, remained consistent in all experiments to ensure a fair comparison.

  Results Summary: For the AF detection task, we conducted this experiment using two architectures with the supervised baseline.

  In the first baseline experiment, we utilized the same architecture as MetaWearS. Due to the scarcity of initial data in the target dataset, this model shows significant overfitting even with a gradual increase in data size. This phenomenon prevents the baseline model from achieving satisfactory performance.

  For the second experiment, to enable a more meaningful comparison and achieve a comparable performance trajectory, we employed a more compact MobileNet-style architecture with a lower number of parameters than MetaWearS. As mentioned in the manuscript, this architecture was used in [38]. The results of training this model solely on the target dataset are presented in Figure 1. The x-axis represents the minutes of labeled signal from the target dataset used for training/fine-tuning, and the y-axis represents the Area Under the Curve (AUC) performance.

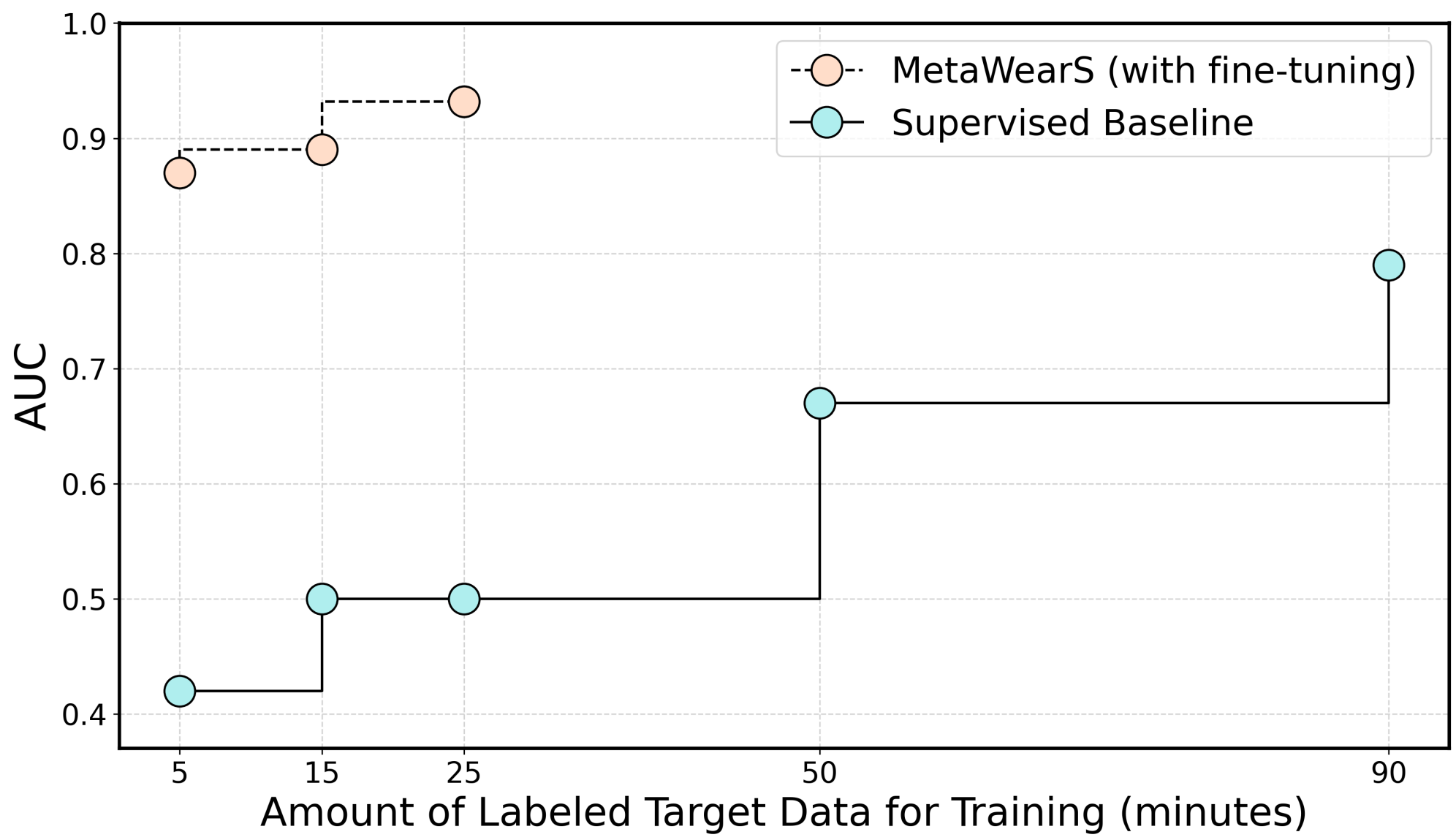

Figure 1: Initial data increase in AF

As illustrated in Figure 1, MetaWearS achieves an AUC of 89% with only 5 minutes of the labeled ECG signal from the target dataset. In stark contrast, the supervised baseline model, with its reduced complexity, only began to show improvement at 50 minutes of signal, reaching 68.2% AUC. Furthermore, it could not achieve an AUC of 80% even with 90 minutes of training data. This clearly demonstrates that MetaWearS significantly reduces the amount of initial labeled data required to achieve high performance.

Manuscript Addition: Based on this experiment, we have added a new subsection detailing this analysis and its results. We added this as Subsection 2.2.3, titled "Quantifying initial data reduction", following the initial results presentation. The added text is highlighted below:

> **2.2.3 Comparison with Target-Only Supervised Baseline:** To explicitly quantify the benefit of MetaWearS in reducing the need for target-specific labeled data (Challenge ①), we compared its performance against a Target-Only Supervised Baseline. This baseline model was trained from scratch using only the labeled data from the target dataset. We incrementally increased the amount of training data available to the baseline, from a few minutes up to 90 minutes of signal, to observe the performance trajectory. We first attempted to use a model with the same architecture as MetaWearS for the baseline; however, due to the severe scarcity of initial data, this model showed significant overfitting and failed to achieve a satisfactory performance level. To facilitate a more meaningful comparison, we then employed a more compact MobileNet-style architecture with fewer parameters for the baseline, as used in [38], which is better suited for training on smaller datasets. The test set was kept consistent across all experiments to ensure a fair evaluation.
>
> The results for the AF detection task are presented in Fig. 7. The figure plots AUC against the minutes of labeled signal used for training. As illustrated, MetaWearS achieves a high performance of 89% AUC using only 5 minutes of the labeled ECG signal from the target user. In stark contrast, the supervised baseline requires significantly more data to even begin learning effectively. It only starts to show a significant improvement after 50 minutes of signal, reaching an AUC of 68.2%. Even when provided 90 minutes of training data, the baseline performance remains below the 80% AUC, failing to reach the level achieved by MetaWearS with a fraction of the data.
>
> This result clearly quantifies the data-efficiency of our approach. MetaWearS can achieve high accuracy with a lower amount of initial data, whereas a traditional approach would

require a substantially larger, and in this case prohibitively large, dataset to approach comparable performance. This directly validates our claim that MetaWearS effectively addresses the challenge of reducing the initial data burden for personalizing wearable sensor models.

References: [38] Kim, S., Chon, S., Kim, J.-K., Kim, J., Gil, Y. and Jung, S. Lightweight Convolutional Neural Network for Real-Time Arrhythmia Classification on Low-Power Wearable Electrocardiograph in 44th Annual International Conference of the IEEE Engineering in Medicine and Biology Society (EMBC) (2022), 1915–1918.

- **Contradictory Wording:** There is some confusion in the wording of the paper, particularly concerning the size of the base dataset. Although one of MetaWearS's claimed benefits is reducing initial data collection, section 2.2 describes the base dataset using terms like "large number" and "extensive," which seem contradictory. Clarifying this discrepancy would improve the coherence of the narrative.

  We thank the reviewer for highlighting this potential point of confusion. We apologize for the lack of clarity. The core idea of MetaWearS is indeed to reduce the need for collecting extensive labeled data, but specifically data collected **from the target wearable device/user**, which is typically scarce. This reduction is achieved precisely by leveraging a separate, potentially **large and extensive base dataset** (e.g., from hospital settings) for effective pre-training. The base dataset provides general knowledge, which is then adapted using only a low amount of target data.

  Manuscript Modification: To ensure this distinction is crystal clear throughout the manuscript, we have added clarifications in **two key locations**:

  1. In the **Introduction section** (page 2) where we introduce how MetaWearS addresses the challenges. The modified paragraph, with the clarification highlighted, now reads:

  > Remarkably, as shown in Fig.1b, our approach reduces the data required for the initial training of the model (①) and for the update process (②). This is achieved through a targeted modification of the few-shot learning strategy, specifically designed to address the aforementioned challenges. Specifically, MetaWearS reduces the required amount of *target-specific* labeled data (i.e., data collected directly from the end-user's wearable device, which is often scarce) by effectively leveraging knowledge transferred from a separate, potentially large and more readily available base dataset (e.g., existing clinical datasets). The use of an extensive base dataset for pre-training is therefore key to minimizing the need for target data collection, resolving the apparent contradiction.

  2. In the **Results section** where the base and target datasets are formally defined, the added clarification is highlighted below:

  > In this section, we tackle Challenge ①... with a minimal amount of annotated signals in the target dataset. To achieve this, in our work, we use a "base dataset", which contains a large number of signals... albeit in a different setting. Fig. 3a shows the base dataset. The base dataset is an extensive dataset.... Crucially, the goal of MetaWearS is to minimize the data required from the scarce *target* dataset (Fig. 3b) by effectively leveraging this larger *base* dataset for pre-training; the potentially large size of the base dataset does not contradict the primary objective of reducing target data needs.

  We believe that adding these clarifications in both the introductory framing and the specific dataset definitions section comprehensively addresses the reviewer's concern and improves the manuscript's coherence.

- **Novelty in Quantization:** The main idea of reducing memory usage in MetaWearS is through quantization, but quantization is a well-established technique. If the approach taken in MetaWearS extends beyond simple quantization, it would be beneficial to highlight this in section 2.4.1 to distinguish the method from existing practices.

  We thank the reviewer for this comment regarding the novelty related to quantization. We agree that quantization is a well-established technique, and our paper does not claim novelty in the quantization

method itself. As the reviewer implicitly notes, the quantization concept is primarily discussed in the context of our hardware implementation results (Section 2.4.1, associated Tables 1 & 2), rather than being a core part of the Introduction or Methods sections defining the MetaWearS algorithm.

Instead, we employed standard quantization techniques (specifically, 16-bit fixed-point with hybrid floating-point for non-linear operations in the epilepsy case) as an essential *enabling technology* for deploying our models efficiently on the resource-constrained X-HEEP hardware platform. The primary novelty of MetaWearS lies in the meta-learning framework designed for the wearable lifecycle, the few-shot learning strategy adapting to new users with low amount of data, and particularly the efficient *prototype-based update mechanism* which drastically reduces data transmission and energy consumption for model updates. Quantization supports the feasibility of running the inference and receiving updates on the low-power hardware.

Manuscript Modification: To ensure this is clear, we have added a clarifying sentence to the hardware implementation section discussing memory optimization (Section 2.4.2). The addition is highlighted below:

> We emphasize that while quantization is crucial for efficient deployment on resource-constrained hardware like X-HEEP, the quantization techniques themselves are standard practices. The core novelty of our work lies in the MetaWearS framework for few-shot learning and efficient prototype-based updates within the wearable systems lifecycle.

Question for authors:

- **Comparison with Other Meta-Learning Methods:** What differentiates MetaWearS from other meta-learning methods like Model-Agnostic Meta-Learning (MAML)? The problem setup appears similar, except that this study focuses on the wearable domain. It would be useful to clarify any unique aspects of MetaWearS in comparison to these established methods.

  We thank the reviewer for this question and for suggesting a comparison with other established few-shot learning methods like MAML. To directly address this, we performed a new experiment (Experiment #2 in our planning) comparing MetaWearS with MAML on our specific tasks and datasets.

  The core distinction of MetaWearS lies not in achieving superior classification accuracy alone, but in being architected for the unique lifecycle constraints of wearable systems. In particular, in addition to the initial training data (Challenge ①), it also addresses the data requirements for subsequent model updates (Challenge ②) and, most critically, the energy efficiency of the update protocol itself (Challenge ③). Established methods like MAML, while powerful, were designed for general few-shot accuracy and do not address these crucial lifecycle considerations for resource-constrained wearable devices.

  To directly investigate the trade-offs, we performed a new comparative analysis against MAML, Matching Networks, and Relation Networks. The goal of this experiment was to answer a critical question: *Can MetaWearS deliver its lifecycle efficiency benefits without compromising the few-shot classification performance established by these state-of-the-art methods?*

  New Experiment Summary & Findings: We implemented MAML using the same feature extractor backbones (Vision Transformer for epilepsy, and MobileNetV2-inspired CNN for AF) as MetaWearS. The learning difference between the two methods immediately highlights the superiority of our MetaWearS Prototypical Network-based approach for the wearable lifecycle (Challenges ② & ③). MAML adapts by learning an initialization for gradient-based fine-tuning of the model's weights. During deployment, any update would require further computationally-intensive gradient steps and the wireless transmission of the entire set of updated weights. MetaWearS, in stark contrast, performs updates by simply recalculating the class prototypes from new data shots and transmitting only these extremely compact vectors. This distinction is the source of our method's primary advantage: as detailed in our hardware analysis, it leads to **orders-of-magnitude reduction (418x-456x)** in the energy consumption required for an update.

  Similar to the methodology used in Section 2.2.2 to compare our work with the source-only baseline, both MetaWearS and MAML methods were pre-trained on the respective base datasets (TUSZ EEG,

PhysioNet ECG) using their standard meta-training procedures. We then evaluated their performance on the few-shot fine-tuning task using the target datasets (Siena EEG, private wearable ECG). For the seizure detection task, we obtained that the classification performance of MetaWearS is not statistically different with MAML ($p > 0.05$), and MetaWearS performs better with an average of 0.9% of AUC. This demonstrates that our efficiency gains come with no loss in accuracy in this scenario. Furthermore, for the more challenging AF detection task, which involves a greater domain shift between the base and target datasets, MetaWearS demonstrated a statistically significant **improvement** in performance over MAML, with an average AUC improvement of 6.4% ($p<0.05$).

These results empirically confirm that our prototype-based approach not only enables the immense energy savings critical for the wearable lifecycle but does so while maintaining a competitive, and in some cases superior, classification accuracy.

This fundamental advantage in lifecycle efficiency extends beyond optimization-based methods and holds true when comparing MetaWearS to other prominent metric-based approaches, namely Matching Networks and Relation Networks. More importantly, these alternative architectures share a critical limitation regarding lifecycle efficiency (Challenges ② and ③). Unlike MetaWearS, which condenses all support set information into a single prototype, both Matching and Relation Networks require the feature vectors of *every individual sample* in the support set to perform a classification. Consequently, to update the system with k new shots, all k feature vectors must be transmitted, a process that is k times less efficient than the single prototype update of MetaWearS.

While establishing this key efficiency advantage, our analysis also confirmed that it does not come at the expense of accuracy. In case of AF detection, MetaWearS demonstrated competitive performance, significantly outperforming Matching Networks with a mean AUC improvement of 2.8% ($p < 0.05$) and achieving an accuracy similar with the highly competitive Relation Network, with a mean improvement of 1.7% ($p > 0.05$). This unique combination of high performance and an architecturally superior, energy-efficient update mechanism solidifies the value of the MetaWearS framework.

In summary, what differentiates MetaWearS is its unique prototype-based architecture, which is explicitly co-designed for the practical constraints of the wearable lifecycle. This design provides an ultra-lightweight and energy-efficient update mechanism—leading to orders-of-magnitude energy savings—while our new experiments confirm that it simultaneously maintains a classification accuracy that is on-par with, or superior to, established meta-learning benchmarks like MAML, Matching Networks, and Relation Networks.

Manuscript Addition: We have added a new subsection (Section 3.1) to the manuscript to detail this comparative analysis and integrate these findings. The added text is provided below.

> **Comparison with other Few-Shot Learning Methods**
>
> To contextualize the performance of MetaWearS, we benchmarked it against several established few-shot learning algorithms, including MAML, Matching Networks, and Relation Networks. Our analysis confirms that MetaWearS provides its significant lifecycle efficiency benefits without compromising on classification performance.
>
> **Comparison with MAML.** The primary distinction lies in the update mechanism, which directly impacts the wearable lifecycle (Challenges ② & ③). MAML adapts by fine-tuning its model weights via gradient descent, requiring the wireless transmission of the entire updated model for on-device deployment. In stark contrast, MetaWearS leverages its Prototypical Network foundation to perform updates by simply recalculating and transmitting a single, compact prototype vector. This architectural difference leads to an orders-of-magnitude reduction (418x-456x) in the energy required for model updates.
>
> Having established this efficiency advantage, for the performance comparison, we followed a methodology similar to the one used in Section 2.2.2. All methods were first pre-trained on their respective base datasets (TUSZ EEG, PhysioNet ECG) using their standard meta-training procedures. Subsequently, we evaluated their few-shot classification performance on the corresponding target datasets (Siena EEG, private wearable ECG).
>
> Our experiments confirm that the MetaWearS advantage on update efficiency does not come at the cost of performance. For the seizure detection task, the classification performance of

MetaWearS was not statistically different with MAML ($p > 0.05$), and MetaWearS has a mean AUC improvement of 0.9%. For the more challenging AF detection task, which involves a greater domain shift, MetaWearS demonstrated a statistically significant **improvement** over MAML, with an average AUC improvement of 6.4% ($p < 0.05$). This performance gap can be attributed to MAML's known vulnerability to overfitting on small support sets, whereas the implicit regularization of our prototype-based approach ensures greater robustness.

Comparison with other Metric-Based Methods. This fundamental advantage in lifecycle efficiency extends to other prominent metric-based approaches, namely Matching Networks and Relation Networks. These architectures share a critical limitation: unlike MetaWearS, which condenses all support set information into a single prototype, they require the feature vectors of *every individual sample* in the support set for classification. To update the system with k new shots, all k feature vectors must be transmitted, a process that is k times less efficient than the single prototype update of MetaWearS.

While being architecturally more efficient, MetaWearS also demonstrated competitive accuracy. In the case of AF detection, it significantly outperformed Matching Networks with a mean AUC improvement of 2.8% ($p < 0.05$) and achieved an accuracy similar to the highly competitive Relation Network, with a mean improvement of 1.7% ($p > 0.05$). This unique combination of validated high performance and an architecturally superior, energy-efficient update mechanism solidifies the value of the MetaWearS framework for practical wearable systems.

- **Motivation for Advanced Techniques:** Traditional methods, including rule-based and statistical models, have shown to work effectively in ECG applications such as atrial fibrillation detection. What is the specific motivation behind employing more advanced techniques in this context? For instance, could you discuss the potential benefits of using deep-learning based methods over simpler approaches in the wearable domain?

  We thank the reviewer for raising this important point regarding the motivation for using advanced Deep Learning (DL) and meta-learning techniques like MetaWearS, especially given the established effectiveness of traditional methods in certain areas like AF detection. Our motivation is driven by the potential of DL to capture complex patterns and adapt to diverse data typically encountered in wearable scenarios, provided the significant challenges associated with DL deployment (data needs, resource constraints) can be overcome – which is the primary goal of MetaWearS.

  While traditional methods are valuable and effective for specific, well-defined tasks, DL-based approaches offer several potential advantages, particularly for versatile, long-term wearable monitoring:

  - **Automatic Feature Extraction:** DL models can learn relevant features directly from raw or minimally processed signals, potentially capturing complex or subtle patterns missed by methods relying on pre-defined, hand-engineered features. This reduces the need for domain-specific feature crafting.
  - **Handling Complexity and Variability:** They excel at modeling non-linear relationships and can potentially adapt better to inter-patient variability and diverse signal characteristics encountered across different individuals and time periods using techniques like fine-tuning.
  - **Potential for Higher Performance:** DL methods often achieve state-of-the-art performance on complex signal classification benchmarks.
  - **End-to-End Learning:** They facilitate an end-to-end learning approach, simplifying the pipeline from signal acquisition to diagnosis.

  The challenge lies in making these powerful DL techniques practical for wearables, given their data hunger and computational demands. MetaWearS specifically targets these challenges (limited target data, efficient updates, energy constraints) to unlock the potential benefits of DL for wearable health monitoring.

Manuscript Modification: To explicitly address this motivation within the paper, we have added a dedicated paragraph in the **Introduction section** (Section 1). The added text, highlighted below, discusses the rationale for exploring DL in this context:

> While traditional methods are effective [15], deep learning offers potential advantages like automatic learning of complex patterns from signals and adaptation to patient variability [16,17]. However, deep learning data and resource demands hinder its use in wearables. MetaWearS is motivated by the goal of overcoming these specific challenges (data scarcity, update efficiency) to make the benefits of advanced deep learning techniques practical for wearable health monitoring.

We believe this addition clarifies the motivation driving our research direction.

References: [15] Tateno, K & Glass, L. Automatic detection of atrial fibrillation using the coefficient of variation and density histograms of RR and RR intervals. Medical and Biological Engineering and Computing 39, 664–671 (2001).

[16] Xia, Y., Wulan, N., Wang, K. & Zhang, H. Detecting atrial fibrillation by deep convolutional neural networks. Computers in biology and medicine 93, 84–92 (2018).

[17] He, R., Wang, K., Zhao, N., Liu, Y., Yuan, Y., Li, Q. & Zhang, H. Automatic detection of atrial fibrillation based on continuous wavelet transform and 2D convolutional neural networks. Frontiers in physiology 9, 1206 (2018).

- **Visualization of Test Set in Figure 9b:** In Figure 9b, the visualization of the test set does not show clear separability of abnormal data points. Could you provide further explanation or clarification on this before making the claim about enhanced separability? This would help readers better understand the effectiveness of the proposed method.

  We thank the reviewer for their careful observation regarding the visualization in Figure 9b. We agree that the 2D Principal Component Analysis (PCA) projection, particularly for the AF detection task shown in Figure 9b, does not depict a perfect separation of the test set samples, especially the abnormal ones.

  PCA is a linear technique aimed at reducing dimensionality for visualization purposes, and this process can inevitably lead to a loss of information. The separability achieved by the model in the original high-dimensional feature space (e.g., D=128 for the ECG model's embedding) might not be fully preserved or apparent in the 2D visualization.

  Therefore, the claim of "enhanced separability" should be primarily understood through the lens of the quantitative classification performance metrics. The high Area Under the Curve (AUC) values achieved on the test set, as reported in other figures [e.g., Fig 5, 6, 8, 11], provide strong evidence that the model, after fine-tuning, can effectively distinguish between the classes in the learned feature space, even if this is not perfectly clear in the 2D PCA plot. The PCA plot serves more to illustrate the relative positions and clustering of the support set samples and the learned prototypes.

  Manuscript Modification: To address the reviewer's valid point, we have revised the discussion related to this figure (Section 3.2) to be more precise about what the visualization shows and to explicitly link the separability claim to the quantitative results. The modified text and caption include clarifications highlighted below:

  > Fig. 10 presents PCA visualizations of the feature embeddings for support and query (test) data after fine-tuning. While the 2D projection may not fully capture the separability in the original high-dimensional space, particularly for the dispersed AF test samples, the visualization illustrates the relative positioning of support data clusters and class prototypes after fine-tuning. The effectiveness of the learned feature space in separating the classes is quantitatively demonstrated by the high AUC performance achieved on the test set (see Section 2).

  We have also addressed the color choices in this figure based on Reviewer 3's comment. We hope this clarifies the interpretation of the visualization.

- **Performance Decline with One-Shot Learning:** The observed decline in performance with one-shot learning is intriguing. Section 3.1 does not delve deeply into this phenomenon. Is this decline typical in few-shot learning scenarios, or is it unique to this specific application? A more detailed exploration of this behavior would be valuable.

We thank the reviewer for their interest in this phenomenon and for suggesting a deeper exploration. This phenomenon **is not unique** to our application. It is a recognized challenge in the field of Incremental Few-Shot Learning (IFL) [65, 66, 67], often manifesting as a form of catastrophic forgetting [68, 69].

In our methodology, prototypes are updated by taking the average of the existing prototype, c_n, and the embedding of the single new sample. Therefore, the core of the problem lies in the followings:

1. **Statistical Instability:** A class prototype is a statistical estimator of the true class centroid. A single data point represents the smallest possible sample size and is therefore an inherently **high-variance** estimator. The updated prototype's position becomes entirely dependent on the stochasticity of that one sample. If this sample is an outlier, its influence is not sufficiently dampened by other samples, allowing it to disproportionately shift the prototype towards its erroneous location. This leads to a poor and non-representative model of the class.
2. **Catastrophic Forgetting in IFL:** This unstable update is a potent trigger for catastrophic forgetting, which is the tendency of a model to lose performance on previously learned tasks after being trained on a new one. The IFL setting, where a model must adapt to new data, is particularly susceptible to this issue. The introduction of a single, noisy prototype disrupts the carefully optimized geometry of the entire metric space, harming classification accuracy for the updated class.

Therefore, as shown in the existing literature [70, 71], they provide academic validation for our empirical findings, showing that a naive, bad update can be more harmful than no update at all. Our own analysis in Section 3.1 illustrates this. Our visualization in Figure 10 depicts the trajectory of these prototype updates. The figure shows that when only a single shot is introduced, the updated prototype deviates from the optimal alignment with both training and test data. However, as more shots are involved (e.g., $k \geq 3$), the averaging process provides statistical regularization. This leads to a more stable update, causing the prototypes to progressively align closer with the test data distribution. This visually confirms that a multi-shot update can successfully fine-tune the model where a one-shot update fails. We have added a concise paragraph to Section 3.1 to ensure this important context is clear to the reader.

Manuscript Modification:

> As seen in Fig. 11, when a single shot is received, the updated prototypes slightly deviate from the train and test samples. This deviation sometimes results in a slight performance decrease compared to the baseline (no update), as observed in Fig. 9. This result is not an anomaly but a known challenge in Incremental Few-Shot Learning [65-67], often described as a form of catastrophic forgetting [68, 69]. The core of the issue lies in the statistical properties of the update itself. A class prototype is an estimator of the true class centroid, and a single sample provides a high-variance, statistically unstable estimate. If this single sample is an outlier or atypical, it can corrupt the prototype's position, disrupting the learned geometry of the entire metric space and degrading overall performance. This observation aligns with previous studies [70, 71] showing that naive adaptation with extremely scarce data can be more detrimental than no adaptation at all.
>
> In contrast, as more shots are received and the prototypes are updated with these shots, the prototypes align more closely with the test sample line. This result shows that in MetaWearS a k-shot update (where $k \geq 3$) leverages the power of averaging to provide a more robust, lower-variance estimate of the class centroid, leading to a more stable and constructive adaptation that improves generalization.

We believe this detailed analysis addresses the reviewer's question about this intriguing behavior.

References:

[65] Mengye Ren, Renjie Liao, Ethan Fetaya, and Richard S Zemel. Incremental few-shot learning with attention attractor networks. In Proceedings of the 33rd International Conference on Neural Information Processing Systems, pages 5275–5285, 2019,

[66] Xiaoyu Tao, Xiaopeng Hong, Xinyuan Chang, Songlin Dong, Xing Wei, and Yihong Gong. Few-shot class-incremental learning. In Proceedings of the IEEE/CVF Conference on Computer Vision and Pattern Recognition, pages 12183–12192, 2020,

[67] Pratik Mazumder, Pravendra Singh, and Piyush Rai. Few-shot lifelong learning. In Proceedings of the AAAI Conference on Artificial Intelligence, volume 35, pages 2337–2345, 2021,

[68] Ian J Goodfellow, Mehdi Mirza, Da Xiao, Aaron Courville, and Yoshua Bengio. An empirical investigation of catastrophic forgetting in gradient-based neural networks. arXiv preprint arXiv:1312.6211, 2013,

[69] James Kirkpatrick, Razvan Pascanu, Neil Rabinowitz, Joel Veness, Guillaume Desjardins, Andrei A Rusu, Kieran Milan, John Quan, Tiago Ramalho, Agnieszka Grabska-Barwinska, et al. Overcoming catastrophic forgetting in neural networks. Proceedings of the national academy of sciences, 114(13):3521–3526, 2017,

[70] Chi Zhang, Nan Song, Guosheng Lin, Yun Zheng, Pan Pan, and Yinghui Xu. Few-shot incremental learning with continually evolved classifiers. In Proceedings of the IEEE/CVF Conference on Computer Vision and Pattern Recognition, pages 12455–12464, 2021,

[71] Shi, G., Chen, J., Zhang, W., Zhan, L. M., and Wu, X. M. (2021). Overcoming catastrophic forgetting in incremental few-shot learning by finding flat minima. Advances in neural information processing systems, 34, 6747-6761.

Reviewer #2:

The paper is well written, has clear structure, and the main contributions seem solid. The results have been analysed and visualised explicitly.

We greatly appreciate the reviewer acknowledging the strengths of our paper.

Here are some comments:

1. The few-shot learning strategies make sense to me, including the updates of prototype instead of all weights. In the contribution, it claims that 'MetaWearS can update the models with minimal additional labeled data when end users are actively using the wearable system.' Where does this 'minimal' come from? Can you prove in theory it is minimal?

   We thank the reviewer for this question. The term "minimal" is used in an **empirical and practical sense** throughout the paper. It highlights the contrast between the small number of newly labeled samples (shots) required by MetaWearS for effective model updates and the substantially larger datasets typically needed for retraining conventional models. Our experiments demonstrate this empirically for both the initial training phase (Challenge ①, Fig. 5) and the update phase (Challenge ②, Fig. 9).

   To provide concrete, quantitative evidence for our assertion that the term "minimal" is used in an empirical and practical sense, we have conducted a new experiment specifically designed to quantify this benefit in Challenge ①. While our original manuscript demonstrated this data efficiency in Figures 5 and 8, this new analysis directly addresses the reviewer's question by benchmarking the data requirements of MetaWearS against a traditional supervised learning baseline. The objective was to determine precisely how much labeled data a standard model, trained only on the target dataset, would need to achieve a performance level comparable to that of MetaWearS.

   New Experiment Description: We compared MetaWearS with a standard supervised learning baseline (trained **only** on the target dataset with traditional ML methods). We incrementally increased the amount of labeled data provided to the supervised baseline and measured performance (AUC). We determined the amount of target data that the baseline required to achieve a performance level comparable to MetaWearS performance. The test set, comprising 20% of the target data set, remained consistent in all experiments to ensure a fair comparison.

   Results Summary: For the AF detection task, we conducted this experiment using two architectures with the supervised baseline.

   In the first baseline experiment, we utilized the same architecture as MetaWearS. Due to the scarcity of initial data in the target dataset, this model shows significant overfitting even with a gradual increase in data size. This phenomenon prevents the baseline model from achieving satisfactory performance.

   For the second experiment, to enable a more meaningful comparison and achieve a comparable performance trajectory, we employed a more compact MobileNet-style architecture with a lower number of parameters than MetaWearS. As mentioned in the manuscript, this architecture was used in [38]. The results of training this model solely on the target dataset are presented in Figure 2. The x-axis represents the minutes of labeled signal from the target dataset used for training/fine-tuning, and the y-axis represents the Area Under the Curve (AUC) performance.

   As illustrated in Figure 2, MetaWearS achieves an AUC of 89% with only 5 minutes of the labeled ECG signal from the target dataset. In stark contrast, the supervised baseline model, with its reduced complexity, only began to show improvement at 50 minutes of signal, reaching 68.2% AUC. Furthermore, it could not achieve an AUC of 80% even with 90 minutes of training data. This clearly demonstrates that MetaWearS significantly reduces the amount of initial labeled data required to achieve high performance.

   Manuscript Addition: Based on this experiment, we have added a new subsection detailing this analysis and its results. We added this as Subsection 2.2.3, titled "Quantifying initial data reduction", following the initial results presentation. The added text is highlighted below:

   > **2.2.3 Comparison with Target-Only Supervised Baseline:** To explicitly quantify the benefit of MetaWearS in reducing the need for target-specific labeled data (Challenge ①), we compared its performance against a Target-Only Supervised Baseline. This baseline model

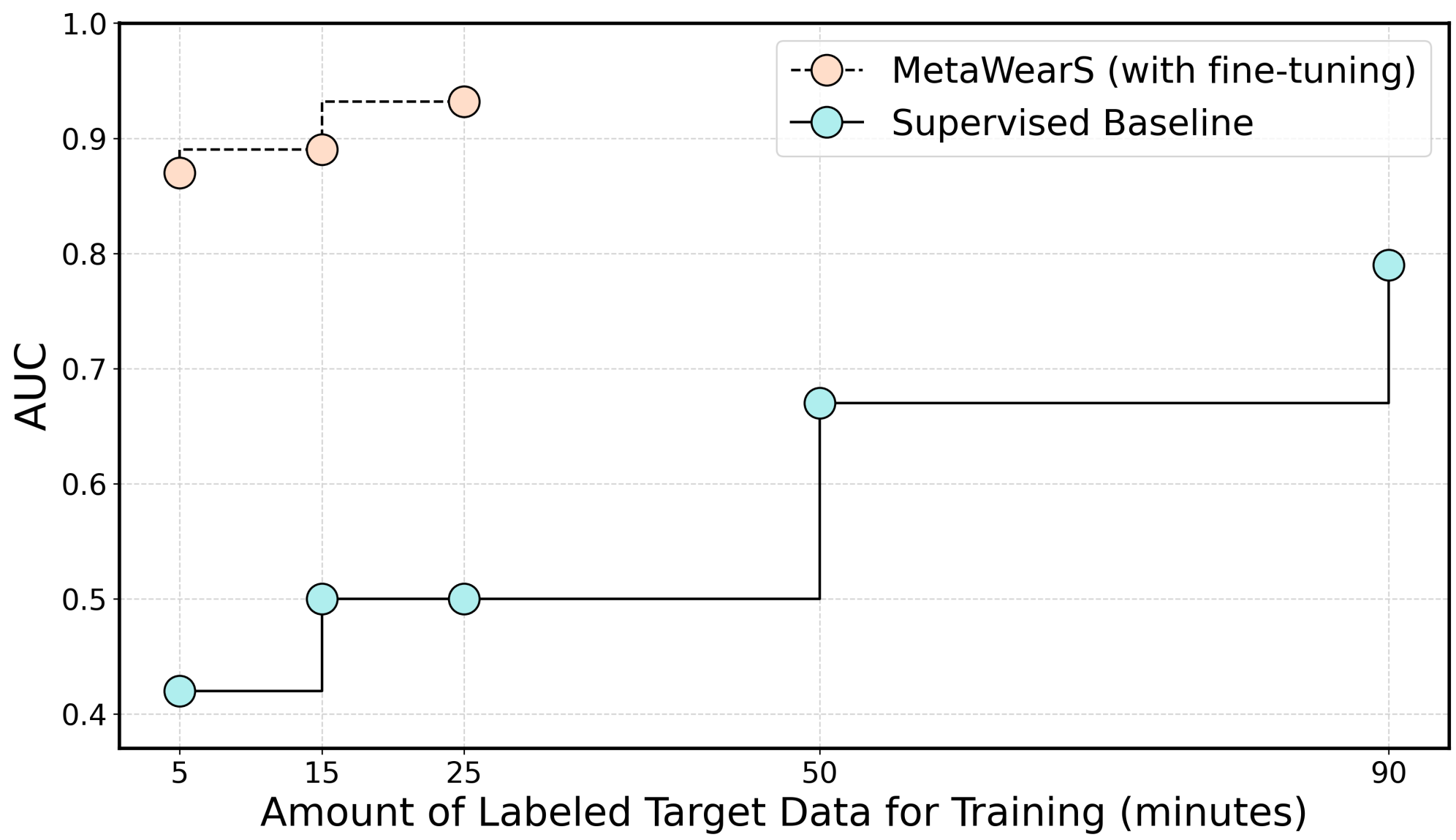

Figure 2: Initial data increase in AF

was trained from scratch using only the labeled data from the target dataset. We incrementally increased the amount of training data available to the baseline, from a few minutes up to 90 minutes of signal, to observe the performance trajectory. We first attempted to use a model with the same architecture as MetaWearS for the baseline; however, due to the severe scarcity of initial data, this model showed significant overfitting and failed to achieve a satisfactory performance level. To facilitate a more meaningful comparison, we then employed a more compact MobileNet-style architecture with fewer parameters for the baseline, as used in [38], which is better suited for training on smaller datasets. The test set was kept consistent across all experiments to ensure a fair evaluation.

The results for the AF detection task are presented in Fig. 7. The figure plots AUC against the minutes of labeled signal used for training. As illustrated, MetaWearS achieves a high performance of 89% AUC using only 5 minutes of the labeled ECG signal from the target user. In stark contrast, the supervised baseline requires significantly more data to even begin learning effectively. It only starts to show a significant improvement after 50 minutes of signal, reaching an AUC of 68.2%. Even when provided 90 minutes of training data, the baseline performance remains below the 80% AUC, failing to reach the level achieved by MetaWearS with a fraction of the data.

This result clearly quantifies the data-efficiency of our approach. MetaWearS can achieve high accuracy with a lower amount of initial data, whereas a traditional approach would require a substantially larger, and in this case prohibitively large, dataset to approach comparable performance. This directly validates our claim that MetaWearS effectively addresses the challenge of reducing the initial data burden for personalizing wearable sensor models.

References: [38] Kim, S., Chon, S., Kim, J.-K., Kim, J., Gil, Y. and Jung, S. Lightweight Convolutional Neural Network for Real-Time Arrhythmia Classification on Low-Power Wearable Electrocardiograph in 44th Annual International Conference of the IEEE Engineering in Medicine and Biology Society (EMBC) (2022), 1915–1918.

Furthermore, to enhance clarity and explicitly reflect the empirical nature of our claim, we have slightly modified the phrasing in the list of contributions in the Introduction (Section 1). The updated contribution now reads:

> The proposed MetaWearS can update the models with an *empirically* minimal amount of additional labeled data when end users are actively using the wearable system.

We believe this revised wording and the new experiment added to the manuscript accurately represents our findings.

2. In the contribution section, it claims we propose an update process involves modifying prototypes without replacing the entire model. Is it new and unique contribution of this paper, or some other papers also developed similar ideas, like [14]?

   We thank the reviewer again for this critical question regarding the novelty of our update mechanism.

   We acknowledge that the **core classification mechanism** using prototypes originates from Prototypical Networks [14] (which we cite). We also acknowledge that various strategies exist for **updating only partial model weights** (like final layers).

   However, our contribution is novel in its **architectural minimalism** and the specific nature of the update. To the best of our knowledge, our work is the first to formalize and evaluate a mechanism where the set of prototypes within a Prototypical Network serves as the **sole updatable layer**. We specifically evaluate this lightweight update mechanism for its suitability in frequent, on-device personalization scenarios on battery-powered wearable devices.

   Our contribution is different compared to the prior works:

   (a) **Update Mechanism Detail:** During the *update phase* post-deployment (Challenge ②/③), MetaWearS keeps the feature extractor network f_ϕ (already trained via meta-pre-training and few-shot fine-tuning) **frozen**. The update consists solely of: (i) embedding the few new shots using this frozen f_ϕ, (ii) recalculating the class prototypes by averaging these new embeddings with embeddings from the initial target support set (Section 4.4, Eq. 3), and (iii) wirelessly transmitting *only these compact prototype vectors* (Table 3) back to the wearable. This makes our method orders of magnitude faster and more energy-efficient, a decisive advantage for frequent personalization on battery-powered devices where the energy cost of backpropagation is a major barrier.

   (b) **Differentiation from Prior Efficiency Strategies:**

   - *vs. Prototypical Network [14]:* The original Prototypical Networks paper focused on learning a deep embedding function (f_ϕ) via episodic training to solve the few-shot classification problem. While its architecture provides a concise representation for each class, the paper does not propose or evaluate an update mechanism with a frozen encoder. Its entire learning algorithm is dedicated to optimizing the embedding space itself, not defining a post-training update rule.
   - *vs. Continual and Federated Learning:* A review of related work in Continual and Federated Learning reveals a consistent pattern: even when using frozen backbones, prior methods introduce separate, learnable components to handle new data. For example, they train secondary encoders [2], fusion layers [3], or projection layers [4]. In these architectures, prototypes are merely a tool used to guide the training of these separate modules. Our method is novel because it dispenses with these extra components entirely.
   - *vs. Partial Weight Updates:* Updating only the last layer involves transmitting layer weights and often uses backpropagation. MetaWearS transmits the derived prototypes themselves (potentially much smaller than even final layer weights, cf. Table 3) and uses a simpler forward-pass + averaging computation for the update calculation (see response to Q4).

   Manuscript Modification: We have updated the **Introduction and Discussion sections** to incorporate insights from the reviewed literature on prototype communication and partial updates, clearly positioning our specific contribution. The statement of contributions in the **Introduction section** (Section 1) remains refined as follows:

   > To the best of our knowledge, our work is the first to formalize and evaluate a mechanism where the set of prototypes within a Prototypical Network serves as the sole updatable layer. We specifically evaluate this lightweight update mechanism for its suitability in frequent, on-device personalization scenarios on battery-powered wearable devices.

We hope that this detailed explanation clarifies the novelty and significance of our approach, particularly concerning Challenge ③.

References: [2] Ren H, Cai Y, Chen X, et al. "A two-phase prototypical network model for incremental few-shot relation classification". Association for Computational Linguistics (ACL), 2020.

[3] Tan, Yue, Guodong Long, Jie Ma, Lu Liu, Tianyi Zhou, and Jing Jiang. "Federated learning from pre-trained models: A contrastive learning approach." Advances in neural information processing systems 35 (2022): 19332-19344.

[4] Zhang, Wei, and Xiaodong Gu. "Few shot class incremental learning via efficient prototype replay and calibration." Entropy 25.5 (2023): 776.

3. Does the target dataset need to be annotated by clinicians? If yes, what is the necessary annotated proportional (limited) target dataset? The annotation can be done normally offline and time consuming. Does it mean that MetaWears cannot be run real-time and requires clinician-in-the-loop?

We thank the reviewer for these important practical questions regarding annotation requirements and real-time operation. Let us address each point:

(a) **Annotation Requirement:** Yes, the reviewer is correct. Both the initial limited target dataset used for fine-tuning MetaWearS (Challenge ①) and the subsequent 'new shots' used for model updates (Challenge ②) require reliable ground-truth labels. In our context, these annotations would typically be provided by expert clinicians, as mentioned in the manuscript (e.g., Section 2.2, Section 2.3, Section 4.4). This supervision is necessary for the meta-learning process (fine-tuning) and for calculating the updated prototypes correctly.

(b) **Necessary Proportion:** Our work does not define a strict minimum proportion or percentage of annotated data required. Instead, we focused on demonstrating the core benefit of MetaWearS, which is its effectiveness even with *very small quantities* of labeled target data. As shown in Figure 5, good initial performance was achieved with data from just 1-5 patients or 5-25 minutes of signals. Similarly, for updates (Figure 8), performance improved significantly with as few as k=3 to k=20 new shots per class. Furthermore, the experiment explained in the reviewer's Question 1 showed that a traditional ML method trained on 90 minutes of annotated signal could not achieve the performance of MetaWearS used 5 minutes of annotated data. we agree with the reviewer that the annotation is a time- (and cost-) consuming process. Therefore, we propose MetaWearS to decrease the necessary amount of annotated data.

(c) **Real-time Operation and Clinician-in-the-loop:** It's crucial to distinguish between the *inference* process and the *model update cycle*.

- **Inference (Detection):** MetaWearS runs in **real-time** on the wearable device for detecting abnormalities. Our hardware evaluation (Section 2.4, Table 1) shows that the processing time per window of data (e.g., 0.76s for AF, 1.9s for Epilepsy in low-latency mode) is well within the real-time requirements (10s / 12s windows). The deployed model continuously analyzes incoming signals without needing immediate clinician input.
- **Update Cycle:** The process of *updating* the model does indeed rely on annotations that are typically generated offline by clinicians, which can be time-consuming. As the reviewer notes, new data is collected, potentially sent to a server, annotated by a clinician, and only then used to compute updated prototypes (as discussed in Section 4.4). These updated prototypes are then efficiently transmitted back to the device. Thanks to MetaWearS, this **update computation and transmission** are fast (as shown in Section 2.4). This offline annotation step does not hinder the real-time **operation** of the current model on the device; it only affects the frequency at which the model can be improved with new validated data. A clinician is 'in the loop' for providing the ground truth for updates, but not for the real-time inference itself.

Manuscript Modification: To explicitly clarify the distinction between real-time inference and the offline nature of the annotation-dependent update cycle, we have added a sentence at the end of Section 4.4 (Methods section discussing updates). The added text is highlighted below:

It is important to note that while the inference process (Algorithm 2) using the deployed prototypes runs in real-time on the wearable device (Section 2.4), the update cycle involving the acquisition and annotation of new samples ($\mathcal{D}^{new}$) typically includes an offline component [80-85]. This process depends on clinician availability and it is performed before the updated prototypes can be computed and transmitted.

References: [80] Lane, N. D., & Georgiev, P. (2015). Can deep learning revolutionize mobile sensing? Proceedings of the 16th International Workshop on Mobile Computing Systems and Applications, 117–122.

[81] Lane, N. D., Georgiev, P., & Qendro, L. (2015). DeepEar: Robust smartphone audio sensing in unconstrained acoustic environments using deep learning. Proceedings of the 2015 ACM International Joint Conference on Pervasive and Ubiquitous Computing, 283–294.

[82] McMahan, B., Moore, E., Ramage, D., Hampson, S., & y Arcas, B. A. (2017). Communication-efficient learning of deep networks from decentralized data. Proceedings of the 20th International Conference on Artificial Intelligence and Statistics, PMLR 54, 1273–1282.

[83] Ravi, D., Wong, C., Lo, B., & Yang, G.-Z. (2016). A deep learning approach to on-node sensor data analytics for mobile or wearable devices. IEEE Journal of Biomedical and Health Informatics, 21(1), 56–64.

[84] Shi, W., Cao, J., Zhang, Q., Li, Y., & Xu, L. (2016). Edge computing: Vision and challenges. IEEE Internet of Things Journal, 3(5), 637–646.

[85] Wang, X., Han, Y., Leung, V. C. M., Niyato, D., Yan, X., & Chen, X. (2020). Convergence of edge computing and deep learning: A comprehensive survey. IEEE Communications Surveys & Tutorials, 22(2), 869–904.

We hope this detailed explanation addresses the reviewer's concerns.

4. MetaWears updates prototype instead of weights to save time and battery. What about computational complexity, will it affect modelling time and battery?

We thank the reviewer for this insightful question. The process of calculating updated prototypes in MetaWearS, as described in Section 4.4, involves two main computational steps, typically performed server-side:

(a) Performing a forward pass of the few new annotated shots ($\mathcal{D}^{new}$) through the frozen feature extractor network f_ϕ to obtain their embeddings $f_\phi(x)$.

(b) Averaging the embeddings of the samples belonging to each class within the newly constructed support set S^n (using Eq. 3 and Eq. 1) to compute the updated prototypes c_n.

This process is computationally much lighter than retraining or fully fine-tuning the network weights, which requires computationally intensive backpropagation steps.

New Analysis Description: To provide quantitative evidence, we performed a new analysis (Experiment #4 in our planning). We compared the prototype update calculation (steps 1 and 2 above) against one epoch of stochastic gradient decent (SGD) fine-tuning (forward pass + loss + backward pass + weight update) for a single input sample. The comparison is based on computational complexity measured in Multiply-Accumulate operations (MACs).

Results Summary: Our analysis shows that the total computational cost of a full fine-tuning SGD step is approximately three times higher than our MetaWearS prototype update. For the AF Detection model, a full SGD update cycle requires 60.58 MMACs, compared to just 20.17 MMACs for the MetaWearS update. We observed the same threefold efficiency gain for the Seizure Detection model, which requires 9.61 MMACs for SGD fine-tuning versus 3.20 MMACs for our method. This significant reduction in computational demand is achieved by design, as MetaWearS completely avoids the expensive backpropagation and weight update stages inherent to model training, making our approach significantly faster and more energy-efficient.

Impact on Modeling Time and Battery: The reduced computational complexity translates to faster update calculations. Since the prototype calculation happens off-device, its computational cost (MACs) has a **negligible direct impact** on the wearable's battery life or real-time inference performance. The primary battery life benefit, as discussed extensively in Section 2.4, stems from the

dramatically reduced energy needed for **wireless transmission** of the compact prototypes compared to large model weights.

Manuscript Modification: We have added a brief clarification emphasizing the low computational cost of the prototype update calculation itself in the Methods section (Section 4.4). The added text is highlighted below:

> Furthermore, the computational process of updating prototypes (requiring only forward passes of new shots and embedding averaging) is significantly less complex and faster than conventional weight fine-tuning via backpropagation. Our analysis indicates that a full fine-tuning step is approximately three times more computationally expensive than our prototype-based update. For our AF Detection model, a complete fine-tuning cycle–encompassing a forward pass, loss calculation, backward pass, and weight update–requires 60.58 MMACs, whereas our method requires only 20.17 MMACs. This threefold efficiency gain is consistent across different architectures, as demonstrated by our Seizure Detection model, which requires 9.61 MMACs for fine-tuning versus 3.20 MMACs for the MetaWearS update. This substantial reduction in computational overhead directly translates to faster model adaptation and lower energy consumption, which are critical for on-device learning scenarios.

We hope this detailed analysis thoroughly addresses the reviewer's question.

5. Here are some related references in few-shot and meta learning in healthcare:
   - Few-shot learning for cardiac arrhythmia detection based on electrocardiogram data from wearable devices
   - Personalized blood glucose prediction for type 1 diabetes using evidential deep learning and meta-learning
   - Few-shot transfer learning for wearable IMU-based human activity recognition

We thank the reviewer for suggesting these highly relevant references. We have reviewed these valuable papers and incorporated citations and discussion within the revised Introduction (Section 1) and Discussion (Section 3) sections to better contextualize MetaWearS.

While all these papers address important aspects of few-shot learning in healthcare (related to our Challenge ① - minimal initial data, and Challenge ② - adaptation/update from few samples), their primary focus differs from the holistic lifecycle approach of MetaWearS. The suggested works primarily concentrate on achieving high performance in the initial few-shot learning or personalization phase (Challenge ①/②), leveraging techniques like meta-transfer learning or optimization-based meta-learning (MAML, Reptile). MetaWearS uniquely provides an integrated solution for **all three** lifecycle challenges, demonstrating strong few-shot performance with minimal initial data (Challenge ①) while also incorporating an efficient prototype-based update mechanism that minimizes both subsequent data requirements (Challenge ②) and energy consumption (Challenge ③) during deployment. While the referenced studies address aspects primarily related to Challenge ①, the holistic focus on the full lifecycle distinguishes MetaWearS.

Prompted by the reviewer's suggestion to consider these related works, and noting that the glucose prediction paper utilizes MAML (a prominent optimization-based method), we recognized the value of a direct comparison. Therefore, in addition to citing these works, we performed a **new experiment** (Experiment #2 in our planning) to benchmark MetaWearS (a metric-based approach) against MAML. However, given the suboptimal performance of MAML on the AF detection task, we expanded our benchmarking to include two prominent metric-based methods: the Matching Network and Relation Network.

The core distinction of MetaWearS lies not in achieving superior classification accuracy alone, but in being architected for the unique lifecycle constraints of wearable systems. In particular, in addition to the initial training data (Challenge ①), it also addresses the data requirements for subsequent model updates (Challenge ②) and, most critically, the energy efficiency of the update protocol itself (Challenge ③). Established methods like MAML, while powerful, were designed for general few-shot

accuracy and do not address these crucial lifecycle considerations for resource-constrained wearable devices.

To directly investigate the trade-offs, we performed a new comparative analysis against MAML, Matching Networks, and Relation Networks. The goal of this experiment was to answer a critical question: *Can MetaWearS deliver its lifecycle efficiency benefits without compromising the few-shot classification performance established by these state-of-the-art methods?*

New Experiment Summary & Findings: We implemented MAML using the same feature extractor backbones (Vision Transformer for epilepsy, and MobileNetV2-inspired CNN for AF) as MetaWearS. The learning difference between the two methods immediately highlights the superiority of our MetaWearS Prototypical Network-based approach for the wearable lifecycle (Challenges ② & ③). MAML adapts by learning an initialization for gradient-based fine-tuning of the model's weights. During deployment, any update would require further computationally-intensive gradient steps and the wireless transmission of the entire set of updated weights. MetaWearS, in stark contrast, performs updates by simply recalculating the class prototypes from new data shots and transmitting only these extremely compact vectors. This distinction is the source of our method's primary advantage: as detailed in our hardware analysis, it leads to **orders-of-magnitude reduction (418x-456x)** in the energy consumption required for an update.

Similar to the methodology used in Section 2.2.2 to compare our work with the source-only baseline, both MetaWearS and MAML methods were pre-trained on the respective base datasets (TUSZ EEG, PhysioNet ECG) using their standard meta-training procedures. We then evaluated their performance on the few-shot fine-tuning task using the target datasets (Siena EEG, private wearable ECG). For the seizure detection task, we obtained that the classification performance of MetaWearS is not statistically different with MAML ($p > 0.05$), and MetaWearS performs better with an average of 0.9% of AUC. This demonstrates that our efficiency gains come with no loss in accuracy in this scenario. Furthermore, for the more challenging AF detection task, which involves a greater domain shift between the base and target datasets, MetaWearS demonstrated a statistically significant **improvement** in performance over MAML, with an average AUC improvement of 6.4% ($p<0.05$).

These results empirically confirm that our prototype-based approach not only enables the immense energy savings critical for the wearable lifecycle but does so while maintaining a competitive, and in some cases superior, classification accuracy.

This fundamental advantage in lifecycle efficiency extends beyond optimization-based methods and holds true when comparing MetaWearS to other prominent metric-based approaches, namely Matching Networks and Relation Networks. More importantly, these alternative architectures share a critical limitation regarding lifecycle efficiency (Challenges ② and ③). Unlike MetaWearS, which condenses all support set information into a single prototype, both Matching and Relation Networks require the feature vectors of *every individual sample* in the support set to perform a classification. Consequently, to update the system with k new shots, all k feature vectors must be transmitted, a process that is k times less efficient than the single prototype update of MetaWearS.

While establishing this key efficiency advantage, our analysis also confirmed that it does not come at the expense of accuracy. In case of AF detection, MetaWearS demonstrated competitive performance, significantly outperforming Matching Networks with a mean AUC improvement of 2.8% ($p < 0.05$) and achieving an accuracy similar with the highly competitive Relation Network, with a mean improvement of 1.7% ($p > 0.05$). This unique combination of high performance and an architecturally superior, energy-efficient update mechanism solidifies the value of the MetaWearS framework.

In summary, what differentiates MetaWearS is its unique prototype-based architecture, which is explicitly co-designed for the practical constraints of the wearable lifecycle. This design provides an ultra-lightweight and energy-efficient update mechanism—leading to orders-of-magnitude energy savings—while our new experiments confirm that it simultaneously maintains a classification accuracy that is on-par with, or superior to, established meta-learning benchmarks like MAML, Matching Networks, and Relation Networks.

Manuscript Addition: We have added a new subsection (Section 3.1) to the manuscript to detail this comparative analysis and integrate these findings. The added text is provided below.

Comparison with other Few-Shot Learning Methods

To contextualize the performance of MetaWearS, we benchmarked it against several established few-shot learning algorithms, including MAML, Matching Networks, and Relation Networks. Our analysis confirms that MetaWearS provides its significant lifecycle efficiency benefits without compromising on classification performance.

Comparison with MAML. The primary distinction lies in the update mechanism, which directly impacts the wearable lifecycle (Challenges ② & ③). MAML adapts by fine-tuning its model weights via gradient descent, requiring the wireless transmission of the entire updated model for on-device deployment. In stark contrast, MetaWearS leverages its Prototypical Network foundation to perform updates by simply recalculating and transmitting a single, compact prototype vector. This architectural difference leads to an orders-of-magnitude reduction (418x-456x) in the energy required for model updates.

Having established this efficiency advantage, for the performance comparison, we followed a methodology similar to the one used in Section 2.2.2. All methods were first pre-trained on their respective base datasets (TUSZ EEG, PhysioNet ECG) using their standard meta-training procedures. Subsequently, we evaluated their few-shot classification performance on the corresponding target datasets (Siena EEG, private wearable ECG).

Our experiments confirm that the MetaWearS advantage on update efficiency does not come at the cost of performance. For the seizure detection task, the classification performance of MetaWearS was not statistically different with MAML ($p > 0.05$), and MetaWearS has a mean AUC improvement of 0.9%. For the more challenging AF detection task, which involves a greater domain shift, MetaWearS demonstrated a statistically significant **improvement** over MAML, with an average AUC improvement of 6.4% ($p < 0.05$). This performance gap can be attributed to MAML's known vulnerability to overfitting on small support sets, whereas the implicit regularization of our prototype-based approach ensures greater robustness.

Comparison with other Metric-Based Methods. This fundamental advantage in lifecycle efficiency extends to other prominent metric-based approaches, namely Matching Networks and Relation Networks. These architectures share a critical limitation: unlike MetaWearS, which condenses all support set information into a single prototype, they require the feature vectors of *every individual sample* in the support set for classification. To update the system with k new shots, all k feature vectors must be transmitted, a process that is k times less efficient than the single prototype update of MetaWearS.

While being architecturally more efficient, MetaWearS also demonstrated competitive accuracy. In the case of AF detection, it significantly outperformed Matching Networks with a mean AUC improvement of 2.8% ($p < 0.05$) and achieved an accuracy similar to the highly competitive Relation Network, with a mean improvement of 1.7% ($p > 0.05$). This unique combination of validated high performance and an architecturally superior, energy-efficient update mechanism solidifies the value of the MetaWearS framework for practical wearable systems.

We believe incorporating these references and performing the suggested comparison significantly strengthens the paper.

6. The discussion and ablation study were presented well. The methods include lots of necessary details. Well done.

We thank the reviewer for their positive feedback on mentioned sections. We appreciate the acknowledgment of the clarity and detail provided.

Reviewer #3

The paper introduces MetaWearS, a meta-learning framework designed to address key challenges in wearable health monitoring systems: limited labelled data, inefficient model updates, and high energy consumption. By leveraging Prototypical Networks for few-shot learning, MetaWearS enables effective detection of biomedical abnormalities (e.g., in two key examples, such as epilepsy and atrial fibrillation) with minimal initial training data.

Strengths:

- The paper is very relevant in wearables and low-power embedded systems. The objectives and contributions are very well stated and significant. It addresses real-world challenges.
- I appreciate the idea of using meta-learning as a way to reduce the dependency on large labelled datasets, which I agree with authors, in the field of healthcare, are challenging to find, particularly in EEG.
- The use of a RISC-V-based system for implementation adds a practical contribution that makes the deployability of the method proposed more credible.
- The results obtained are promising.
- The paper is clear and well-written.

We greatly appreciate the reviewer acknowledging the strengths of our paper.

Weaknesses:

- Although the authors demonstrate the method's applicability across two distinct biomedical signal types, the paper does not dissipate doubt about the method's generalization ability. Can authors better discuss this aspect? For example, in EEG, several kinds of analysis are more affected, if possible, by the variability of data and the need to calibrate the data on different patients. How does this method generalize in these cases?

  We thank the reviewer for raising this important point regarding the generalization ability of MetaWearS. The MetaWearS framework is indeed designed with generalization to new, unseen patients as a core principle, addressed through several mechanisms:

  - **Meta-Training Strategy:** During the meta-training phase (both pre-training on the base dataset and few-shot fine-tuning on the target dataset, detailed in Section 4.3), we employ an episodic training approach. As described (see Fig. 2 and Fig. 3), each episode's support set and query set are constructed using data from *different, non-overlapping patients.* This forces the model to learn to generalize from a few examples of one group of patients to new, unseen patients within the query set.
  - **Fine-tuning on Target Domain:** The fine-tuning step on the target dataset adapts the model learned on the base dataset to the specific characteristics and variability inherent in the target user population and wearable device data (Section 4.3). Our experiments (Section 2.2.1, Fig. 5) evaluate performance on test patients not seen during this fine-tuning support set construction.
  - **Prototype Update Mechanism for Personalization:** For ongoing adaptation (Challenge ②), the prototype update mechanism (Section 2.3, Fig. 8 & 9) is specifically designed to calibrate the model to new end-users by incorporating a few of their labeled samples. This allows the prototypes to be personalized over time.

  While these mechanisms are designed to promote generalization, we acknowledge that the degree of variability and domain shift between the base dataset, the initial target dataset, and new end-user data will influence performance. High inter-patient variability, as often seen in EEG, indeed poses a significant challenge.

  Manuscript Modification: To better discuss this aspect, we have added a dedicated paragraph in the **Discussion section** (Section 3.5). This paragraph elaborates on how MetaWearS addresses generalization and acknowledges current limitations. The added text is highlighted below:

A key consideration in wearable health monitoring is the model's ability to generalize to new, unseen patients, particularly given the high inter-patient variability common in biomedical signals. MetaWearS is designed to inherently address this through its meta-learning paradigm. The episodic training during both pre-training and fine-tuning (Section 4.3) explicitly trains the model to make predictions for patients not seen in the support set of a given episode. Furthermore, the fine-tuning process itself adapts the model to the target population, and the prototype update mechanism allows for ongoing personalization as data from new users become available. While these strategies aim to enhance generalization, the extent of domain shift between the base data, initial target data, and new user data remains a factor influencing performance. Future work could explore the robustness against severe data variability and improve calibration for highly diverse patient cohorts.

We believe this addition provides a more thorough discussion of the generalization aspect.

- Is the choice of 3 quantities per case done in section 2.2.1 sufficient? How have the 5,15,25 for AF and 1,3,5 for epilepsy been chosen? What is the medical reference? Can the authors justify better or expand?

  We thank the reviewer for this question.

  The primary rationale for selecting these specific small quantities (1, 3, and 5 patients for epilepsy; 5, 15, and 25 minutes of signals for AF) was to empirically demonstrate the core capability of MetaWearS: its ability to achieve meaningful performance with **extremely limited initial labeled data from the target domain** (Challenge ①). Our goal was to test the few-shot learning aspect of our framework by exploring the lower bounds of data requirements.

  Regarding a specific "medical reference" for these exact quantities for model training: these values were chosen primarily to represent challenging yet plausible data scarcity scenarios in the initial deployment of wearable systems. Obtaining extensive annotated data from many users is often a major issue. While the chosen small quantities serve as critical set-points to validate our few-shot learning claims, we agree that a more explicit quantification of this benefit strengthens our manuscript. Therefore, to directly demonstrate and measure the extent of the data reduction achieved by MetaWearS, we conducted a new head-to-head comparison against a traditional supervised learning baseline. In this analysis, we aimed to determine the amount of labeled target data the baseline model would require to approach the high performance MetaWearS achieves with only a minimal amount of signal.

  New Experiment Description: We compared MetaWearS with a standard supervised learning baseline (trained **only** on the target dataset with traditional ML methods). We incrementally increased the amount of labeled data provided to the supervised baseline and measured performance (AUC). We determined the amount of target data that the baseline required to achieve a performance level comparable to MetaWearS performance. The test set, comprising 20% of the target data set, remained consistent in all experiments to ensure a fair comparison.

  Results Summary: For the AF detection task, we conducted this experiment using two architectures with the supervised baseline.

  In the first baseline experiment, we utilized the same architecture as MetaWearS. Due to the scarcity of initial data in the target dataset, this model shows significant overfitting even with a gradual increase in data size. This phenomenon prevents the baseline model from achieving satisfactory performance.

  For the second experiment, to enable a more meaningful comparison and achieve a comparable performance trajectory, we employed a more compact MobileNet-style architecture with a lower number of parameters than MetaWearS. As mentioned in the manuscript, this architecture was used in [38]. The results of training this model solely on the target dataset are presented in Figure 3. The x-axis represents the minutes of labeled signal from the target dataset used for training/fine-tuning, and the y-axis represents the Area Under the Curve (AUC) performance.

  As illustrated in Figure 3, MetaWearS achieves an AUC of 89% with only 5 minutes of the labeled ECG signal from the target dataset. In stark contrast, the supervised baseline model, with its reduced complexity, only began to show improvement at 50 minutes of signal, reaching 68.2% AUC. Furthermore,

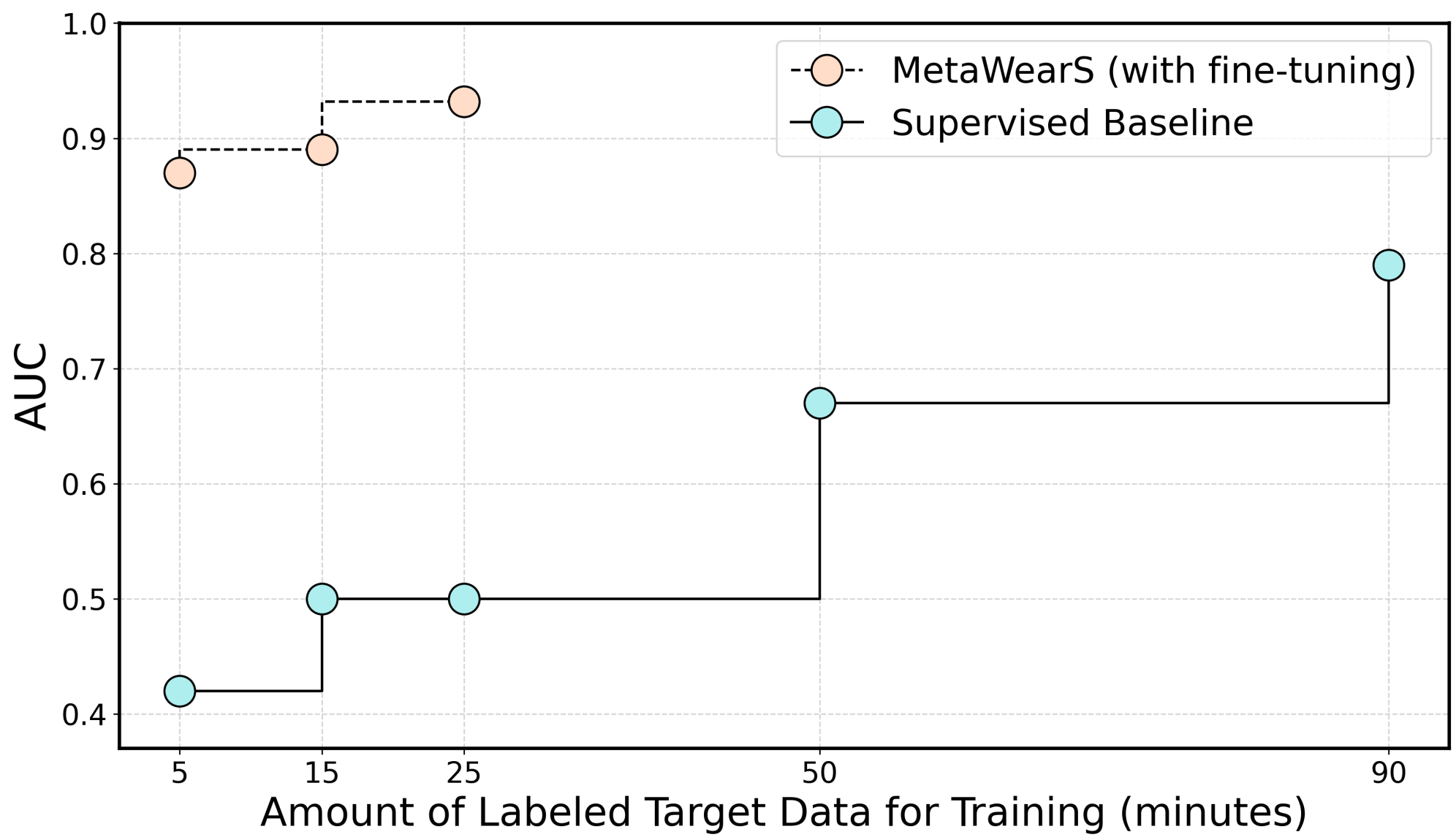

Figure 3: Initial data increase in AF

it could not achieve an AUC of 80% even with 90 minutes of training data. This clearly demonstrates that MetaWearS significantly reduces the amount of initial labeled data required to achieve high performance.

Manuscript Addition: Based on this experiment, we have added a new subsection detailing this analysis and its results. We added this as Subsection 2.2.3, titled "Quantifying initial data reduction", following the initial results presentation. The added text is highlighted below:

> **2.2.3 Comparison with Target-Only Supervised Baseline:** To explicitly quantify the benefit of MetaWearS in reducing the need for target-specific labeled data (Challenge ①), we compared its performance against a Target-Only Supervised Baseline. This baseline model was trained from scratch using only the labeled data from the target dataset. We incrementally increased the amount of training data available to the baseline, from a few minutes up to 90 minutes of signal, to observe the performance trajectory. We first attempted to use a model with the same architecture as MetaWearS for the baseline; however, due to the severe scarcity of initial data, this model showed significant overfitting and failed to achieve a satisfactory performance level. To facilitate a more meaningful comparison, we then employed a more compact MobileNet-style architecture with fewer parameters for the baseline, as used in [38], which is better suited for training on smaller datasets. The test set was kept consistent across all experiments to ensure a fair evaluation.
>
> The results for the AF detection task are presented in Fig. 7. The figure plots AUC against the minutes of labeled signal used for training. As illustrated, MetaWearS achieves a high performance of 89% AUC using only 5 minutes of the labeled ECG signal from the target user. In stark contrast, the supervised baseline requires significantly more data to even begin learning effectively. It only starts to show a significant improvement after 50 minutes of signal, reaching an AUC of 68.2%. Even when provided 90 minutes of training data, the baseline performance remains below the 80% AUC, failing to reach the level achieved by MetaWearS with a fraction of the data.
>
> This result clearly quantifies the data-efficiency of our approach. MetaWearS can achieve high accuracy with a lower amount of initial data, whereas a traditional approach would require a substantially larger, and in this case prohibitively large, dataset to approach comparable performance. This directly validates our claim that MetaWearS effectively addresses the challenge of reducing the initial data burden for personalizing wearable sensor models.

References: [38] Kim, S., Chon, S., Kim, J.-K., Kim, J., Gil, Y. and Jung, S. Lightweight Convolutional Neural Network for Real-Time Arrhythmia Classification on Low-Power Wearable Electrocardiograph in 44th Annual International Conference of the IEEE Engineering in Medicine and Biology Society (EMBC) (2022), 1915–1918.

In addition to incorporating this new analysis as a dedicated subsection (2.2.3), we have also revised the original results section to explicitly state the rationale for choosing these small data quantities. This edit addresses the core of the reviewer's question directly within the manuscript, ensuring our reasoning is clear to all readers. The specific sentence added to Section 2.2.1 is shown below.

Manuscript Modification: To better clarify this rationale within the manuscript, we have added a sentence to Section 2.2.1 ("Quantitative results"). The added text is highlighted below:

> These specific small quantities were chosen to rigorously evaluate the few-shot learning capability of MetaWearS under conditions of extreme data scarcity, representative of challenges in initial wearable system deployment, and to provide clear data points for illustrating the performance trend with empirically minimal samples in Figure 5.

We trust this explanation clarifies our experimental design choices for demonstrating few-shot learning performance.

- What happens in cases with more complex signals, e.g. multi-channel or multimodal signals? What happens in the case of non-binary classifications? Please comment on the scalability of the method.

  We thank the reviewer for this insightful question regarding the scalability of MetaWearS to more complex signal types and classification tasks.

  Non-binary Classifications: The MetaWearS framework, which is based on Prototypical Networks, inherently supports non-binary (multi-class) classification. As described in Section 2.1 and Equation 1 of our manuscript, a prototype c_n is computed for each class n present in the support set. Classification of a query sample is then performed by assigning it to the class of the nearest prototype in the embedding space. While our case studies focused on binary classification (normal vs. abnormal) for clarity and based on the available annotated target data, the underlying mechanism readily extends to scenarios with more than two classes. In fact, our ECG model's architecture, as detailed in Section 4.2.2, initially included a final layer designed for four ECG classes before we adapted it for the binary target task.

  Multi-channel Signals: MetaWearS already demonstrates its applicability to multi-channel signals in our epileptic seizure detection case study (Section 4.1). We utilize multi-channel EEG recordings, and the preprocessing involves creating STFT "images" from these signals (Section 4.1.1). These STFTs inherently capture information from the multiple EEG channels, which are then processed by the Vision Transformer. The framework is thus directly applicable to multi-channel inputs, provided the feature extractor f_ϕ is designed to handle such data.

  Multi-modal Signals: Extending MetaWearS to multi-modal signals (e.g., combining ECG with accelerometer data) would primarily involve designing a more complex feature extractor network f_ϕ. This network would need to be capable of processing each modality appropriately and then fusing the information to produce a unified embedding vector. Fusion could be achieved through various strategies, such as concatenation of features from modality-specific branches, attention mechanisms, or other sensor fusion techniques, before the final embedding layer from which prototypes are computed. The core meta-learning principles of MetaWearS—episodic training, prototype calculation, and efficient prototype-only updates—would remain applicable to this joint embedding space. The main scalability consideration for multi-modal data would be the increased complexity and resource requirements of the feature extractor f_ϕ, which would impact training time and potentially the on-device inference cost. However, the update mechanism via compact prototypes would retain its efficiency benefits.

  Manuscript Modification: To explicitly address these scalability aspects, we have added a new subsection to the **Discussion section** (Section 3.3.3). The added text is highlighted below:

  > Regarding the scalability of MetaWearS, the framework is inherently capable of handling non-binary classification tasks, as Prototypical Networks compute a distinct prototype for

each class. Our epileptic seizure case study already demonstrates its use with multi-channel EEG data. Extension to multi-modal signals would primarily necessitate the design of a suitable feature extractor f_ϕ capable of processing and fusing diverse data streams into a coherent embedding space, from which prototypes can then be derived. While this might increase the complexity of f_ϕ, the core meta-learning and efficient prototype-update mechanisms of MetaWearS remain applicable, offering a scalable approach for various complex wearable sensing scenarios.

We believe this clarifies the scalability of MetaWearS for more complex scenarios.

- Is the choice of Prototypical Networks the only possible? What about other methods like Relation Networks or Matching Networks?

  We thank the reviewer for this question regarding our choice of Prototypical Networks as the underlying few-shot learning mechanism for MetaWearS.

  Prototypical Networks [14] were chosen for several key reasons that align well with the objectives of MetaWearS, particularly in the context of wearable systems:

  - **Simplicity and Efficiency:** Prototypical Networks offer a conceptually simple and computationally efficient approach. Calculating prototypes as the mean of support sample embeddings in the learned feature space is straightforward. This efficiency is beneficial both for the initial few-shot classification and, critically, for our proposed lightweight update mechanism.
  - **Effectiveness in Few-Shot Learning:** They have been shown to be highly effective across various few-shot learning benchmarks, providing a strong foundation for learning from limited data.
  - **Direct Suitability for Prototype-Only Updates:** The core concept of class prototypes aligns perfectly with our strategy for addressing Challenge ③ (energy-efficient updates). The ability to update the model's knowledge by merely recalculating and transmitting these compact prototype vectors was a primary motivator for their adoption, as this directly leads to significant energy and bandwidth savings.

  We acknowledge that other metric-based meta-learning methods, such as Matching Networks or Relation Networks, also exist and have demonstrated success in few-shot learning. These methods could potentially be adapted for our framework.

  New Experiment: To provide a broader comparison against a different class of meta-learning algorithms (optimization-based), we conducted a new experiment (Experiment #2 in our planning) benchmarking MetaWearS against Model-Agnostic Meta-Learning (MAML), Relation Network, and Matching Network.

  The core distinction of MetaWearS lies not in achieving superior classification accuracy alone, but in being architected for the unique lifecycle constraints of wearable systems. In particular, in addition to the initial training data (Challenge ①), it also addresses the data requirements for subsequent model updates (Challenge ②) and, most critically, the energy efficiency of the update protocol itself (Challenge ③). Established methods like MAML, while powerful, were designed for general few-shot accuracy and do not address these crucial lifecycle considerations for resource-constrained wearable devices.

  To directly investigate the trade-offs, we performed a new comparative analysis against MAML, Matching Networks, and Relation Networks. The goal of this experiment was to answer a critical question: *Can MetaWearS deliver its lifecycle efficiency benefits without compromising the few-shot classification performance established by these state-of-the-art methods?*

  New Experiment Summary & Findings: We implemented MAML using the same feature extractor backbones (Vision Transformer for epilepsy, and MobileNetV2-inspired CNN for AF) as MetaWearS. The learning difference between the two methods immediately highlights the superiority of our MetaWearS Prototypical Network-based approach for the wearable lifecycle (Challenges ② & ③). MAML adapts by learning an initialization for gradient-based fine-tuning of the model's weights. During deployment,

any update would require further computationally-intensive gradient steps and the wireless transmission of the entire set of updated weights. MetaWearS, in stark contrast, performs updates by simply recalculating the class prototypes from new data shots and transmitting only these extremely compact vectors. This distinction is the source of our method's primary advantage: as detailed in our hardware analysis, it leads to **orders-of-magnitude reduction (418x-456x)** in the energy consumption required for an update.

Similar to the methodology used in Section 2.2.2 to compare our work with the source-only baseline, both MetaWearS and MAML methods were pre-trained on the respective base datasets (TUSZ EEG, PhysioNet ECG) using their standard meta-training procedures. We then evaluated their performance on the few-shot fine-tuning task using the target datasets (Siena EEG, private wearable ECG). For the seizure detection task, we obtained that the classification performance of MetaWearS is not statistically different with MAML ($p > 0.05$), and MetaWearS performs better with an average of 0.9% of AUC. This demonstrates that our efficiency gains come with no loss in accuracy in this scenario. Furthermore, for the more challenging AF detection task, which involves a greater domain shift between the base and target datasets, MetaWearS demonstrated a statistically significant **improvement** in performance over MAML, with an average AUC improvement of 6.4% ($p<0.05$).

These results empirically confirm that our prototype-based approach not only enables the immense energy savings critical for the wearable lifecycle but does so while maintaining a competitive, and in some cases superior, classification accuracy.

This fundamental advantage in lifecycle efficiency extends beyond optimization-based methods and holds true when comparing MetaWearS to other prominent metric-based approaches, namely Matching Networks and Relation Networks. More importantly, these alternative architectures share a critical limitation regarding lifecycle efficiency (Challenges ② and ③). Unlike MetaWearS, which condenses all support set information into a single prototype, both Matching and Relation Networks require the feature vectors of *every individual sample* in the support set to perform a classification. Consequently, to update the system with k new shots, all k feature vectors must be transmitted, a process that is k times less efficient than the single prototype update of MetaWearS.

While establishing this key efficiency advantage, our analysis also confirmed that it does not come at the expense of accuracy. In case of AF detection, MetaWearS demonstrated competitive performance, significantly outperforming Matching Networks with a mean AUC improvement of 2.8% ($p < 0.05$) and achieving an accuracy similar with the highly competitive Relation Network, with a mean improvement of 1.7% ($p > 0.05$). This unique combination of high performance and an architecturally superior, energy-efficient update mechanism solidifies the value of the MetaWearS framework.

In summary, what differentiates MetaWearS is its unique prototype-based architecture, which is explicitly co-designed for the practical constraints of the wearable lifecycle. This design provides an ultra-lightweight and energy-efficient update mechanism—leading to orders-of-magnitude energy savings—while our new experiments confirm that it simultaneously maintains a classification accuracy that is on-par with, or superior to, established meta-learning benchmarks like MAML, Matching Networks, and Relation Networks.

Manuscript Addition: We have added a new subsection (Section 3.1) to the manuscript to detail this comparative analysis and integrate these findings. The added text is provided below.

> **Comparison with other Few-Shot Learning Methods**
>
> To contextualize the performance of MetaWearS, we benchmarked it against several established few-shot learning algorithms, including MAML, Matching Networks, and Relation Networks. Our analysis confirms that MetaWearS provides its significant lifecycle efficiency benefits without compromising on classification performance.
>
> **Comparison with MAML.** The primary distinction lies in the update mechanism, which directly impacts the wearable lifecycle (Challenges ② & ③). MAML adapts by fine-tuning its model weights via gradient descent, requiring the wireless transmission of the entire updated model for on-device deployment. In stark contrast, MetaWearS leverages its Prototypical Network foundation to perform updates by simply recalculating and transmitting a

single, compact prototype vector. This architectural difference leads to an orders-of-magnitude reduction (418x-456x) in the energy required for model updates.

Having established this efficiency advantage, for the performance comparison, we followed a methodology similar to the one used in Section 2.2.2. All methods were first pre-trained on their respective base datasets (TUSZ EEG, PhysioNet ECG) using their standard meta-training procedures. Subsequently, we evaluated their few-shot classification performance on the corresponding target datasets (Siena EEG, private wearable ECG).

Our experiments confirm that the MetaWearS advantage on update efficiency does not come at the cost of performance. For the seizure detection task, the classification performance of MetaWearS was not statistically different with MAML ($p > 0.05$), and MetaWearS has a mean AUC improvement of 0.9%. For the more challenging AF detection task, which involves a greater domain shift, MetaWearS demonstrated a statistically significant **improvement** over MAML, with an average AUC improvement of 6.4% ($p < 0.05$). This performance gap can be attributed to MAML's known vulnerability to overfitting on small support sets, whereas the implicit regularization of our prototype-based approach ensures greater robustness.

Comparison with other Metric-Based Methods. This fundamental advantage in lifecycle efficiency extends to other prominent metric-based approaches, namely Matching Networks and Relation Networks. These architectures share a critical limitation: unlike MetaWearS, which condenses all support set information into a single prototype, they require the feature vectors of *every individual sample* in the support set for classification. To update the system with k new shots, all k feature vectors must be transmitted, a process that is k times less efficient than the single prototype update of MetaWearS.

While being architecturally more efficient, MetaWearS also demonstrated competitive accuracy. In the case of AF detection, it significantly outperformed Matching Networks with a mean AUC improvement of 2.8% ($p < 0.05$) and achieved an accuracy similar to the highly competitive Relation Network, with a mean improvement of 1.7% ($p > 0.05$). This unique combination of validated high performance and an architecturally superior, energy-efficient update mechanism solidifies the value of the MetaWearS framework for practical wearable systems.

We hope this clarifies our reasoning and that the added MAML comparison provides useful context.

- The datasets used (e.g., TUH and PhysioNet) are mostly from controlled environments (e.g., hospitals). The authors also use data from a wearable device in one of the 2 cases. However, how much of the data used is pre-processed and clean? Have the authors considered a test on more "dirty" datasets closer to real-world settings? The authors honestly admit this limitation in section 3.3. Can authors anyway provide more details on the robustness of the approach in non-controlled environments?

  We thank the reviewer for this important question. We agree that this is a critical aspect for the practical deployment of wearable systems.

  As the reviewer notes, our base datasets (TUH and PhysioNet) are from hospital settings. For the target datasets, the Siena EEG dataset is also hospital-based (though from a different setting than TUH), while for AF detection, we used a private dataset acquired using a wearable device, which inherently includes some real-world signal characteristics. All datasets undergo standard preprocessing steps (e.g., filtering, resampling, normalization, as detailed in Sections 4.1.1 and 4.2.2) to mitigate baseline noise and artifacts and ensure consistency. However, we concur that these steps do not fully replicate the challenges of "dirty" data from uncontrolled environments. We acknowledged this limitation in Section 3.3.

  New Experiment (Robustness to Simulated Noise): To provide more quantitative insight into the robustness of MetaWearS, we performed a new experiment (Experiment #5 in our planning).

  Experiment Description: We took the test sets from our target domains (Siena EEG for epilepsy and the private wearable ECG for AF detection) *after* their standard preprocessing. We then artificially added various types and levels of realistic noise commonly encountered in wearable scenarios. For EEG,

this included simulated muscle artifacts (EMG) and baseline wander. For ECG, we added baseline wander and motion artifacts. We evaluated the performance (AUC) of the fine-tuned MetaWearS models on these noise-corrupted test sets.

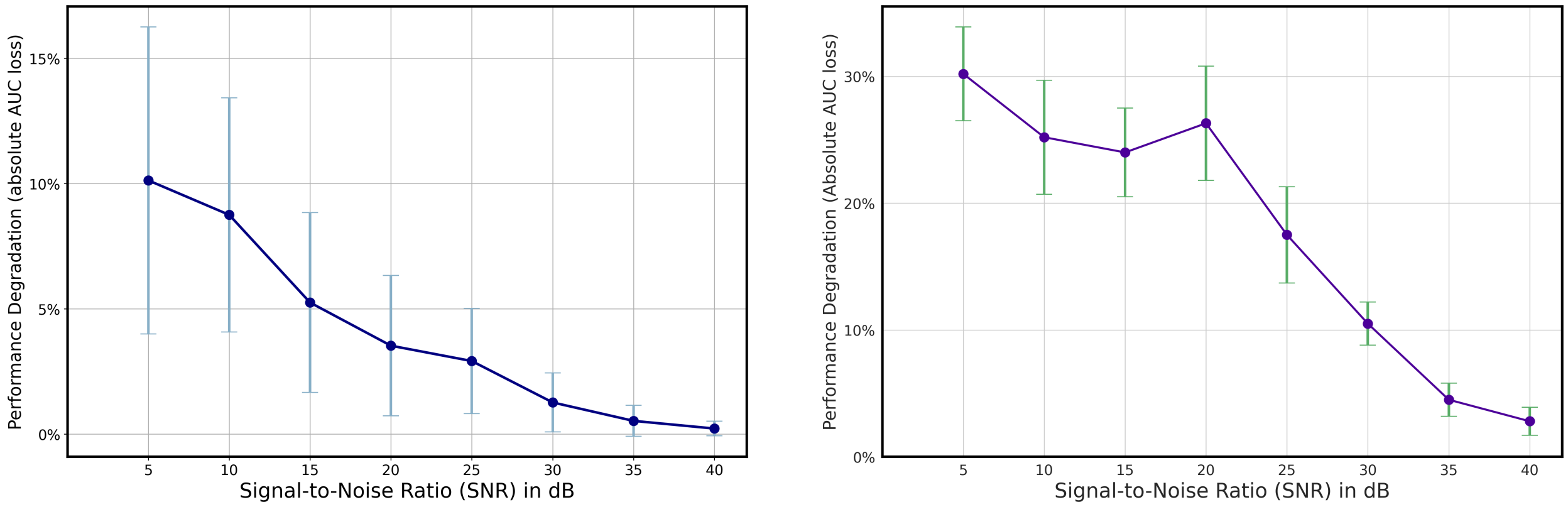

(a) epilepsy Noise Test

(b) AF Noise Test

Figure 4: Comparison of performance degradation across different SNR levels: (a) Epilepsy, (b) AF.

Results Summary Figures 4 demonstrate that MetaWearS's performance declines as noise increases, though with differing sensitivities for each task. At high-quality inputs (SNR $\geq$ 30 dB), the absolute AUC loss is small for both tasks, ranging from a few percent or less. Conversely, at low SNRs (5–10 dB), the loss becomes substantial, reaching approximately 25–30% for AF detection and up to 10% for the seizure detection task. The error bars from repeated noise realizations confirm these patterns are consistent. This indicates that while the model retains robust accuracy under low-to-moderate noise for epilepsy detection, its performance for AF detection is significantly more susceptible to severe noise contamination. Ultimately, for both tasks, the model demonstrates a strong ability to maintain high performance as signal quality improves, confirming its overall robustness.

Manuscript Addition: We have added a new subsection in the **Results section** (Section 3.3, titled "Robustness to Simulated Noise") detailing this experiment and its outcomes. The added text is highlighted and emphasized below:

3.3.1 Robustness to Simulated Noise *To assess the robustness of MetaWearS to common artifacts in non-controlled environments, we evaluated its performance on target test sets corrupted with simulated noise. After standard preprocessing, white noise was added at varying signal-to-noise ratios (SNRs).*

For the epilepsy detection task, where the model was fine-tuned on 5 patients (with a baseline AUC of 87.7%), the absolute AUC loss (Fig. 12a) was over 10% at a low 5 dB SNR. However, the model demonstrated strong resilience, with the loss decreasing to only 2.5-3% at a moderate 25 dB SNR, preserving a robust AUC of approximately 85.2-85.0%. Degradation became negligible at 40 dB SNR. Similarly, for AF detection, where the model was fine-tuned on 25 minutes of data (with a baseline AUC of 93.0%) and ECG signals were affected by motion artifacts and baseline wander, the absolute AUC loss (Fig. 12b) was approximately 30% at 5 dB SNR, reflecting significant interference. Yet, performance notably improved with higher SNRs, reducing to 17-17.5% at 25 dB SNR, corresponding to an AUC of about 75.5-76.0%. At 40 dB SNR, the loss was a minimal 2.5-3%. While performance degrades with increasing noise, these results indicate that MetaWearS maintains reasonable efficacy under common noise conditions, particularly when coupled with appropriate signal preprocessing.

- Strictly related to the previous comment. The discussion should also provide a more critical reflection on the limitations of the proposed approach, such as its dependency on the quality of the feature space, sensitivity to noise or challenges in adapting to highly imbalanced datasets.

We thank the reviewer for this constructive suggestion to enhance the critical reflection on the limitations of MetaWearS. We agree that these are important aspects to discuss.

Manuscript Modification: In response, we have significantly expanded the **"Study limitations and future perspective" subsection** (Section 3.3) in the Discussion to more thoroughly address the points raised. The revised section now includes a more critical reflection on:

- **Dependency on Feature Space Quality:** We now explicitly state that the performance of MetaWearS, akin to other metric-learning and prototype-based methods, fundamentally relies on the quality and discriminative power of the embedding space learned by the feature extractor (f_ϕ). If the feature extractor fails to produce embeddings that effectively separate the underlying classes, the efficacy of the prototype-based classification will be compromised.
- **Sensitivity to Noise:** Building on our response to the previous comment and the new "Robustness to Simulated Noise" experiment (Experiment #5, now detailed in the Discussion Section), we discuss that while standard preprocessing (Sections 4.1.1, 4.2.2) and the learned features provide a degree of resilience, MetaWearS's performance can be sensitive to high levels of noise or artifacts not adequately mitigated by these preprocessing steps. The new experimental results provide quantitative insights into this sensitivity.
- **Data Imbalance Handling:** Concerning data imbalance, a notable strength of MetaWearS's few-shot, episodic training design is its inherent robustness to class imbalance during the fine-tuning and update phases on the target device. Because we explicitly sample a small, fixed number of shots (k) per class for both support and query sets in each episode, the learning process itself is balanced on a per-episode basis. This mitigates concerns about the model being skewed by disproportionate class representation within the *target few-shot data*. The primary challenge related to imbalance in the target domain then becomes the practical one of sourcing at least k representative samples for each class, especially rare ones. Imbalance within the *base dataset*, which is used for pre-training, is a more traditional concern; for instance, we addressed this in the base AF dataset through random resampling and Gaussian noise augmentation (Section 4.2.1) to ensure the pre-trained model is not unduly biased before adaptation. Thus, MetaWearS's structure inherently manages imbalance at the crucial few-shot adaptation stage, provided a minimal number of representative samples per class can be obtained.

The expanded text in Section 3.5 incorporates these reflections. An exemplary excerpt of the additions is highlighted below:

> Furthermore, the efficacy of MetaWearS, like other approaches relying on learned embeddings, is inherently tied to the quality of the feature space generated by the encoder f_ϕ. A poorly discriminative feature space will inevitably limit the performance of the prototype-based classifier.

Furthermore, we have added the following discussion to Section 3.3.2:

> **Data Imbalance Handling:** Concerning data imbalance, a notable strength of MetaWearS's few-shot, episodic training design is its inherent robustness to class imbalance during the fine-tuning and update phases on the target device. Because we explicitly sample a small, fixed number of shots (k) per class for both support and query sets in each episode, the learning process itself is balanced on a per-episode basis. This mitigates concerns about the model being skewed by disproportionate class representation within the *target few-shot data*. The primary challenge related to imbalance in the target domain then becomes the practical one of sourcing at least k representative samples for each class, especially rare ones. Imbalance within the *base dataset*, which is used for pre-training, is a more traditional concern; for instance, we addressed this in the base AF dataset through random resampling and Gaussian noise augmentation to ensure the pre-trained model is not unduly biased before adaptation. Thus, MetaWearS's structure inherently manages imbalance at the crucial few-shot adaptation stage, provided a minimal number of representative samples per class can be obtained.

We believe these additions provide the more critical reflection requested by the reviewer.

- While the paper compares MetaWearS with a conventional approach, it doesn't benchmark against other state-of-the-art meta-learning techniques. A broader comparative analysis would strengthen the claims.

  We thank the reviewer for this suggestion. We agree that a comparative analysis with other state-of-the-art meta-learning techniques would indeed strengthen our claims.

  As detailed in our response to the reviewer's previous point (Weakness 4, regarding the choice of Prototypical Networks), we have performed a **new experiment** to provide such a comparison. Specifically, we benchmarked MetaWearS (which is based on metric learning via Prototypical Networks) against Model-Agnostic Meta-Learning (MAML), a prominent optimization-based SOTA meta-learning algorithm.

  The full description of this new MAML experiment, including the setup, results summary (performance for Challenge ①), and a discussion of the implications for Challenge ② (Updates) and Challenge ③ (Energy Efficiency), is provided in our response to Weakness 4 above.

  Manuscript Addition: As stated in that previous response, we have added a new subsection detailing this comparative analysis with MAML. This has been added as **Subsection 3.1, titled "Comparison with MAML"**, within the Discussion section of the revised manuscript.

  We believe this new experimental comparison with MAML directly addresses the reviewer's concern for a broader comparative analysis and better positions MetaWearS within the state-of-the-art.

- Although the paper emphasizes energy efficiency, it doesn't fully address the trade-offs involved in computational demands, memory usage, or latency for different hardware platforms. I confirm I appreciated the deployment of MetaWearS on the RISC-V hardware. However, as a minor shortcoming, a deployment (or an applicability evaluation) also in a more standard and easy-to-find platform would have increased the value of the demonstration.

  We thank the reviewer for this valuable suggestion to explore trade-offs and evaluate MetaWearS on more hardware platforms. We agree that this broadens the applicability and strengthens the demonstration of our approach.

  New Experiment (Deployment on Additional Hardware Platforms): To address this, we have performed a new set of experiments (Experiment #3 in our planning). In addition to the custom X-HEEP platform, we have now deployed and evaluated MetaWearS on two widely available ARM-based microcontrollers: the **STM32 Nucleo-L4R5ZI-P** (based on the **ARM Cortex-M4** core) and the **Raspberry Pi Pico** (based on the dual-core **ARM Cortex-M0+**). These are commonly used platforms in wearable devices and serve as excellent baselines.

  Experiment Description: For the STM32 and Pico platforms, we ported our C-code implementation and measured key performance metrics for both the epilepsy and AF detection case studies. This included:

  - **Computational Demand/Latency:** Inference time per data window.
  - **Power Consumption:** Active and idle power draw measured during operation.
  - **Estimated Battery Life:** Calculated using the same methodology as the X-HEEP platform (based on a 480 mAh battery) for a direct comparison.

  Results Summary: Our evaluation across the three platforms was analyzed separately for each case study, yielding a clear picture of the performance and efficiency trade-offs. We have updated the main manuscript and replaced the original hardware performance table with these two new tables containing the comprehensive comparison.

  - **Epilepsy Detection Case Study** For the epilepsy detection task, the results show different strengths for each platform.
    * **Latency:** In low-latency mode, the X-HEEP and STM32 Nucleo delivered comparable performance, with inference times of 1.9s and 1.67s, respectively. The Raspberry Pi Pico was slower, with a latency of 3.75s.

Table 1: Performance comparison for the **Epilepsy Detection** application across the X-HEEP, STM32 Nucleo, and Raspberry Pi Pico platforms. The metrics are measured for a single inference on a 12-second EEG signal window.

Platform	Mode	Frequency [MHz]	Active power [mW]	Idle power [mW]	Voltage [V]	Exec. time [seconds]	Battery life [hours]
**X-HEEP**	low-latency	450	50	18.7	1.2	1.9	24.3
	low-power	75	3.7	-	0.8	12	103.8
**STM32 Nucleo**	low-latency	120	132	77.5	1.8	1.673	10.2
	low-power	16	35.5	27.9	1.8	11.62	24.5
**RPi Pico**	low-latency	133	82.3	23.7	3.3	3.752	37.7
	low-power	41	38.7	18.8	3.3	12	40.9

Table 2: Performance comparison for the **AF Detection** application across the X-HEEP, STM32 Nucleo, and Raspberry Pi Pico platforms. The metrics are measured for a single inference on a 10-second ECG signal window.

Platform	Mode	Frequency [MHz]	Active power [mW]	Idle power [mW]	Voltage [V]	Exec. time [seconds]	Battery life [hours]
**X-HEEP**	low-latency	450	50	18.7	1.2	0.76	27.3
	low-power	34.5	1.9	-	0.8	10	202.1
**STM32 Nucleo**	low-latency	120	124	77.5	1.8	4.41	8.8
	low-power	45	62	44.5	1.8	9.9	14.0
**RPi Pico**	low-latency	133	80.4	23.7	3.3	4.527	32.1
	low-power	60	47	19.8	3.3	10	33.7

    * **Energy Efficiency & Battery Life:** The X-HEEP platform is the clear leader in efficiency. In its low-power mode, it achieves an estimated battery life of 103.8 hours. This is more than double the best baseline result of the Raspberry Pi Pico (40.9 hours) and four times longer than the STM32 (24.5 hours).

- AF Detection Case Study For the AF detection task, the X-HEEP platform was the dominant performer, leading in both speed and energy efficiency by a significant margin.
    * **Latency:** The X-HEEP was exceptionally fast in low-latency mode, with an inference time of only 0.76 seconds. This is nearly 6 times faster than the STM32 (4.41s) and Raspberry Pi Pico (4.53s).
    * **Energy Efficiency & Battery Life:** The X-HEEP's efficiency is most apparent here. Its low-power mode delivers an estimated 202.1 hours of battery life. This is approximately 6 times longer than the baseline platforms, which both operate for around 33 hours in their most efficient configurations. This performance is driven by X-HEEP's remarkably low active power of 1.9 mW in this mode.

Discussion of Trade-offs: These new results allow for a clearer discussion of hardware trade-offs. The X-HEEP platform, with its highly configurable RISC-V architecture and specialized low-power mode (0.8V operation), is optimized for maximum energy efficiency. In contrast, the general-purpose STM32 Nucleo and Raspberry Pi Pico offer standardized, widely accessible development environments based on ARM Cortex-M4 and -M0+ cores, respectively.

The trade-off is evident within each case study: one platform/mode may offer lower latency while another provides better battery life. This highlights that the optimal platform/mode choice depends on whether the primary application constraint is raw speed or power consumption.

Importantly, while the choice of hardware impacts computational performance, the core benefit of the MetaWearS update mechanism—updating a tiny prototype instead of a large model—remains

a constant and significant advantage across all platforms, with energy savings of 418x-456x due to reduced data transmission.

Manuscript Addition: We have expanded the hardware evaluation discussion in the manuscript to include these new platforms, metrics, and a more explicit discussion of the trade-offs. This information has been added to **Section 2.4 ("Battery life with MetaWearS")**. The added subsection 2.4.1 is highlighted below:

> **Hardware Platform Trade-offs**
>
> To provide a comprehensive evaluation of MetaWearS's performance and applicability, we extended our analysis beyond the custom RISC-V-based X-HEEP platform. We deployed and benchmarked our system on two widely-used, commodity hardware platforms: the STM32 Nucleo-L4R5ZI-P, which features an ARM Cortex-M4 core, and the Raspberry Pi Pico, built on a dual-core ARM Cortex-M0+. These platforms represent standard choices for embedded and wearable device development, allowing for a direct comparison between a specialized, energy-centric RISC-V implementation and mainstream ARM architectures.
>
> For both the epilepsy and AF detection case studies, we measured three key metrics: inference latency (execution time), power consumption (active and idle), and estimated battery life. To ensure a fair comparison, battery life for all platforms was calculated based on a standard 480 mAh battery.
>
> The complete performance results for the epilepsy detection task are detailed in Table 1. The analysis reveals a clear trade-off between latency and energy efficiency. In their respective low-latency modes, the STM32 Nucleo achieves the fastest inference time at 1.67 seconds, closely followed by the X-HEEP at 1.9 seconds. However, in terms of energy efficiency, the X-HEEP platform is the undisputed leader. Its low-power mode yields an estimated battery life of 103.8 hours, which is over four times longer than the STM32's 24.5 hours and more than double the Raspberry Pi Pico's 40.9 hours.
>
> For the AF detection application, as shown in Table 2, the X-HEEP platform demonstrates superior performance across both latency and efficiency. In its low-latency mode, it completes an inference in just 0.76 seconds—nearly 6× faster than both the STM32 Nucleo (4.41s) and the Raspberry Pi Pico (4.53s). This efficiency advantage is even more pronounced in the low-power mode, where the X-HEEP achieves an estimated battery life of 202.1 hours. This is approximately 6× longer than the baselines, which both offer around 33 hours. This remarkable efficiency is primarily due to X-HEEP's extremely low active power draw of only 1.9 mW in this configuration.
>
> These results highlight the distinct advantages of each architectural approach. The X-HEEP platform, with its configurable RISC-V core and specialized features like its ultra-low-voltage (0.8V) operating point, is explicitly optimized for energy-efficient, long-duration monitoring tasks. In contrast, the ARM-based STM32 and Raspberry Pi Pico provide highly accessible, general-purpose development ecosystems. The choice of hardware is therefore dictated by the specific requirements of the target application: for applications demanding the lowest possible latency, the STM32 Cortex-M4 proves competitive, whereas for those prioritizing maximum battery life, the specialized RISC-V architecture of X-HEEP is unparalleled.
>
> Crucially, this expanded evaluation demonstrates that while the underlying hardware platform influences execution time and power draw, the core principle of MetaWearS—updating a compact prototype to avoid transmitting large raw data or full models—delivers substantial energy savings universally. The 418× to 456× reduction in energy consumption from minimized data transmission is a platform-independent benefit that underscores the robustness of our approach.

We believe these additions, including experimental results from a more standard hardware platform, significantly strengthen the practical demonstration of MetaWearS and address the reviewer's comments on trade-offs.

- In my personal opinion, the organization of the paper should be revised. I'm not sure if this is a choice to align with the structure of other articles of this target journal or if it is a choice of the authors.

However, I had to read section 4 to fully understand sections 3 and 2. I would have preferred a more traditional organization such as Abstract, Intro, SoA/related works and background info, methods, results, discussion and conclusion. In particular, "methods" before and not after the "results" would have helped better understand the results themselves. Some of my comments may be caused by having lost information due to the paper's organisation.

We thank the reviewer for their constructive suggestion on the paper's structure. We agree that presenting the methods before the results would significantly enhance the paper's clarity, and we intend to make this change.

For the sake of simplicity during this revision stage, we have kept the current section order to ensure that all references in our responses correspond directly to the manuscript. However, we consider this feedback very important. If the paper is recommended for acceptance, we will restructure the manuscript as suggested for the final version: Abstract 1.Intro → 2.Methods → 3.Results → 4.Discussion → 5.Conclusion → Data/Code/Refs.

Some additional comments:

- Figure 6 is unclear - can authors improve the caption and the in-text comment? What is the red line? What are the points? Etc.
- p.5 at the beginning "... convention approach one..." is the word convention correct? Or was it conventional? What is approach one? I apologize. I do not understand the sentence and the reference.
- The choice of colour for the test set points in plot 9a is not very helpful (being red and purple very similar).

We thank the reviewer for these additional comments, which help us improve the manuscript's presentation.

Regarding Figure 6 clarity: We apologize for the previous lack of clarity. We have revised both the figure caption and the in-text commentary (Section 2.2.2) to provide a clear explanation of the box plot elements. The red line represents the median AUC improvement, the box spans the interquartile range (IQR), the whiskers extend to 1.5x IQR (or data extremes if no outliers), and each individual point represents the result of an experiment with a specific patient combination. The revised caption now explicitly details these elements:

> Figure 6: Box plots illustrating the distribution of AUC improvement for MetaWearS compared to the conventional source-only approach for (a) epileptic seizure detection and (b) AF detection across multiple experimental runs with different patient combinations from the target dataset. The red line indicates the median improvement, the box spans the interquartile range (IQR), the whiskers extend to 1.5x IQR from the box boundaries (or data extremes if no outliers are present), and individual points beyond the whiskers represent outliers from specific experimental runs.

Concerning the typo on p.5 (now in Section 2.2.2):
We thank the reviewer for highlighting this unclear sentence and sincerely apologize for the typo. The reviewer is correct; the word "convention" should have been "conventional". The confusing phrase "...conventional approach one" was an imprecise reference to our first baseline experiment, which is designed to address Challenge ①. We recognize this was poorly worded and confusing for the reader. To resolve this issue and improve clarity, we have substantially revised Section 2.2.2. We have removed the vague "conventional approach" terminology and replaced it with the specific, descriptive title "Source-Only Baseline." As now clearly defined in the text, this baseline refers to a standard transfer learning model that uses a conventional classifier. It is trained on the base dataset and tested directly on the target dataset, without using the prototypical network architecture of our proposed MetaWears model The revised caption now explicitly details these elements:

> To evaluate MetaWearS in comparison with a standard transfer learning strategy, we establish a Source-Only Baseline. This baseline utilizes a model with the same backbone architecture as

MetaWearS, but it is trained for direct classification using a conventional classifier. This approach addresses Challenge (1)—the limited data in the target dataset—by training a model on a larger base (source) dataset and then directly evaluating its performance on the target dataset without any fine-tuning or prototype-based classification. This direct transfer method is common in medical research across various data types, such as medical images[20-23], biomedical signals[24-29], electronic health records [30-33], and their combinations [34-37]. This section compares the performance of MetaWearS against this Source-Only Baseline to specifically measure the performance improvement gained from our fine-tuning stage. To ensure this comparison was robust and accounted for patient-specific variability, we conducted multiple experiments using different combinations of patients from the target dataset, obtaining corresponding results for both MetaWears and the Source-Only Baseline in each run. The AUC improvement using MetaWearS for each target dataset was then calculated by subtracting the conventional approach's result from the MetaWearS result.

Regarding the color choice in Figure 9a: We thank the reviewer for this observation and agree that the original color choice was not optimal. We have revised Figure 9a and its legend to use a new color scheme with clearly distinguishable colors and markers for the training set, test set, and prototypes to enhance readability and avoid any confusion(Figure 5).

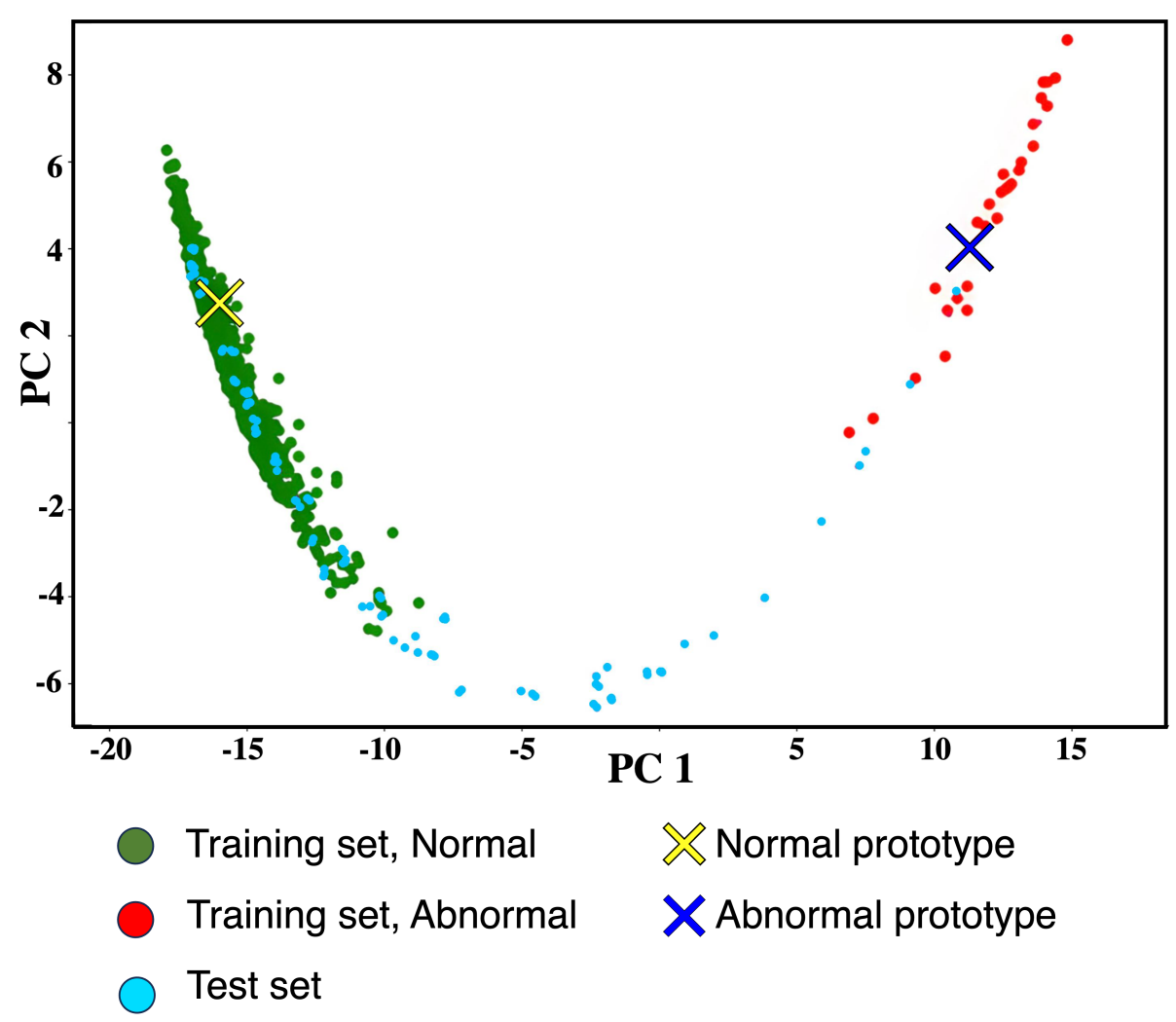

Figure 5: Epileptic seizure detection

A meta-learning method with reduced data requirements for training and updating deep learning models on wearable devices

We are writing to resubmit our revised manuscript titled "MetaWearS: A Shortcut in Wearable Systems Lifecycle with Only a Few Shots" (Manuscript ID: COMMSMED-24-0848-T.) for publication in Nature Communications medicine.

We are delighted to submit our revised manuscript and are thrilled that the reviewers have recommended the publication of our work in Communications Medicine. We sincerely thank the Editor and the Reviewers for their time and expertise; we found their feedback to be extremely constructive and appreciate the opportunity to bring our work to the high standards of the Nature Portfolio.

Based on the Editor's comments and the journal's specific policy requirements, we have implemented the necessary structural and formatting changes to the manuscript. We believe that these revisions have significantly enhanced the paper's clarity, rigor, and overall contribution. The revised manuscript is attached with this letter, and all modifications and additions made to the original text have been highlighted in yellow for ease of identification.

Sincerely,

Alireza Amirshahi, Maedeh H. Toosi, Siamak Mohammadi, Stefano Albini, Pasquale Davide Schiavone, Giovanni Ansaloni, Amir Aminifar, and David Atienza

In accordance with the editorial guidance and policy requirements, we have revised the manuscript to adhere to the journal's specific formatting requests. These changes include updating the title, adjusting the Abstract, Plain Language Summary, and Methods sections.

- Manuscript Title

  We have updated the title to "A meta-learning method with reduced data requirements for training and updating deep learning models on wearable devices." We confirm this title matches both the manuscript file and the submission system.

- Abstract

  Conclusions Our findings demonstrate that the proposed method effectively overcomes the critical barriers of data scarcity and energy constraints, offering a practical and efficient solution for the lifecycle management of deep learning models on wearable health devices.

- Plain Language Summary

  We have included a Plain Language Summary immediately following the Abstract. It uses non-specialist language to explain the study's aim, methods, main findings, and significance.

  Plain language summary Wearable devices are essential for continuous health monitoring, but introducing a new device presents significant challenges. Training an artificial intelligence (AI) model for the new device typically requires collecting large amounts of medical data specifically from that new hardware. Furthermore, updating these models over time requires continuous data collection and significantly drains the device's battery. To address these barriers, we developed a method that allows a new device to drastically reduce the amount of data needed for initial training and subsequent updates. In tests for detecting epileptic seizures and irregular heart rhythms, our method achieved high accuracy with very few samples and reduced the energy required for updates by over 400 times. This technology could accelerate the deployment of new health monitors.

- Placement of Methods

  We have moved the Methods section to immediately follow the Introduction.

- Figure Formatting

  We have reviewed all figures and converted graphs depicting single point values with error bars into box-plots overlaid with individual data points to better visualize the data distribution. Please note that due to these revisions and the reorganization of the manuscript, the figure numbering has been updated. For example, the results previously displayed in Figure 5 (as a bar plot) are now presented in Figure 7 (as a box-plot). We have also provided the source data underlying these graphs in the Supplementary Data file.

- Table Formatting

  We have reviewed all tables in the manuscript and removed bold or italic formatting from the data cells to ensure a clean presentation. In instances where specific formatting was necessary (e.g., to indicate statistical significance), we have clearly defined its meaning.

- Acknowledgements

  Acknowledgements: This work was supported in part by the Swiss NSF, grant no. 10.002.812: "Edge-Companions: Hardware/Software Co-Optimization Toward Energy-Minimal Health Monitoring at the Edge". Additionally, this work has been partially supported by the Swedish Research Council and the Wallenberg AI, Autonomous Systems and Software Program.

- Author Contributions

  Author Contributions: Conceptualization: A.Amirshahi, M.H.Toosi, S.Mohammadi, A.Aminifar, D.Atienza; Methodology: A.Amirshahi, M.H.Toosi; Software Implementation: A.Amirshahi (Epileptic seizure detection), M.H.Toosi (AF detection); Hardware Implementation: S.Albini, P.D.Schiavone; Writing – Original Draft: A.Amirshahi, M.H.Toosi; Writing – Review & Editing: All; Supervision: S.Mohammadi,

A.Aminifar, D.Atienza; Funding Acquisition: D.Atienza. All authors reviewed and approved the final manuscript.

Reviewer #3

After reviewing the revised manuscript and the associated rebuttal letter, considering also comments from other reviewers. I appreciate the authors' substantial effort in addressing all reviewer comments. The authors have significantly improved the manuscript by clarifying concepts and adding missing details. They also added novel experimental results (e.g. adding noise on EEG signal) and comparisons with alternative techniques (e.g., MAML, Matching Networks, Relation Networks). The manuscript is well written, the figures are clearer, and the new results make the paper more compelling. The generalization discussion has been clearly expanded and now properly addresses patient variability and the limits of transfer between datasets. As a minor suggestion one sentence acknowledging that results are based on two binary detection tasks would be a reasonable and transparent addition. This is optional refinement; the manuscript is already solid without it. Overall, the revision is strong, thorough, and of high quality.

We thank the reviewer for the positive feedback and the helpful suggestion. As recommended, we have added the following sentence to the Discussion section (Section 4):

> Our experiments focus on two binary detection tasks, epileptic seizure detection and AF detection. Future work may explore whether the observed generalization behavior extends to multi-class or more diverse clinical settings.
