# Supplementary material for: A meta-learning method with reduced data requirements for training and updating deep learning models on wearable devices
Source: Commun Med (Lond). 2026 Jul 31;6:523. doi: 10.1038/s43856-026-01740-1 (PMC13650112; doi:10.1038/s43856-026-01740-1)
Supplement: Supplementary file 2 [file 43856_2026_1740_MOESM2_ESM.pdf]

Supplementary Material

Table S1: Hardware implementation details of the deep learning models for epilepsy and AF detection.

Feature	Epilepsy	AF
Parameters type	Fixed-point (16 bit)	Floating-point (32 bit)
Operations type	Hybrid	Floating-point (32 bit)
Model size	29.2 KB	209 KB
Input size	117 KB	3.9 KB
Intermediate output size	93 KB	146.5 KB
Prototypes size	64 B	512 B

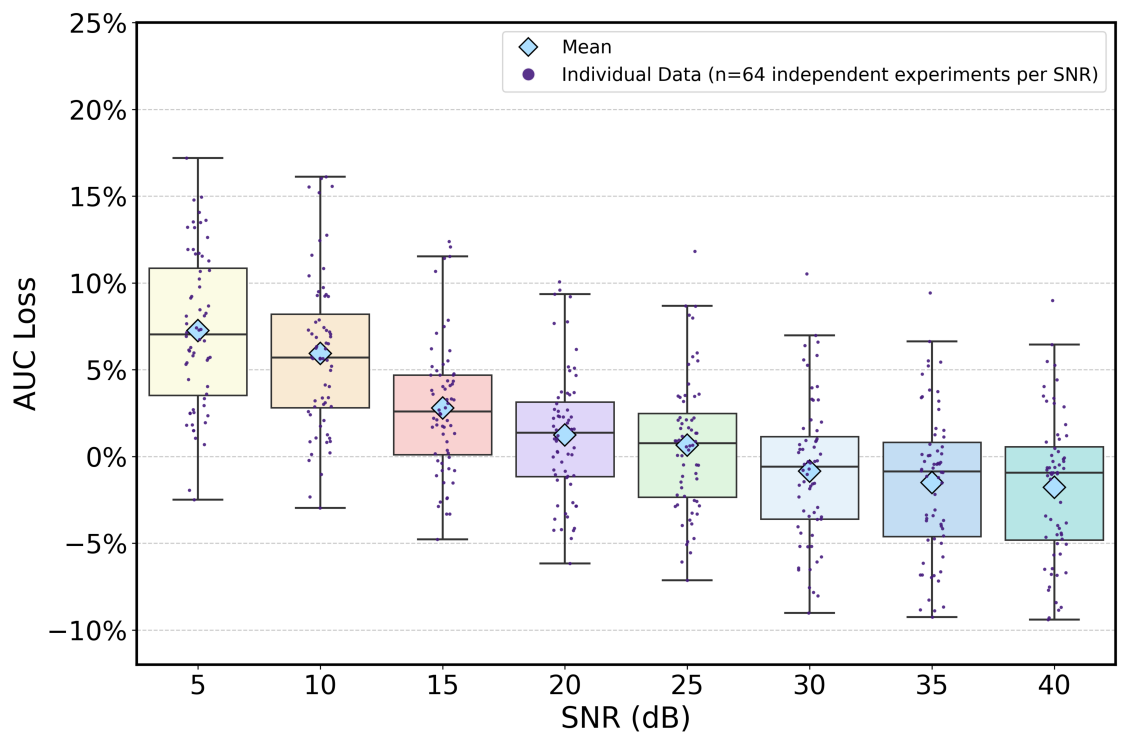

(a) Epilepsy seizure Noise Test

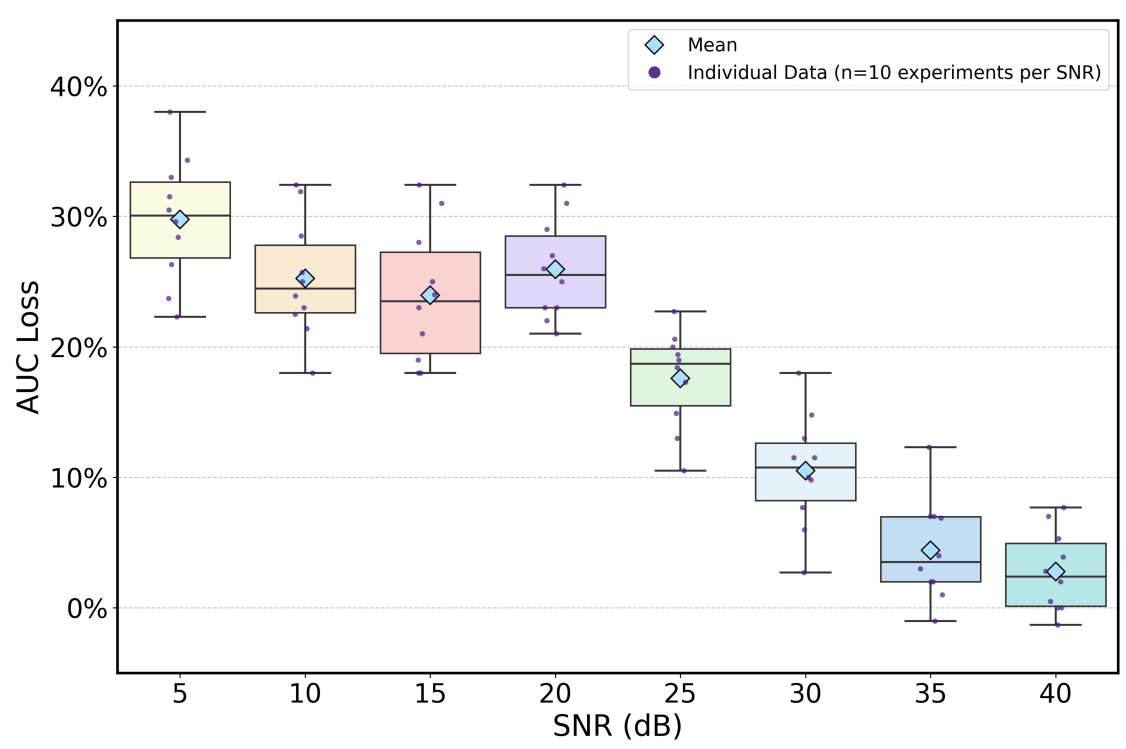

(b) AF Noise Test

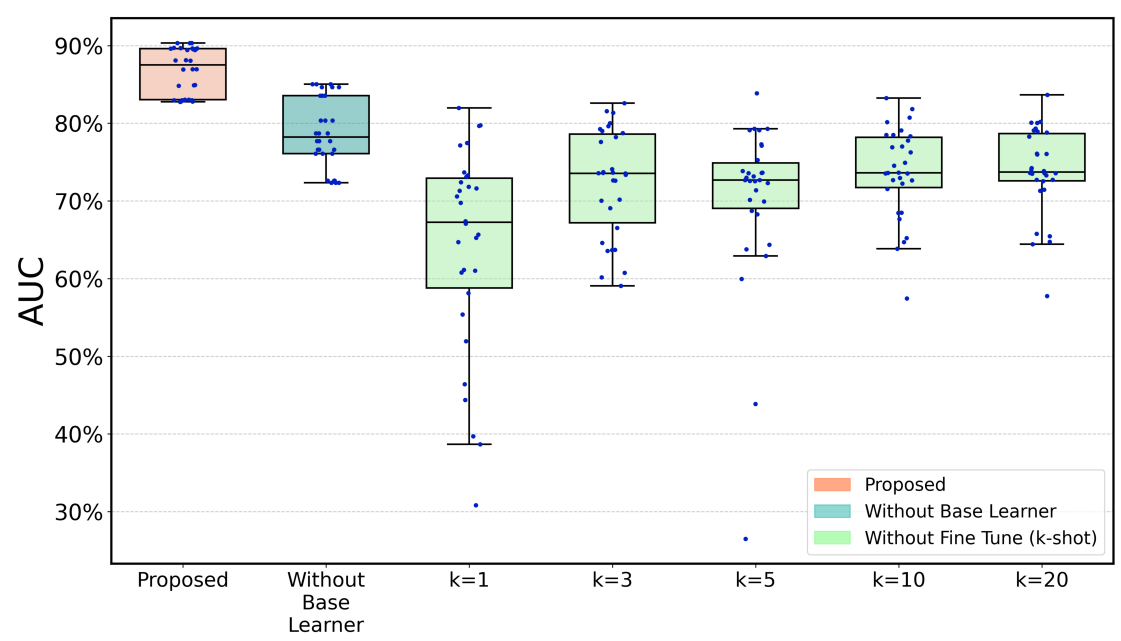

(c) Epilepsy seizure detection

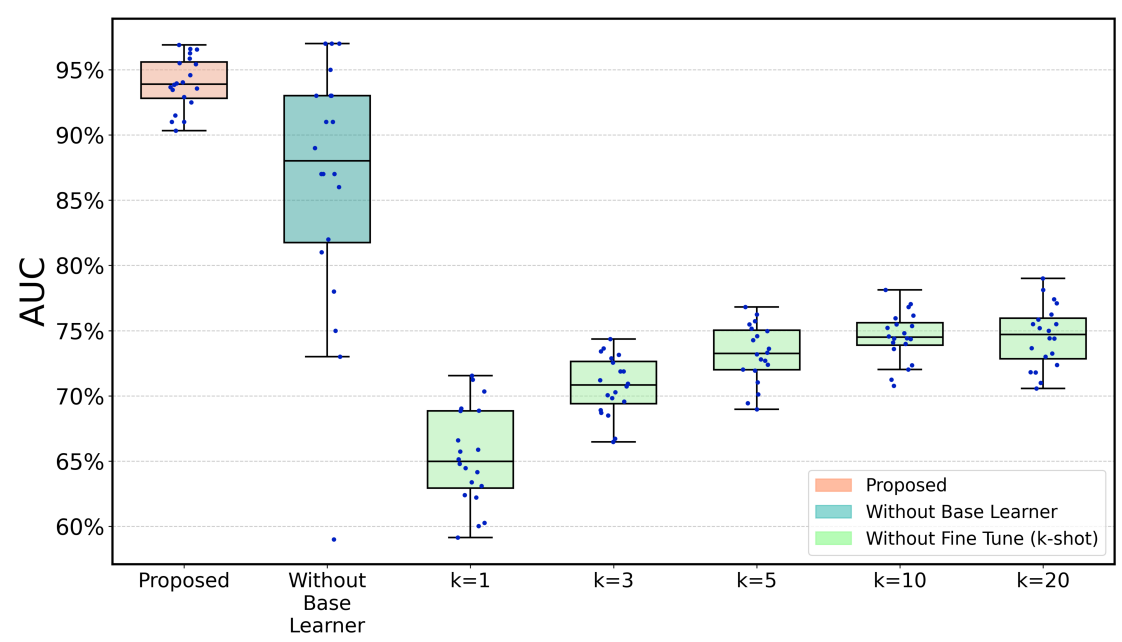

(d) AF detection

Figure S1: Robustness analysis to simulated noise and ablation study of the MetaWearS framework. (a–b) Comparison of performance degradation across different SNR levels for (a) epilepsy and (b) AF. Performance degrades with increasing noise, but MetaWearS maintains reasonable efficacy under common noise conditions. (c–d) Comparison of the results for the two ablation study cases "Without Fine-tune" and "Without Base Learner" and the proposed method MetaWearS for (c) epilepsy and (d) AF, showing the results for different shots 1, 3, 5, 10 and 20 in the case "Without fine tune". For the noise resilience study, the sample sizes are $n = 64$ for epilepsy and $n = 10$ for AF. For all ablation study configurations (Proposed, Without Base Learner, and each column of Without Fine-tune), the sample sizes are $n = 30$ for epilepsy and $n = 20$ for AF.
